# Epidemiological trends in abortion and miscarriage between 1990 and 2019

**DOI:** 10.1186/s12978-025-02049-3

**Published:** 2025-06-05

**Authors:** Yadi Wang, Yujie Tang, Guoshuai Wang, Ran Wei, Lu Liu, Chao Lu

**Affiliations:** 1 Department of Reproductive Medicine, Affiliated Hospital of Shandong Second Medical University, Weifang, Shandong Province 261000 P.R. China; 2 School of Clinical Medicine, Shandong Second Medical University, Weifang, 261000 China

**Keywords:** Global burden of disease, Abortion, Social development index, Disability-adjusted life-years

## Abstract

**Background:**

Abortion and miscarriage both lead to pregnancy loss, with miscarriage occurring spontaneously and abortion referring to the intentional ending of pregnancy. In this context, unsafe remains a major threat to reproductive health. To inform public health strategies and improve maternal health outcomes, it is essential to monitor the epidemiological data associated with abortion and miscarriage. In this study, we utilized data from the Global Burden of Disease Study (GBD) 2019 to comprehensively assess the global burden of abortion and miscarriage from 1990 to 2019.

**Methods:**

The available data were stratified by year, age, and social development index (SDI) at the global, regional, and country levels from the GBD 2019 database. Furthermore, we employed estimated annual percentage changes (EAPCs) to assess the age-standardized rates of abortion and miscarriage. Additionally, linear regression analysis was conducted to examine the relationship between abortion rates and SDI values.

**Results:**

The global incidence of abortion and miscarriage decreased from 49,637,961 in 1990 to 42,385,827 in 2019. The number of deaths dropped significantly from 59,475 to 19,565, while disability-adjusted life years (DALYs) decreased from 3,449,222 to 1,130,038 over the same period. Despite this overall decline, the disease burden remains substantial in some regions. The low-SDI had the highest age-standardized incidence rate (ASIR), reaching 1,983.8 per 100,000. At the national level, Ethiopia recorded the highest ASIR at 3,839.06 per 100,000 in 2019, followed by Bolivia at 3,524.9 per 100,000. Among 204 countries and territories, Chad (17.59 per 100,000) and Niger (14.02 per 100,000) had the highest age-standardized death rates (ASDR) in 2019. Among the 21 GBD regions, Eastern Sub-Saharan Africa had the highest DALY rate in 2019, amounting to 208.42 per 100,000. Globally, a high incidence cases (13,341,611) was recorded for young women aged 20 to 29 years, meanwhile deaths (3,239) was higher in women aged 35 to 44 years.

**Conclusions:**

By identifying key risk factors and high-burden areas, we hope to provide a usable theoretical basis for policymakers and healthcare professionals to develop targeted interventions that improve reproductive health outcomes.

**Supplementary Information:**

The online version contains supplementary material available at 10.1186/s12978-025-02049-3.

## Plain Language Summary

Abortion and miscarriage both lead to pregnancy loss. Unsafe abortion, in particular, greatly impairs maternal reproductive health. Understanding the burden and epidemiological trends of abortion and miscarriage is essential for informing public health strategies. In this study, we used the Global Burden of Disease Study (GBD) 2019 to comprehensively analyse the burden of abortion and miscarriage from 1990 to 2019. Although our study observed a global decline in incidence from 1990 to 2019, abortion and miscarriage remains significant reproductive health concerns in low-SDI countries. The low-SDI had much greater age-standardized rates of incidence, deaths and DALYs than the other SDI quintiles. Globally, a high incidence was observed for women aged 20 to 29 years, and a greater number of deaths was recorded for women aged 35 to 44 years.

Our study may provide useful theoretical data for reproductive health interventions. Studying the epidemiology of abortion and miscarriage, identifying key risk factors and regional disparities, can enable healthcare systems to implement targeted interventions to protect reproductive health effectively.

**Supplementary Information:**

The online version contains supplementary material available at 10.1186/s12978-025-02049-3.

## Background

Abortion and miscarriage have the same outcome— pregnancy loss, both of them are involved the separation of an embryo or a fetus from the mother’s uterus before it is mature [1]. Abortion is defined as elective or medically indicated termination of pregnancy at any gestational age [2]. The term abortion is commonly used to indicate an induced abortion, meaning a medication or procedure to end the pregnancy, while the term miscarriage is used to indicate a spontaneous abortion. According to Bearak’s findings, six out of ten unintended pregnancies end in induced abortion [3].

Miscarriage is a spontaneous loss of early pregnancy, which often occurs in the first 20 weeks of pregnancy and occupies about 15–20% of maternal pregnancy complications [4, 5]. The aetiology and pathogenesis of miscarriage are unclear. Approximately 50% of miscarriages are caused by chromosomal abnormalities [6]. Patients with complete spontaneous abortions rarely require intervention. Women with incomplete miscarriages require medical or surgical interventions. Early abortion with a combination of mifepristone and misoprostol has replaced some surgical abortions, as it has no long-term adverse effects on health or fertility [7, 8].

However, not all abortions are safe. Bleeding and infection are common complications of abortion, and septic abortion is a major cause of death [9]. The WHO defines unsafe abortion as “a procedure for terminating an unintended pregnancy either by individuals without the necessary skills or in an environment that does not conform to minimum medical standards or both” [10]. Unsafe abortion has been a serious public health issue in recent years. Approximately 14% of pregnancy-related deaths worldwide are attributed to complications due to unsafe abortion [11]. According to a study detailing the sociodemographic structure and general distribution of abortion incidence, over half (56%) of abortions among Nigerian women are caused by unplanned pregnancies, and the majority of abortions are unsafe [12]. Zane reported that between 1998 and 2010, the mortality rate associated with legal induced abortion was 0.7 deaths per 100,000 procedures in the United States [13]. A study revealed that laws restricting abortion increase inequality in the incidence of abortion. For example, total maternal mortality increased by 7% in states with policies limiting abortion in the United States from 2015 to 2018 [14, 15].

The above data are on the incidence and mortality of abortions over a period of several years, with studies focusing on specific countries and regions. To our knowledge, less comprehensive global-scale analysis has been conducted to describe the epidemiological characteristics and burden of incidence and mortality associated with abortion and miscarriage. Unsafe abortion greatly impairs maternal reproductive health. The sustainable Development Goal (SDG) 3.7 calls for the widespread distribution of sexual education and improved reproductive health care by 2030 [16]. A comprehensive global assessment provides a broader perspective on overall trends, regional disparities, and potential areas for intervention.

This study aims to quantify the global burden and regional heterogeneity of abortion and miscarriage from an epidemiological perspective. By identifying key risk factors and high-burden areas, we hope to provide a usable theoretical basis for policymakers and healthcare professionals to develop targeted interventions that improve reproductive health outcomes.

## Methods

### Data sources

Our data were extracted from the GBD 2019 database, which integrates multiple primary data sources to generate comprehensive global health estimates. The complete dataset is publicly accessible on the Global Health Data Exchange (GHDx) website (http://GHDx.healthdata.org/GBD-results-tool) [17, 18]. This open-access website collects epidemiological data on 369 diseases and health losses caused by 87 risk factors [19]. The GBD 2019 study described indicators of disease burden, including incidence, death, disability-adjusted life-years (DALYs), and age-standardized rates (ASRs) [20, 21]. Our research topics are categorized as abortion and miscarriage under the broad category of maternal disorders.

### Study variables and modelling approach

The GBD research team found that social development status is closely associated with population health outcomes. DALYs comprehensively consider the loss of life years due to premature death (YLLs) and the loss of healthy life years due to disability (YLDs), reflecting the total burden of diseases on society [17].

SDI values are on a scale from 0 to 1, the value of 0 indicates that lagged distribution has the lowest per-capita income, the average education years for those aged 15 years and over is the lowest, and the total fertility rate of the population under 25 years is the highest [17, 22]. SDI is defined using a quintile-based classification, categorizing countries into five groups: high, high-middle, middle, low-middle, and low. In our study, the 21 GBD regions are standard categorizations used within the GBD framework to assess geographic and socioeconomic disparities in health outcomes. For the analysis by income level, we selected representative countries or groups from the GBD regional classification based on their income characteristics. This approach allows us to explore differences in abortion and miscarriage burden across diverse economic contexts while maintaining consistency with the GBD methodology.

The number of cases in this paper is the total number of cases in the cohort containing all ages, while the ASRs are rates after standardizing for age. The ASRs (per 100,000 population) were calculated as described in a previous article [23]. The natural logarithm of the ASR exhibits a linear relationship with time and is calculated as Y = α + βX + ε. In this formula, Y denotes$\:\:{ln}\left(AsR\right)$, X denotes the calendar year, and ε denotes the error term. Based on the original data from the GBD, the estimated annual percent changes (EAPCs) for abortion and miscarriage globally, regionally, and in 204 countries and territories were calculated using relative statistical software. The EAPC was calculated as 100 × (exp(β) − 1), and the 95% confidence interval (CI) of the EAPC was derived from the linear model [24, 25]. Positive and negative values of the EAPC reflect trends in the global burden of abortion and miscarriage. If both the EAPC and the lower limit of its 95% CI were > 0, the age-standardized rate showed an increasing trend. In contrast, if the EAPC and the upper limit of its 95% CI were < 0, the age-standardized rates presented a decreasing trend.

### Statistical analysis

We assessed the association between ASRs and SDI values using Pearson’s correlation analysis. Statistical analyses were performed using R software (version 4.2.1) and GraphPad Prism 9.0. *P* < 0.05 was considered to indicate a significant difference.

## Results

### The global burden of abortion and miscarriage in 204 countries and territories

Globally, the number of reported incident cases declined from 49,637,961 (95% uncertainty interval (UI): 37,364,984–63,774,614) in 1990 to 42,385,827 (95%UI: 32,538,623–53,751,065) in 2019, representing a 15% reduction (95%UI: 9–19%) (Table 1). The age-standardized incidence rate (ASIR) decreased from 1703.56 (95%UI: 1288.59–2179.36) to 1098.36 (95%UI: 843.01–1391.51) per 100,000 population over three decades, with an estimated annual percentage change (EAPC) of -1.44% (95% CI: -1.49%, -1.40%) (Table 1). The upper limit of the 95% CI was below 0, confirming a significant downward trend in ASIR. A total of 19,565 (95%UI: 16,319–23,373) deaths occurred worldwide in 2019, marking a 67% (95%UI: 61–73%) reduction compared to 59,475 (95%UI: 51,678–68,097) in 1990 (Table S1). The global age-standardized death rate (ASDR) in 2019 was 0.5 (95%UI: 0.41–0.59) per 100,000 population, with an EAPC of -5.21% (95%CI: -5.38%, -5.04%), showing a decreasing trend from 1990 to 2019 (Table S1). In 2019, abortion and miscarriage accounted for 1,130,038 (95%UI: 947,675–1,338,397) DALYs globally, marking a 67% decline (95%UI: 61–73%) since 1990. The age-standardized DALY rate was 28.86 (95%UI: 24.21–34.15) per 100,000 population, with an EAPC of -5.14% (95%UI: -5.31%, -4.96%), indicating a sustained decrease over three decades (Table S2).

**Table 1:** The incidence cases and age-standardized rates of maternal abortion and miscarriage in 1990 and 2019, and their temporal changes from 1990 to 2019, for female, in global, SDI regions and 21 GBD regions

Location	1990 Number (95% UI)	2019 Number (95% UI)	Change of number from 1990 to 2019 (95% UI)	1990 ASIR (per100,000) (95% UI)	2019 ASIR (per100,000) (95% UI)	EAPC in ASIR (%, 95% CI)
Global	49,637,961 (37364984–63774614)	42,385,827 (32538623–53751065)	-0.15 (-0.19–0.09)	1703.56 (1288.59-2179.36)	1098.36 (843.01-1391.51)	-1.44 (-1.49–1.4)
Low SDI	6,973,740 (5256837–9009711)	11,637,902 (8822761–15031690)	0.67 (0.62–0.73)	2755.25 (2106.01–3539)	1983.8 (1519.8-2558.48)	-1.05 (-1.17–0.92)
Low-middle SDI	11,410,130 (8524369–14790897)	10,494,835 (7838015–13604841)	-0.08 (-0.12–0.04)	1927.33 (1444.94-2493.41)	1075.64 (805.82-1390.98)	-1.96 (-2–1.93)
Middle SDI	16,958,086 (12370288–22212792)	11,997,853 (9245783–15119393)	-0.29 (-0.35–0.22)	1673.45 (1246.38-2186.33)	993.6 (764.48-1250.2)	-1.68 (-1.79–1.58)
High-middle SDI	10,042,815 (7463420–12970993)	6,017,285 (4776984–7495964)	-0.4 (-0.47–0.31)	1587.98 (1181.16-2046.6)	904.47 (710.64-1116.01)	-1.83 (-2.02–1.65)
High SDI	4,221,596 (3472966–5082058)	2,209,475 (1778026–2748701)	-0.48 (-0.53–0.41)	1007.14 (828.67-1208.39)	502.02 (404.72-618.15)	-2.81 (-3.03–2.59)
Central Asia	828,596 (593960–1105493)	781,569 (570200–1043319)	-0.06 (-0.14-0.03)	2194.31 (1587.96-2917.78)	1589.35 (1154.08-2114.26)	-0.77 (-1.04–0.51)
Central Europe	310,038 (230687–402651)	188,959 (143335–253242)	-0.39 (-0.5–0.27)	541.62 (398.59-712.17)	388.47 (292.29-507.63)	0.34 (-0.05-0.74)
Eastern Europe	2,059,621 (1538534–2697902)	1,281,790 (956431–1705000)	-0.38 (-0.47–0.26)	1955.61 (1446.94-2590.15)	1384.82 (1037.2-1822.72)	-0.84 (-1.07–0.61)
Australasia	106,203 (76407–140865)	96,730 (71130–127403)	-0.09 (-0.23-0.09)	1005.61 (721.97-1335.03)	738.9 (543.51-970.54)	-0.8 (-0.96–0.64)
High-income Asia Pacific	1,375,473 (1099004–1707700)	301,895 (243413–383894)	-0.78 (-0.82–0.73)	1602.32 (1279.43-1992.6)	413.09 (332.38-524.11)	-7.11 (-8.05–6.15)
High-income North America	1,142,786 (991252–1327219)	576,199 (488901–681395)	-0.5 (-0.54–0.44)	813.13 (703.84–942.1)	359.47 (305.88-424.06)	-2.83 (-3.11–2.54)
Southern Latin America	139,537 (104134–182133)	127,760 (97018–164309)	-0.08 (-0.2-0.06)	545.28 (408.12-707.95)	383.07 (290.87-491.64)	-1.23 (-1.46–1.01)
Western Europe	1,515,164 (1150236–1945036)	1,270,044 (987208–1621564)	-0.16 (-0.26–0.03)	795.79 (606.41-1019.49)	706.06 (548.79-897.13)	-0.09 (-0.23-0.05)
Andean Latin America	869,202 (663509–1098257)	869,019 (673750–1096062)	0 (-0.08-0.09)	4108.38 (3175.16-5186.27)	2578.46 (2001.01-3253.24)	-1.52 (-1.55–1.48)
Caribbean	527,128 (388366–680341)	358,847 (270939–458761)	-0.32 (-0.37–0.26)	2536.28 (1883.51-3276.97)	1495.33 (1129.37-1910.45)	-1.86 (-1.91–1.8)
Central Latin America	2,680,776 (1973815–3499753)	2,350,893 (1785885–2930404)	-0.12 (-0.19–0.06)	2786.42 (2078.65-3601.09)	1735.13 (1319.37-2162.77)	-1.54 (-1.67–1.41)
Tropical Latin America	1,797,177 (1316630–2369899)	1,527,795 (1116733–1973642)	-0.15 (-0.23–0.07)	2039.52 (1499.73-2683.38)	1319.07 (964.48-1703.05)	-1.32 (-1.39–1.24)
North Africa and Middle East	4,303,738 (3203543–5578771)	3,829,589 (2904840–4937884)	-0.11 (-0.18–0.03)	2433.58 (1849.67-3144.08)	1180.71 (893.91-1521.84)	-2.53 (-2.58–2.48)
South Asia	9,349,633 (6837491–12377996)	9,314,408 (6771633–12457913)	0 (-0.06-0.06)	1679.5 (1243.37-2212.56)	928.34 (676.29-1243.13)	-1.89 (-1.97–1.8)
East Asia	12,095,212 (8617458–16002093)	5,237,452 (4163818–6391050)	-0.57 (-0.62–0.49)	1557.56 (1119.09-2060.71)	806.07 (634.77–985.1)	-2.34 (-2.74–1.93)
Oceania	34,726 (26201–44779)	57,845 (43255–76262)	0.67 (0.52–0.82)	993.78 (756.3-1273.98)	813.02 (610.71-1063.89)	-0.67 (-0.7–0.64)
Southeast Asia	3,836,492 (2837830–5051369)	2,845,632 (2180767–3588592)	-0.26 (-0.31–0.2)	1403.88 (1051.82-1831.03)	808.8 (619.62-1021.14)	-1.8 (-1.88–1.72)
Central Sub-Saharan Africa	477,197 (361214–619069)	777,994 (590824–1009273)	0.63 (0.5–0.78)	1801.53 (1373.37-2347.16)	1176.77 (898.41-1532.1)	-1.38 (-1.55–1.22)
Eastern Sub-Saharan Africa	3,765,853 (2841735–4891872)	5,996,056 (4511106–7781668)	0.59 (0.52–0.67)	4036.31 (3085.02-5224.92)	2735.43 (2065.94-3542.31)	-1.44 (-1.55–1.33)
Southern Sub-Saharan Africa	507,413 (385169–660959)	779,238 (592324–1030599)	0.54 (0.38–0.69)	1766.54 (1350.24-2291.86)	1741.58 (1322.78-2291.39)	0.23 (-0.11-0.58)
Western Sub-Saharan Africa	1,915,996 (1456166–2485815)	3,816,111 (2920945–4979935)	0.99 (0.89–1.07)	2056.21 (1578.9-2646.59)	1602.35 (1234.08-2088.8)	-0.72 (-0.83–0.62)

*Abbreviations*: *ASIR* age-standardized incidence rate, *EAPC* estimated annual percentage change, *CI* confidence interval, *UI* uncertainty interval

**Fig. 1:**
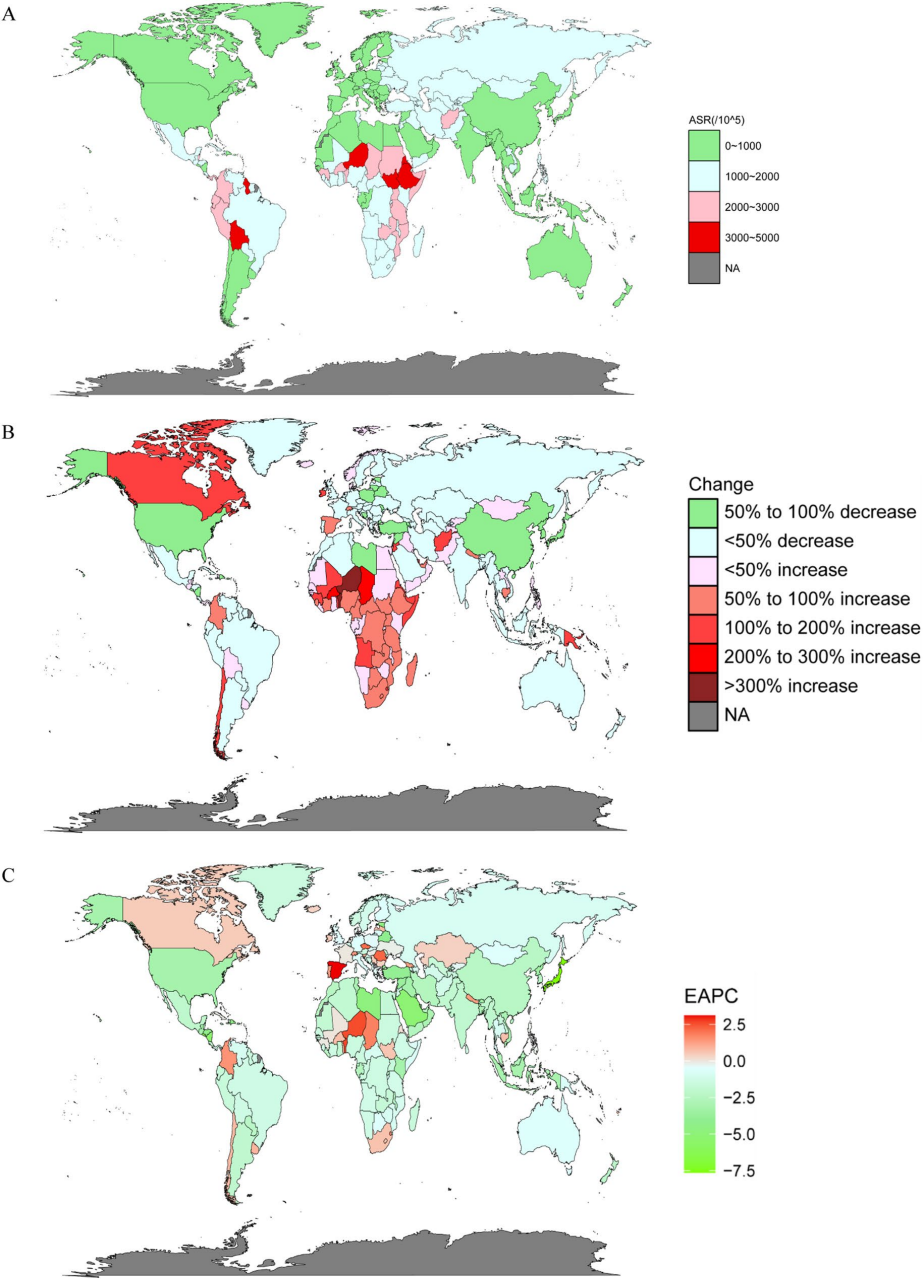
The incidence of abortion and miscarriage in 204 countries and territories. **A** The age-standardized incidence rate (ASIR) of abortion and miscarriage in 2019. **B** The percent change in incidence cases of abortion and miscarriage from 1990 to 2019. **C** The estimated annual percentage change (EAPC) of abortion and miscarriage in ASIR from 1990 to 2019

In 2019, the highest ASIR of abortion and miscarriage was in Ethiopia (3839.06 (95%UI: 2900.13–4987.39) per 100,000 population), followed by Bolivia (Plurinational State of) (3524.897 (95%UI: 2622.06–4530.11) per 100,000 population), and Niger (95%UI: 3293.98 (95%UI: 2469.62–4304.95) per 100,000 population) (Fig. 1A; Table S3). Singapore (60.13 (95%UI: 50.48–71.26) per 100,000 population) had the lowest ASIR in 2019, followed by Brunei Darussalam (148.03 (95%UI: 112.05–192.71) per 100,000 population), and Republic of Korea (182.97 (95%UI: 133.21–250.89) per 100,000 population) (Fig. 1A; Table S3). The largest change in incident cases was in Niger (365% (95%UI: 288–433%)) from 1990 to 2019, while the greatest decrease in incident cases was in Japan (-80% (95%UI: -84%, -75%)) (Fig. 1B; Table S3). Moreover, Japan (-7.63% (95%CI: -8.67%, -6.58%)) showed the greatest decrease by the EAPC in ASIR (Fig. 1C; Table S3).

In 2019, Chad had the highest ASDR at 17.59 per 100,000 (95%UI: 12.09–23.84), followed by Niger at 14.02 per 100,000 (95%UI: 9.2–19.75) (Figure S1A; Table S4). Chad also showed the largest increase in deaths, rising by 109% (95%UI: 37–201%), while the number of deaths decreased by 100% (95%UI: 99–100%) in Bosnia and Herzegovina from 1990 to 2019 (Figure S1B; Table S4). The greatest reduction in the EAPC of ASDR was also observed in Bosnia and Herzegovina (-20.13% (95%CI: -21.76%, -18.47%)) (Figure S1C; Table S4). Chad (958.79 (95%CI: 658.31–1286.7) per 100,000 population) had the highest age-standardized DALY rate in 2019 (Figure S2A; Table S5). DALYs in Chad increased by 112% (95%UI: 39–205%), while deaths in Bosnia and Herzegovina declined by 98% (95%UI: 98–99%) over 30 years (Figure S2B; Table S5). During the same period, Bosnia and Herzegovina (-15.12% (95% CI: -16.89%, 13.31%)) showed the most significant decreasing trend in the EAPC of DALYs (Figure S2C; Table S5). In 1990, Chile had the lowest ASIR at 207.3372 (95%UI: 153.0–281.12) per 100,000 population. Ethiopia (5569.06 (95%UI: 4202.62–7310.2) per 100,000 population) and Bolivia (5250.99 (95%UI: 3909.47–6829.99) per 100,000 population) had the highest ASIRs in 1990 (Figure S3A; Table S3). Ethiopia also had the largest ASDR at 35.04 per 100,000 (95%UI: 26.28–47.66) in 1990 (Figure S3B; Table S4). And the highest age-standardized DALY rate were observed in Timor-Leste (1537.44 (95%UI: 1033.28–2053.91) per 100,000 in 1990 (Figure S3C; Table S5).

Overall, the burden of abortion and miscarriage exhibited a decreasing trend at the global level. Among the 204 countries and territories assessed in 2019, Ethiopia, Niger, and Chad had heavy abortion and miscarriage burdens.

### Trends of ASIRs, ASDRs and age-standardized DALY rates by sociodemographic index (SDI)

**Fig. 2:**
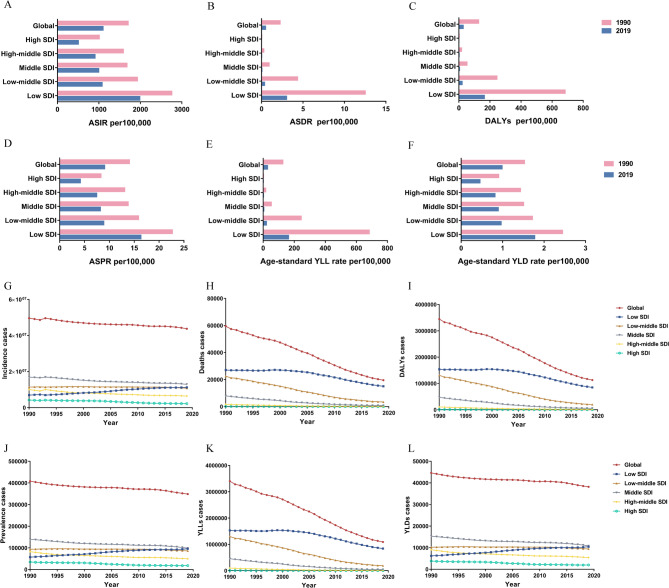
Trends of ASIRs, ASDRs and age-standardized DALY rates by sociodemographic index (SDI). The age-standardized incidence (**A**), death (**B**), DALY (**C**), prevalence (**D**), YLL (**E**), YLD (**F**) rate of abortion and miscarriage by year (1990 and 2019). Trends in the number of incidence (**G**), death (**H**), DALY (**I**), prevalence (**J**), YLL (**K**), YLD (**L**) cases of abortion and miscarriage from 1990 to 2019. DALY: disability-adjusted life-year; YLL: year of life lost; YLD: year lived with disability

We performed a statistical analysis by SDI. Compared to that in 1990, the disease burden in 2019 was lower in each SDI quintile. In 2019, the largest ASIR was in low-SDI at 1983.8 (95%UI: 1519.8–2558.48) per 100,000 population, while the high-SDI had the lowest ASIR 502.02 (95%UI: 404.72–618.15) per 100,000 population (Fig. 2A; Table 1). In 2019, the ASIR in the low-middle-SDI was 1075.64 (95%UI: 805.82–1390.98) per 100,000 population, which was very close to the global level of 1098.36 (95%UI: 843.01–1391.51) per 100,000 population (Table 1). The ASDRs were lower than the global level at 0.5(95%UI: 0.41–0.59) per 100,000 population in four SDI quintiles, except in the low-SDI (3.03(2.47–3.73) per 100,000 population) in 2019 (Fig. 2B; Table S1). The low-SDI recorded the highest ASR of DALY among all SDI quintiles in both 1990 (684.62 (95%UI: 571.18–815.38) per 100,000 population) and 2019 (165.56 (95%UI: 134.76–201.11) per 100,000 population) (Fig. 2C; Table S2). Low-SDI had the highest age-standardized prevalence (ASPR) both in 1990 (22.67 (95%UI: 14.47–33.39) per 100,000) and 2019 (16.32 (95%UI: 10.43–24.11) per 100,000) (Fig. 2D)., In 2019, the age-standardized YLL rate had significantly decreased across all SDI quintiles compared to 1990, with high-SDI having the lowest age-standardized YLL rate at (0.35 (95%UI: 0.3–0.41) per 100,000 population (Fig. 2E). Low-SDI had the highest age-standardized YLD rates in both 1990 (2.45 (95%UI: 1.3–3.94) per 100,000) and 2019 (1.78 (95%UI: 0.94–2.87) per 100,000) (Fig. 2F).

From 1990 to 2019, most SDI levels showed downward trends in incident cases, with the exception of the low-SDI (Fig. 2G). The most significant decrease in deaths occurred in the low-middle SDI, with a reduction of 19,030 people (Fig. 2H; Table S1). The most significant decline in DALY was also seen in the low-middle SDI (Fig. 2I). The slight increase in prevalent cases was observed in low-SDI from 1990 to 2019 (Fig. 2J). Low-SDI was also had the highest YLLs over the 30 years (Fig. 2K). Additionally, an increasing trend in YLDs was seen in low-SDI during this period (Fig. 2L).

### Trends of ASIRs, ASDRs and age-standardized DALY rates in the 21 GBD regions

**Fig. 3:**
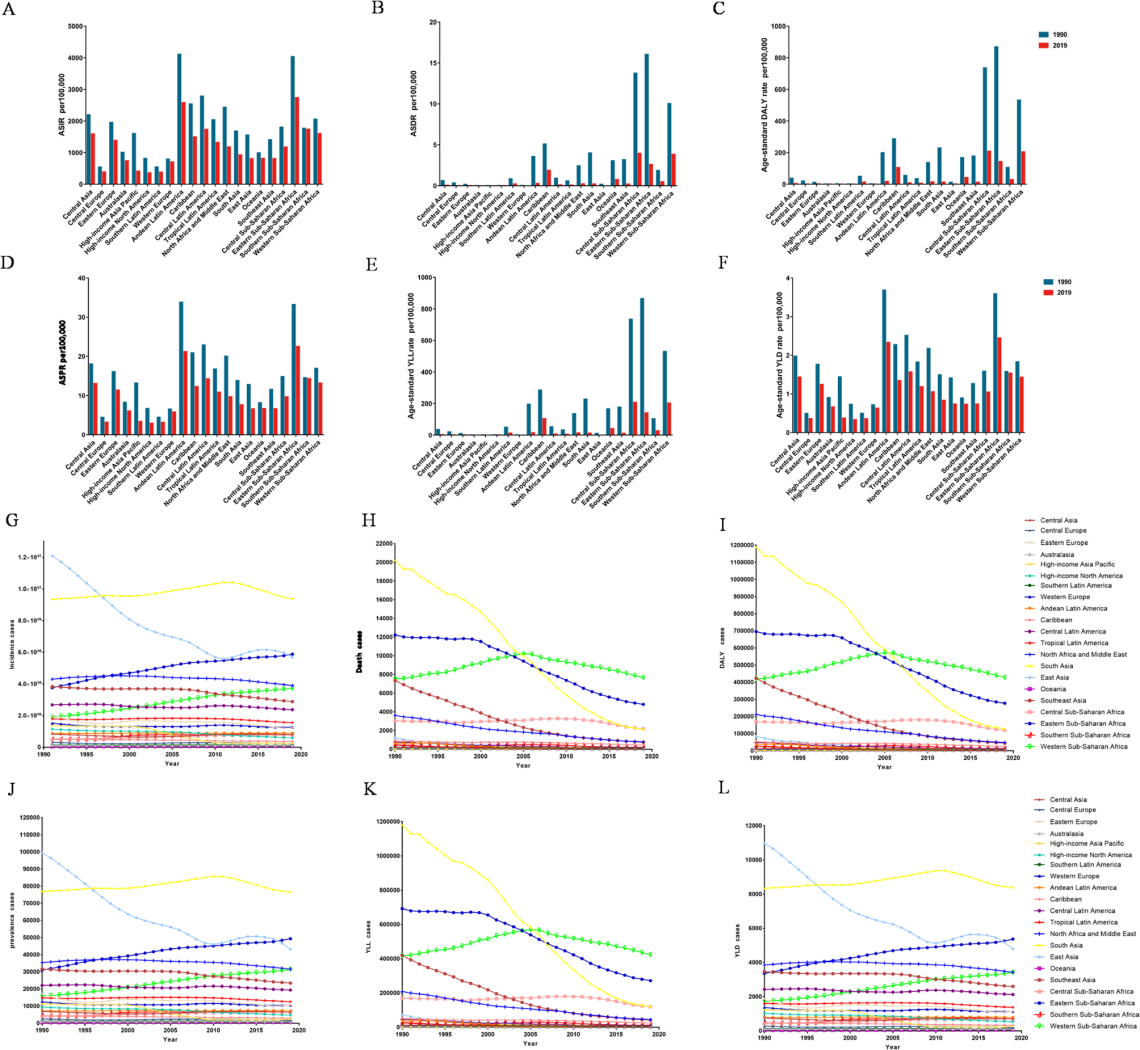
Trends of ASIRs, ASDRs and age-standardized DALY rates in the 21 GBD regions. The age-standardized incidence (**A**), death (**B**), DALY (**C**), prevalence (**D**), YLL (**E**), YLD (**F**) rate of abortion and miscarriage compared between 1990 and 2019. Trends in the number of incidence (**G**), death (**H**), DALY (**I**), prevalence (**J**), YLL (**K**), YLD (**L**) cases of abortion and miscarriage from 1990 to 2019. DALY: disability-adjusted life-year; YLL: year of life lost; YLD: year lived with disability

In 2019, eastern Sub-Saharan Africa had the highest ASIR (2735.44 (95%UI: 2065.94–3542.31) per 100,000 population), while North America had the lowest ASIR (359.47 (95%UI: 305.88–424.06) per 100,000 population) (Fig. 3A; Table 1). Eastern Sub-Saharan Africa (16.04(95%UI: 12.79–20.29) per 100,000 population) had the highest ASDR in 1990, but Central Sub-Saharan Africa (3.96 (95%UI: 2.88–5.29) per 100,000 population) had the highest ASDR in 2019. The lowest ASDR in 2019 was observed in Australasia, with 0.00156 (95%UI: 0.00118–0.00205) per 100,000 population (Fig. 3B, Table S1). High-income Asia Pacific (0.46 (95%UI: 0.28–0.69) per 100,000 population) had the lowest age-standardized DALY rate in 2019 (Fig. 3C; Table S2). Eastern Sub-Saharan Africa had the highest ASPR in 2019 (22.51(95%UI: 14.37–33.58) per 100,000 population) (Fig. 3D). Central Sub-Saharan Africa had the largest age-standardized YLL rate at 207.38(95%UI: 152.42–273.31) per 100,000 population in 2019 (Fig. 3E). Eastern Sub-Saharan Africa (2.45 (95%UI: 1.28–3.97) per 100,000 population) also had the highest age-standardized YLD rate in 2019 (Fig. 3F).

Over the past 30 years, eastern Sub-Saharan Africa showed the largest increase, followed by western Sub-Saharan Africa, while East Asia had the greatest decline (Fig. 3G). Deaths dropped most notably in South Asia, with Southeast Asia next (Fig. 3H). For DALYs, South Asia experienced the sharpest decline over the 30 years (Fig. 3I). Eastern Sub-Saharan Africa also had the greatest rise in prevalence (Fig. 3J). The largest drop in YLLs was in South Asia (Fig. 3K). For YLDs, the most obvious decreasing trend occurred in East Asia, and the most obvious increasing trend was in eastern Sub-Saharan Africa from 1990 to 2019 (Fig. 3L).

### Trends of ASIRs, ASDRs and age-standardized DALY rates in different income regions

**Fig. 4:**
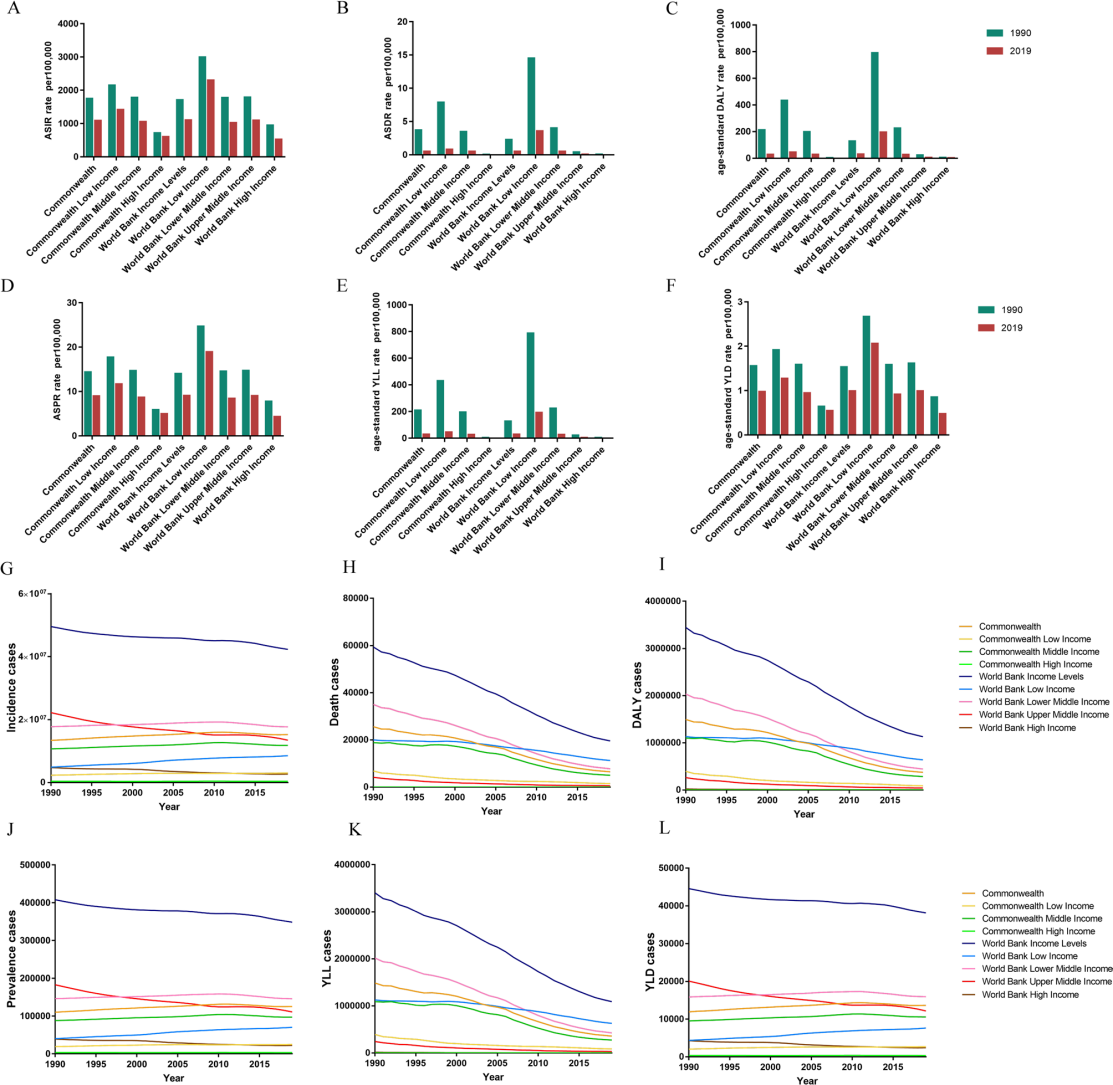
Trends of ASIRs, ASDRs and age-standardized DALY rates in different income areas. The age-standardized incidence (**A**), death (**B**), DALY (**C**), prevalence (**D**), YLL (**E**), YLD (**F**) rate of abortion and miscarriage by year (1990 and 2019). Trends in the number of incidence (**G**), death (**H**), DALY (**I**), prevalence (**J**), YLL (**K**), YLD (**L**) cases of abortion and miscarriage from 1990 to 2019. DALY: disability-adjusted life-year; YLL: year of life lost; YLD: year lived with disability

We further analysed abortion and miscarriage burden by income level using the Commonwealth and World Bank classifications from the GBD 2019 study. In both systems, low-income area had the highest ASIRs in 1990 and 2019, while high-income area had the lowest (Fig. 4A). Similarly, low-income countries also showed the highest age-standardized rates for deaths, DALYs, prevalence, YLLs, and YLDs, whereas high-income area had the lowest rates (Fig. 4B–F).

From 1990 to 2019, the World Bank upper middle-income countries showed the greatest decline in incident cases (Fig. 4G). Deaths in the lower middle-income group exceeded those in the low-income group until 2009, after which the low-income group reported the highest death numbers (Fig. 4H). After 1998, prevalent cases in upper middle-income areas dropped below those in lower middle-income area (Fig. 4J). High-income countries consistently had the lowest YLLs over the three decades (Fig. 4K). A sustained decline in YLDs was observed only in the World Bank upper middle-income areas (Fig. 4L).

### Trends of ASIRs, ASDRs and age-standardized DALY rates by age group

**Fig. 5:**
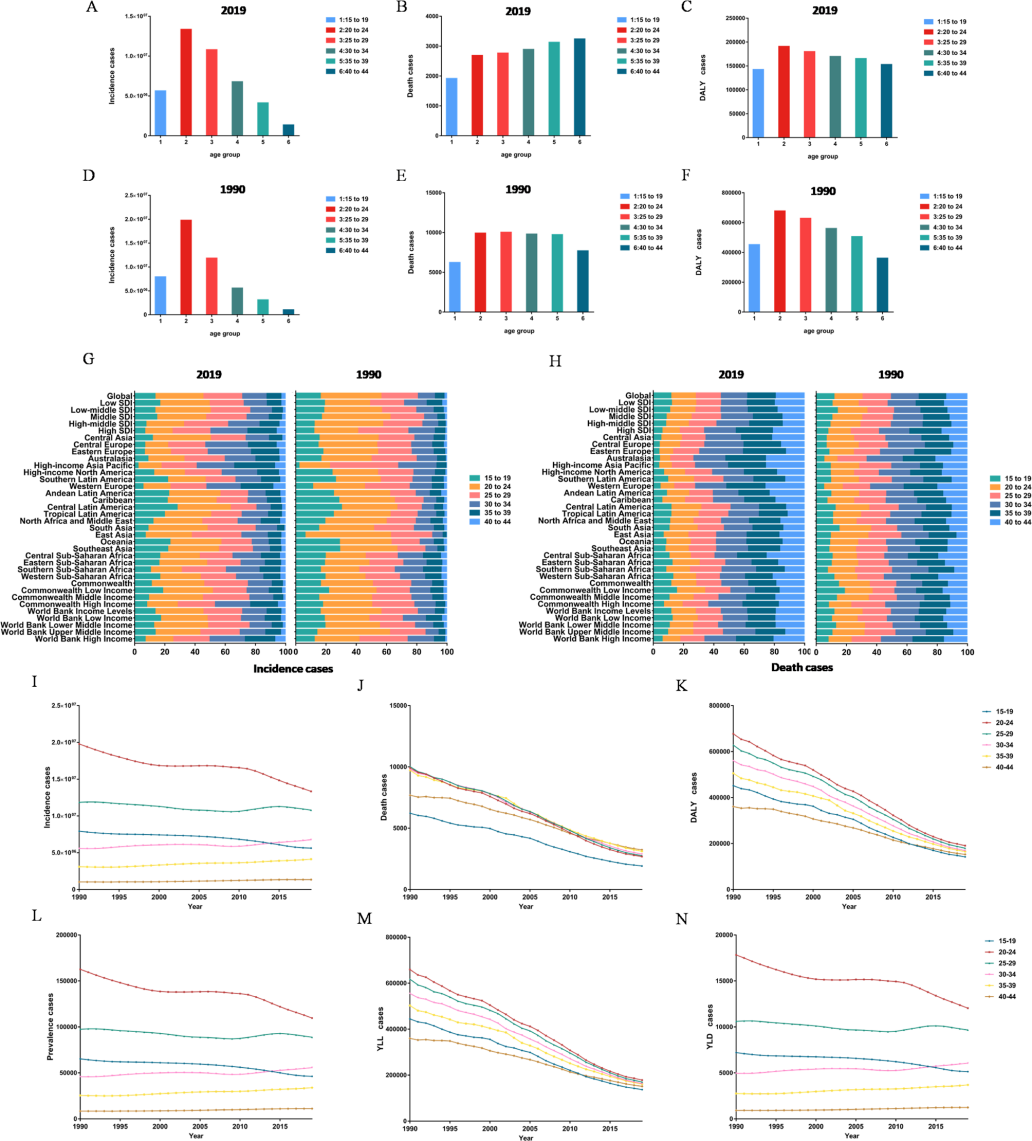
Trends of ASIRs, ASDRs and age-standardized DALY rates by age group. The populations were divided into six age groups: 15–19 years, 20–24 years, 25–29 years,30–34 years,35–39 years, 40–44 years. The number of incidence (**A**), death (**B**), DALY (**C**) cases of abortion and miscarriage by age groups in 2019. The number of incidence (**D**), death (**E**), DALY (**F**) cases of abortion and miscarriage by age groups in 1990. The proportion (%) of the six age groups in the total incidence cases (**G**). The proportion (%) of the six age groups in the total death cases (**H**). Trends in the number of incidence (**I**), death (**J**), DALY (**K**), prevalence (**L**), YLL (**M**), YLD (**N**) cases of abortion and miscarriage by six age groups from 1990 to 2019. DALY: disability-adjusted life-year; YLL: year of life lost; YLD: year lived with disability

We extracted data from people aged 15–44 years and divided them into six age groups: 15–19, 20–24, 25–29, 30–34, 35–39, and 40–44 years. Compared to 1990, the numbers of incident cases, deaths, and DALYs were lower in all age groups in 2019 (Fig. 5A-F). We found that the 20–24 years group had the largest number of incident cases in both 2019 (13,341,611(95%UI: 8,819,863–18,764,757)) and 1990 (1,028,981(95%UI: 706,394–1,550,370)), suggesting the importance of prenatal screening in younger women (Fig. 5A and D). The number of incidences was the lowest in the 40–44 years (1,352,441(95%UI: 951,622–1,985,526)), but the number of deaths was high in 2019 (3239(95%UI: 2,598–3,988)) (Fig. 5B). We also observed the heaviest DALYs among those females aged 20 to 24 years in 2019 (190,785(95%UI: 155,903–233,177)) (Fig. 5C). In 1990, the 15–19 years group (180(95%UI: 149–219)) had the least number of deaths (Fig. 5E). The 20–24 years group (677,573(95%UI: 548,039–833,442)) had the greatest number of DALY cases in 1990, which may be related to the poor medical treatment (Fig. 5F). The prevalences, YLLs and YLDs are shown in Supplemental Fig. 4.

In 2019, the highest proportion of incident cases occurred in the 25–29 years group in countries among high-middle SDI (29.17%), such as Australasia (26.67%). And the 20–24 years group had the largest proportion of incident cases in the low-SDI (32.69%) and low-middle SDI (36.72%), including countries like Central Latin America (35.24%) and South Asia (39.15%) (Fig. 5G). Regarding the total number of deaths, the proportion of people aged 35–39(18.81%) and 40–44(19.49%) is higher globally in 2019 (Fig. 5H).

From 1990 to 2019, incident cases declined in the 15–19, 20–24, and 25–29 age groups, with the 20–24 years group showing the fastest decrease. In contrast, cases gradually increased in the 30–34, 35–39, and 40–44 groups. The 15–19 and 30–34 years groups had equal numbers of incident cases in 2014, afterward, the 30–34 years group had a greater number of cases than the 15–19 years group (Fig. 5I). During this period, deaths decreased across all age groups, with the most significant decline in the 20–24 group (Fig. 5J). DALYs also dropped, especially in the 25–29 years group (Fig. 5K). After 2014, the prevalence in the 30–34 years group was greater than that in the 15–19 years group (Fig. 5L). Although the 25–29 years group showed a general downward trend, it had a greater number of YLLs than the other groups each year (Fig. 5M). In addition, the number of YLDs in the 20–24 years group decreased fastly (Fig. 5N).

### Risk factors for abortion and miscarriage

**Fig. 6:**
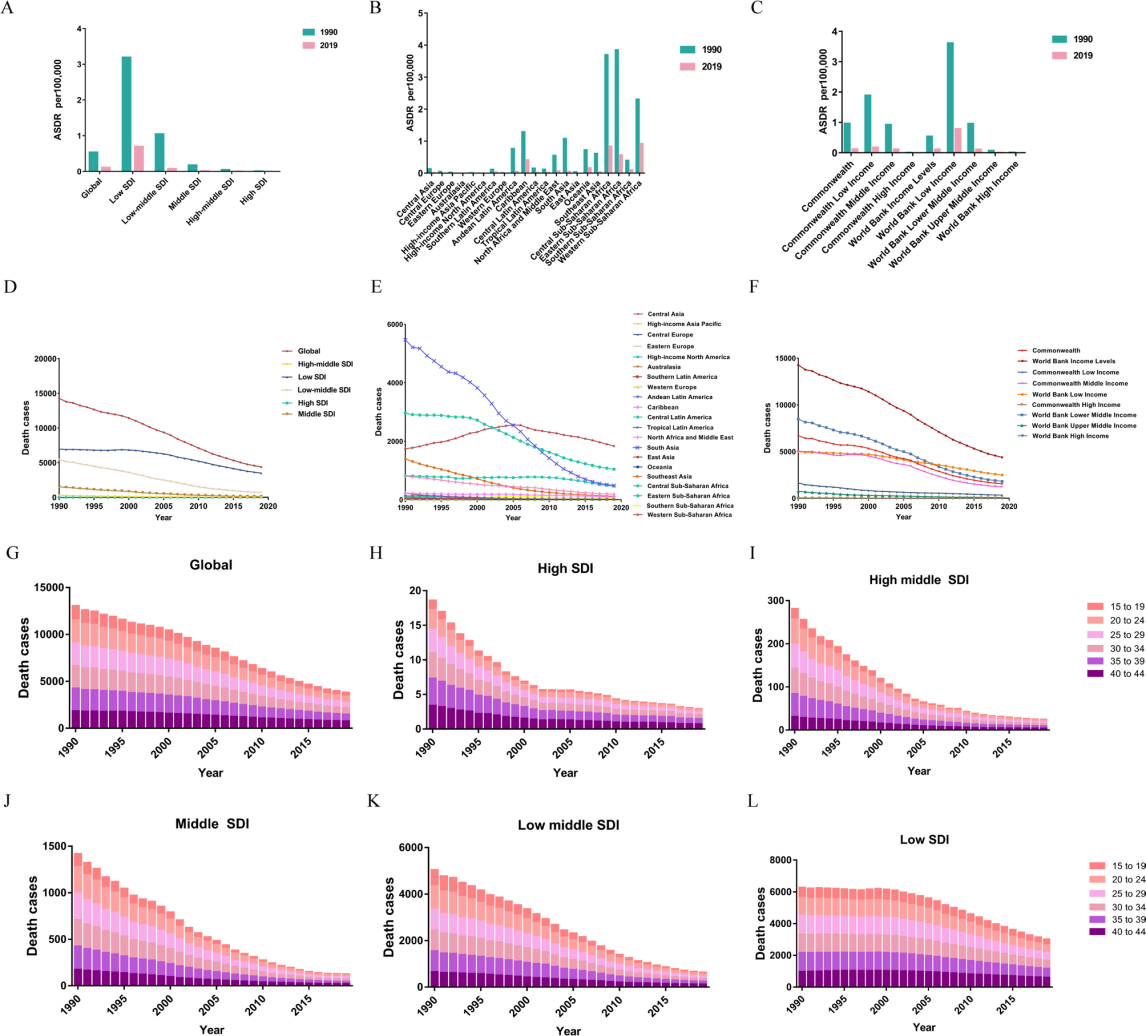
The death burden attributable to risk factor (iron deficiency) for abortion and miscarriage. **A** The age-standardized death rate (ASDR) of abortion and miscarriage attributable to iron deficiency globally and in five SDI quintiles in 2019. **B** The age-standardized death rate (ASDR) of abortion and miscarriage attributable to iron deficiency in 21GBD regions in 2019. **C** The age-standardized death rate (ASDR) of abortion and miscarriage attributable to iron deficiency in different income areas in 2019. **D** Trends in the number of deaths of abortion and miscarriage attributable to iron deficiency globally and in five SDI quintiles from 1990 to 2019. **E** Trends in the number of deaths of abortion and miscarriage attributable to iron deficiency in 21GBD regions from 1990 to 2019. **F** Trends in the number of deaths of abortion and miscarriage attributable to iron deficiency in different income areas from 1990 to 2019. The proportion of the six age groups for iron deficiency-related deaths globally (**G**), and in high (**H**), high middle (**I**), middle (**J**), low middle (**K**), and low SDI quintiles (**L**) in 2019

According to data collected by the GBD 2019 study, iron deficiency is a subclass of child and maternal malnutrition in the behavioural risk category. From 1990 to 2019, global ASDR of abortion and miscarriage due to iron deficiency declined. Among SDI quintiles, the low-SDI had the highest ASDR at (0.7(95%UI: 0.24–1.19) per 100,000 population) in 2019 (Fig. 6A). Among the 21 GBD regions, deaths from iron deficiency significantly decreased, with western Sub-Saharan Africa (0.91(95%UI: 0.31–1.58) per 100,000 population), central Sub-Saharan Africa (0.84(95%UI: 0.3–1.47) per 100,000 population) and eastern Sub-Saharan Africa (0.56(95%UI: 0.2–0.95) per 100,000 population) showing the highest ASDRs in 2019 (Fig. 6B). In 2019, low-income countries had the highest ASDRs, while high-income countries were the lowest (Fig. 6C).

Globally, deaths due to iron deficiency dropped rapidly, with the sharpest decline in the low-middle SDI group (Fig. 6D). From 1990 to 2006, the number of deaths in western Sub-Saharan Africa showed an upward trend, surpassing the number of deaths in eastern Sub-Saharan Africa by 2002, and the number of deaths in western Sub-Saharan Africa decreased after 2007 (Fig. 6E). Low-income had more higher numbers of deaths in 2019 (Fig. 6F).

We selected the global level and five SDI quintiles to examine the temporal trends in deaths due to iron deficiency by age group. Overall, the proportion and number of deaths declined in all age groups globally and across the five SDI quintiles from 1990 to 2019 (Fig. 6). The 15–19 years group exhibited the lowest total number of deaths per year (Fig. 6G). Among SDI quintiles, the high-SDI had the most rapid decrease and consistently had the lowest death counts (Fig. 6H). In high-middle SDI, deaths were lowest in the 15–19 group, followed by the 40–44 group (Fig. 6I). The distribution of deaths among the age groups in the middle-SDI exhibited a similar pattern to that in the high-middle-SDI, but the number of people in each age group was approximately 5 times that in the high-middle-SDI (Fig. 6J). Low-SDI had the highest death counts across all age groups and showed relatively minor change over time (Fig. 6K).

### The correlation between the SDI and the age-standardized rate of abortion and miscarriage

**Fig. 7:**
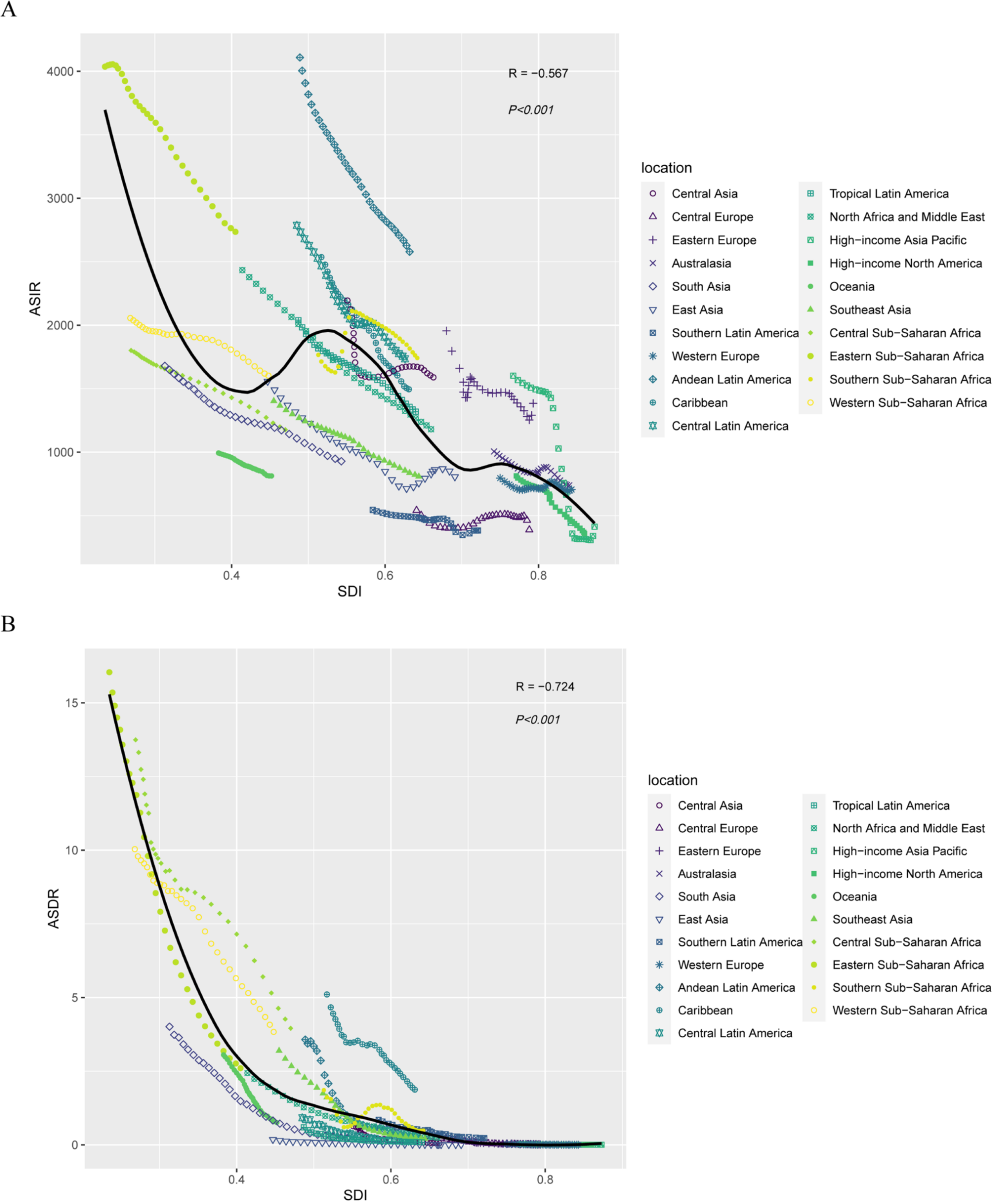
The correlation between the sociodemographic index (SDI) and the age-standardized rate of abortion and miscarriage for 21 GBD regions, 1990–2019. **A** Age-standardized incidence rate (ASIR). **B** Age-standardized death rate (ASDR). Lines in the same colour and pattern represent the age-standardized rate of a region, each point in a line represents 1 year starting at 1990 and ending at 2019. The black line represents the average expected relationship between SDI and age-standardized rates for abortion and miscarriage, according to values for each geographic region from 1990–2019

We investigated the association between the SDI and the age-standardized rate of abortion and miscarriage. Negative correlations were observed between the SDI and the ASIR (ρ = -0.567; *P* < 0.001) or the ASDR (ρ = -0.724; *P* < 0.001) (Fig. 7).

From 1990 to 2019, ASIR generally declined with increasing SDI. Despite a sharper ASIR drop in low-SDI like eastern Sub-Saharan Africa, the disease burden remained high (Fig. 7A). In contrast, ASIR was consistently low in high-SDI, such as high-income North America and the Asia-Pacific (Fig. 7A). ASDR also declined in many regions, with Sub-Saharan Africa showing both the highest ASDR and a decreasing trend as SDI rose (Fig. 7B). However, ASDR remained largely unchanged in some middle- and high-SDI, including Central Europe, Australia, and high-income North America (Fig. 7B).

## Discussion

Reducing unsafe abortion is an important challenge for public health organizations. In the present study, we analysed the spatial and temporal trends in the burden of abortion and miscarriage over the last three decades. We estimated that approximately 42,385,827 (95%UI: 32,538,623–53,751,065) cases of abortion and miscarriage occurred worldwide, with an ASIR of 1098.36 (95%UI: 843.01–1391.51) per 100,000 population in 2019. Abortion and miscarriage accounted for 19,565 total deaths and 1.13 million DALYs in 2019. In general, the total number of deaths due to abortion decreased worldwide from 59,475 (95%UI: 51,678–68,097) in 1990 to 19,565 (95%UI: 16,319–23,373) in 2019. This decline in abortion-related deaths may be attributed to advancements in medical treatment, improved access to healthcare, and increased awareness of women’s reproductive health worldwide. With the increased availability and use of misoprostol and mifepristone, medical abortion has become a safe and convenient method beyond the formal health system and has become more widespread in situations where abortion is restricted by law [26]. The introduction and use of modern contraceptive methods and drugs have reduced the incidence of abortion [27, 28].

When we investigated the relationship between sociodemographic factors and maternal abortion, we found that the incidence and mortality rates were significantly higher in low-SDI countries, such as Sub-Saharan Africa. The main reasons include economic dependence and insecurity [12, 29]. Consistent with other research, this outcome could be due to differences in levels of income or health care in different regions [30]. Disparities in access to healthcare, education, and contraceptive services contribute to higher rates of unintended pregnancies and adverse maternal health outcomes, especially in low-SDI and low-income countries such as Eastern and Western Sub-Saharan Africa. In addition, the strong sense of community and family support in the social and cultural environments shaped by religions, as well as the social stigma associated with abortion, have an impact on abortion. In regions such as the Philippines where Catholicism has a significant influence, some states of the United States under the influence of Christianity, and Thailand influenced by Buddhism, the abortion rates are relatively low [31–33].

Abortion also imposes an economic burden on society. In our study, low-income countries had high ASDRs and DALYs. This result indicates that income is closely related to the disease burden of abortion and miscarriage. Pregnant women must pay for health services to receive an abortion, especially for surgical management, and their families must spend money on nutritional supplements for them [34, 35]. Disparities exist in the availability of contemporary contraceptive and abortion services; for instance, women from low-income countries are at greater risk than women from high-income countries for facing structural and financial obstacles to having an abortion [36]. Studies have shown that as many as 1% and 5% of abortions are unsafe in high-income and upper-middle-income countries, respectively, while the proportions are as high as 20% and 54% in low- and middle-income countries, respectively [37]. Furthermore, the economic costs associated with the incidence of abortion and maternal mortality due to abortion are enormous, imposing burdens on public health care resources and social care services [38]. According to the World Health Organization, almost all abortion-related deaths and disabilities may be avoided by providing sexual education, the use of reliable contraception, access to safe, legally induced abortion, and prompt medical attention for any complications. Therefore, economic estimates may be useful for clinical service and budget planning.

Moreover, age is an important factor contributing to abortion and miscarriage [39, 40]. We found that the 20–29 years group had the highest incidence, and the highest number of deaths occurred in women aged 40–44 years group. This finding suggests the importance of prenatal screening among women at high risk. Women under the age of 20 are disproportionately affected by unsafe abortion practices, exhibiting a higher number of deaths and disabilities compared to women over the age of 20 [41]. An estimated 3.9 million girls aged 15–19 experience unsafe abortions each year [42]. Adolescent girls are often less aware of their rights regarding abortion and postabortion care. Compared to older women, younger girls are more likely to choose self-induced abortion or seek abortion services from non-medical providers.

Iron deficiency was identified as a risk factor for abortion and miscarriage. Lack of iron may cause iron deficiency anaemia, which leads to hypoxia and affects foetal growth and development, resulting in miscarriage [43]. Minerals are essential for human growth, development, and the maintenance of normal physiological functions, and their role in foetal growth and development has received substantial attention [44, 45]. To our knowledge, the risk factors for miscarriage include embryonic chromosomal errors, female endometrial defects, male sperm abnormalities, lifestyle factors, alcohol consumption, environmental pollution, and many other factors [46]. In GBD study, iron deficiency was a major factor for abortion, and deaths due to iron deficiency were an essential part of the disease burden. During pregnancy, the demand for iron increases significantly to maintain maternal erythropoiesis expansion and support fetal growth and development. Studies have shown that in women who experience spontaneous abortion in the first trimester of pregnancy, the concentrations of hepcidin, serum iron, and ferritin are higher than those in women with healthy pregnancies in the first trimester, indicating a close association between miscarriage and changes in maternal iron metabolism [47]. The prevalence of iron deficiency varies among populations in different regions, which is significantly influenced by local lifestyle factors. For example, in some regions, residents have a long-term staple diet of grains. Phytates, oxalates, and other components in these grains can inhibit iron absorption, leading to insufficient iron intake. Moreover, women engaged in occupations such as metal processing and mining, due to long-term exposure to metal dusts or chemical substances, may have their iron metabolism disrupted, increasing the risk of iron deficiency anaemia and thus indirectly raising the likelihood of abortion and miscarriage.

Environmental pollution is also a factor affecting abortion and miscarriage. In Canada, higher concentrations of PM2.5 are associated with an increased incidence of spontaneous abortion [48]. Higher levels of traffic-related air pollution is also linked to miscarriage, and the strongest association is observed between the 10th and 20th weeks of pregnancy [49]. Studies in China have also shown that exposure to PM2.5, PM10, and O_3_ before pregnancy is associated with the risk of pregnancy termination, highlighting the significant impact of pre - pregnancy environmental exposure on adverse pregnancy outcomes [50].

It cannot be ignored that unintended pregnancy is an important cause of abortion. Despite impressive achievements in the use of contraceptives worldwide, family planning is still not effective worldwide [51, 52]. Every year, approximately 33 million women use contraception but still become unintentionally pregnant [53]. The use of contraceptives has reduced the frequency of unwanted pregnancies, but it does not eliminate the need for safe abortion access [3]. Women who experience unintended pregnancies will continue to resort to abortion if the need for family planning is not satisfied. Increasing funding for family planning initiatives to avoid unintended and untimely pregnancies could reduce health care expenses.

However, available data on abortion are limited due to abortion limitations and women’s fear of stigma. Statistical data on abortion can facilitate the comparison of abortion rates among countries. Regarding measurement error, differences in data collection methods and tools across countries can lead to inaccuracies. In resource - scarce countries, under - reporting of abortion and miscarriage cases may occur. For reporting bias, social, cultural, and legal factors cause women and medical institutions in some regions to under - report. As for methodological differences, distinct statistical methods and models used by countries affect data comparability, resulting in potential biases in disease burden estimates. Several challenges exist to improving reproductive health in the SDG era [54]. Unsafe abortion is a clear example of social injustice and inequality throughout the world. The majority of unsafe abortions occur in poor nations, which are also the main countries with severe abortion laws. Efficient screening techniques must be created to track population-based abortion rates and to identify the risks of abortion and miscarriage. As countries expand access to family planning services, the availability of contraception and safe abortion must be improved to address the unintended risk for adolescents in the future.

## Conclusions

The global burden of abortion and miscarriage remains a challenge to reproductive health. Policymakers should focus on the epidemiology of abortion and miscarriage in low SDI and low-income countries such as eastern Sub-Saharan Africa and western Sub-Saharan Africa. Prioritizing the implementation of effective family planning programs and increasing access to contraception are important measures for reducing the number of unintended pregnancies and ultimately decreasing the need for induced abortion.

## Supplementary Information

Supplementary Material 1: Supplementary Figure 1. The mortality of abortion and miscarriage in 204 countries and territories. (A) The age-standardized death rate (ASDR) of abortion and miscarriage in 2019. (B) The percent change in deaths cases of abortion and miscarriage from 1990 to 2019. (C) The estimated annual percentage change (EAPC) of abortion and miscarriage in ASDR from 1990 to 2019.

Supplementary Material 2: Supplementary Figure 2. The disability-adjusted life years (DALYs) of abortion and miscarriage in 204 countries and territories. (A) The DALYs rate of abortion and miscarriage in 2019. (B) The percent change in DALYs of abortion and miscarriage from 1990 to 2019. (C) The estimated annual percentage change (EAPC) of abortion and miscarriage in DALYs from 1990 to 2019.

Supplementary Material 3: Supplementary Figure 3. The global burden of abortion and miscarriage in 204 countries and territories in 1990. (A) The age-standardized incidence rate (ASIR) of abortion and miscarriage in 1990. (B) The age-standardized death rate (ASDR) of abortion and miscarriage in 1990. (C) The age-standardized disability-adjusted life years (DALYs) rate of abortion and miscarriage in 1990.

Supplementary Material 4: Supplementary Figure 4. Trends of prevalence of abortion and miscarriage by age groups. The number of prevalence(A), YLL(B), YLD(C) cases of abortion and miscarriage by age groups in 2019. The number of prevalence(D), YLL(E), YLD(F) cases of abortion and miscarriage by age groups in 1990. DALY: Disability-adjusted life year; YLL: year of life lost; YLD: year lived with disability.

Supplementary Material 5: Supplementary Figure 5. The death burden attributable to risk factor (iron deficiency) of abortion and miscarriage. The age-standardized DALY(A), YLL(B), YLD(C) rate of abortion and miscarriage attributable to iron deficiency globally and in five SDI quintiles in 2019. Trends in the number of DALY(D), YLL(E), YLD(F) cases of abortion and miscarriage attributable to iron deficiency globally and in five SDI quintiles from 1990 to 2019. The age-standardized DALY(G), YLL(H), YLD(I) rate of abortion and miscarriage attributable to iron deficiency in 21 GBD regions in 2019. Trends in the number of DALY(J), YLL(K), YLD(L) cases of abortion and miscarriage attributable to iron deficiency in 21 GBD regions from 1990 to 2019. DALY: Disability-adjusted life year; YLL: year of life lost; YLD: year lived with disability.

Supplementary Material 6: Supplementary Figure 6. The death burden attributable to risk factor (iron deficiency) of abortion and miscarriage. The age-standardized DALY(A), YLL(B), YLD(C) rate of abortion and miscarriage attributable to iron deficiency in different income areas in 2019. Trends in the number of DALY(D), YLL(E), YLD(F) cases of abortion and miscarriage attributable to iron deficiency in different income areas from 1990 to 2019. DALY: Disability-adjusted life year; YLL: year of life lost; YLD: year lived with disability.

Supplementary Material 7.

## Data Availability

The dataset supporting the conclusions of this article is available in the Global Health Data Exchange (GHDx) repository, [unique persistent identifier and hyperlink to dataset in http://ghdx.healthdata.org/gbd-results-tool].

## Abbreviations

WHO: World Health Organization
GBD: Global burden of disease
ASR: Age-standardized rate
ASIR: Age-standardized incidence rate
ASDR: Age-standardized death rate
DALY: Disability-adjusted life year
YLL: Year of life lost
YLD: Year lived with disability
EAPC: Estimated annual percentage change
SDI: Social-demographic index
UI: Uncertainty interval
CI: Confidence interval
