# Supplementary figures and images for: Epidemiological trends in abortion and miscarriage between 1990 and 2019
Source: Reprod Health. 2025 Jun 5;22:97. doi: 10.1186/s12978-025-02049-3 (PMC12143105; doi:10.1186/s12978-025-02049-3)

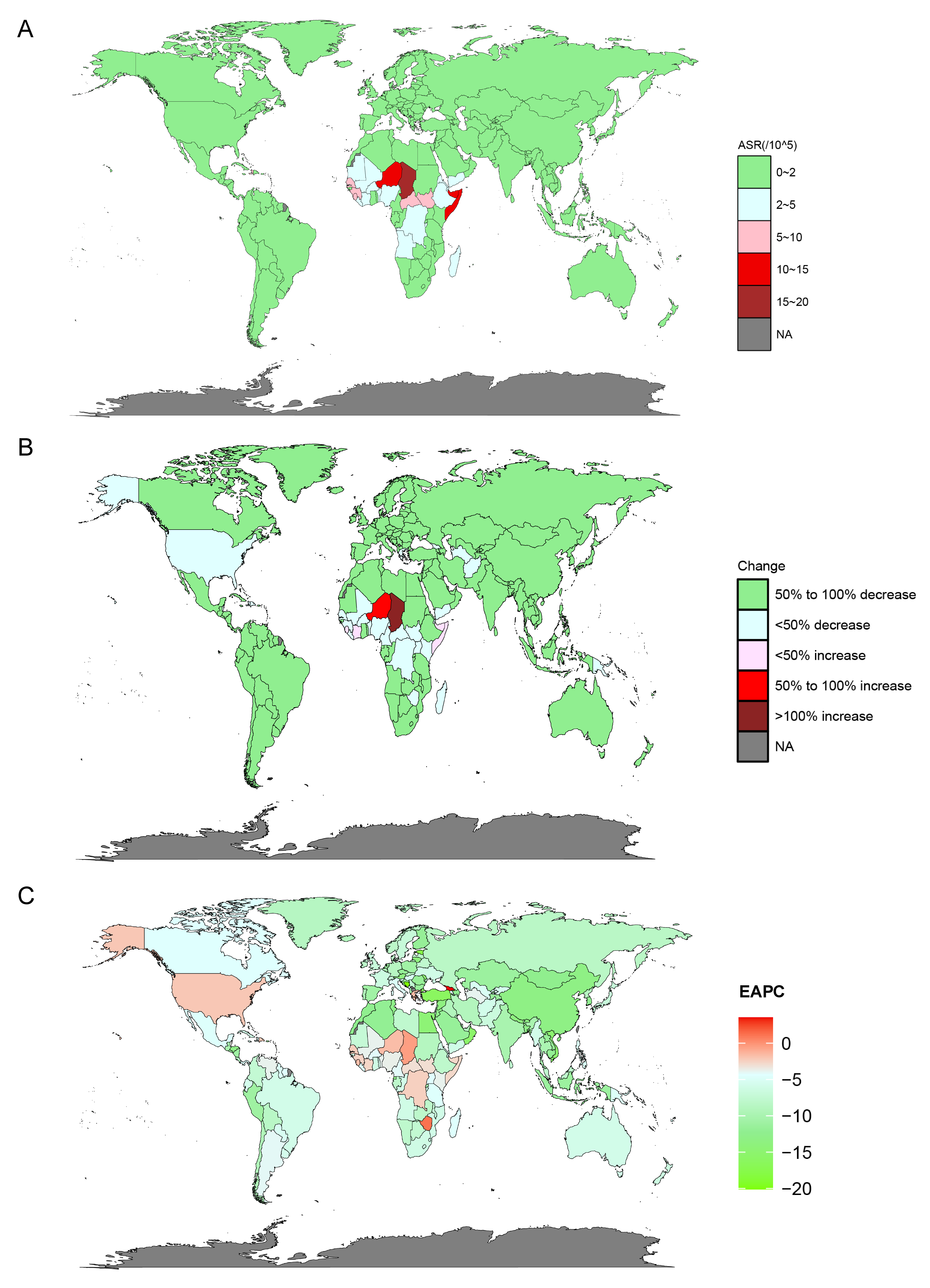

Supplement: Supplementary file 1 — Supplementary Material 1: Supplementary Figure 1. The mortality of abortion and miscarriage in 204 countries and territories. (A) The age-standardized death rate (ASDR) of abortion and miscarriage in 2019. (B) The percent change in deaths cases of abortion and miscarriage from 1990 to 2019. (C) The estimated annual percentage change (EAPC) of abortion and miscarriage in ASDR from 1990 to 2019. [file 12978_2025_2049_MOESM1_ESM.tif]

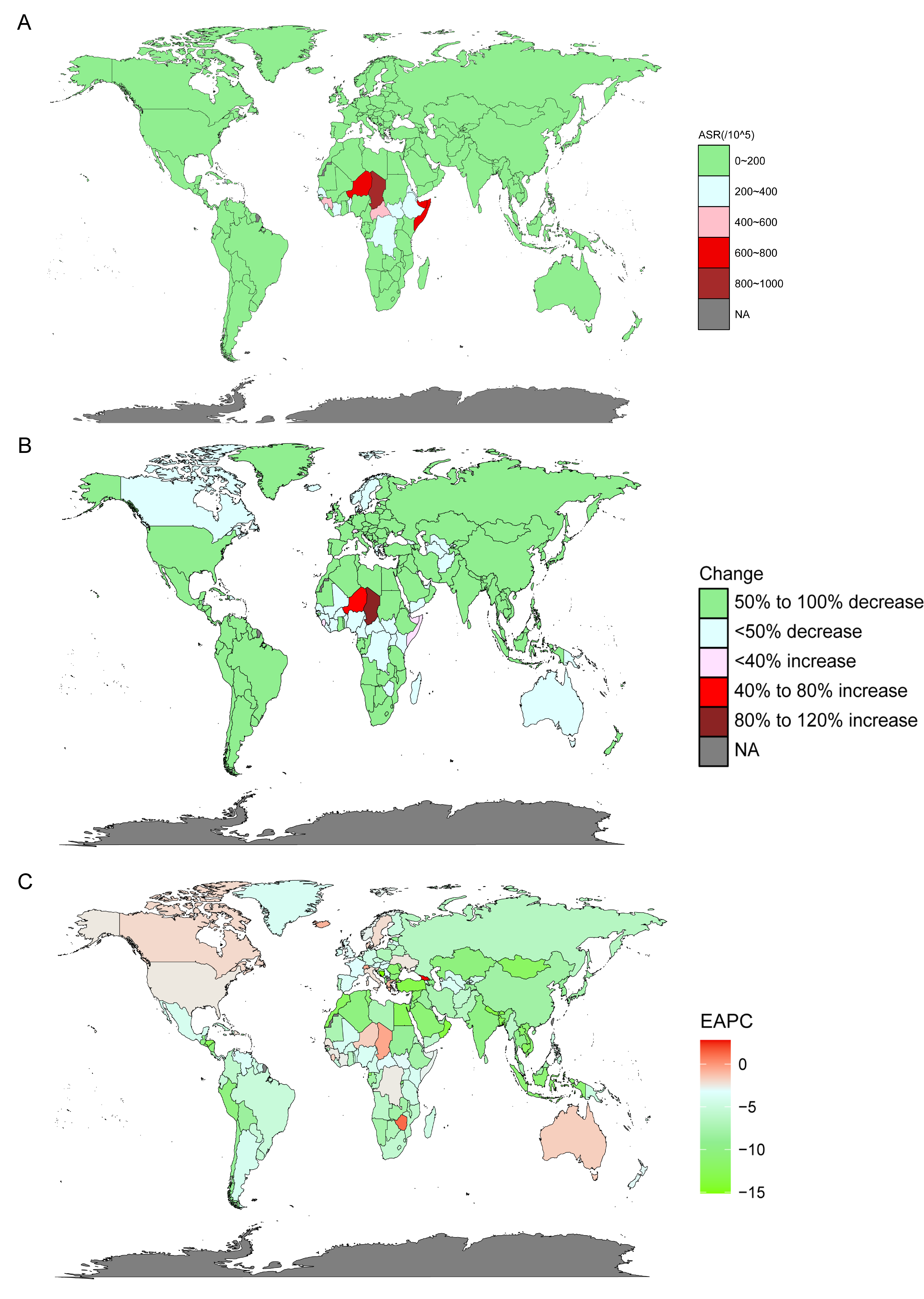

Supplement: Supplementary file 2 — Supplementary Material 2: Supplementary Figure 2. The disability-adjusted life years (DALYs) of abortion and miscarriage in 204 countries and territories. (A) The DALYs rate of abortion and miscarriage in 2019. (B) The percent change in DALYs of abortion and miscarriage from 1990 to 2019. (C) The estimated annual percentage change (EAPC) of abortion and miscarriage in DALYs from 1990 to 2019. [file 12978_2025_2049_MOESM2_ESM.tif]

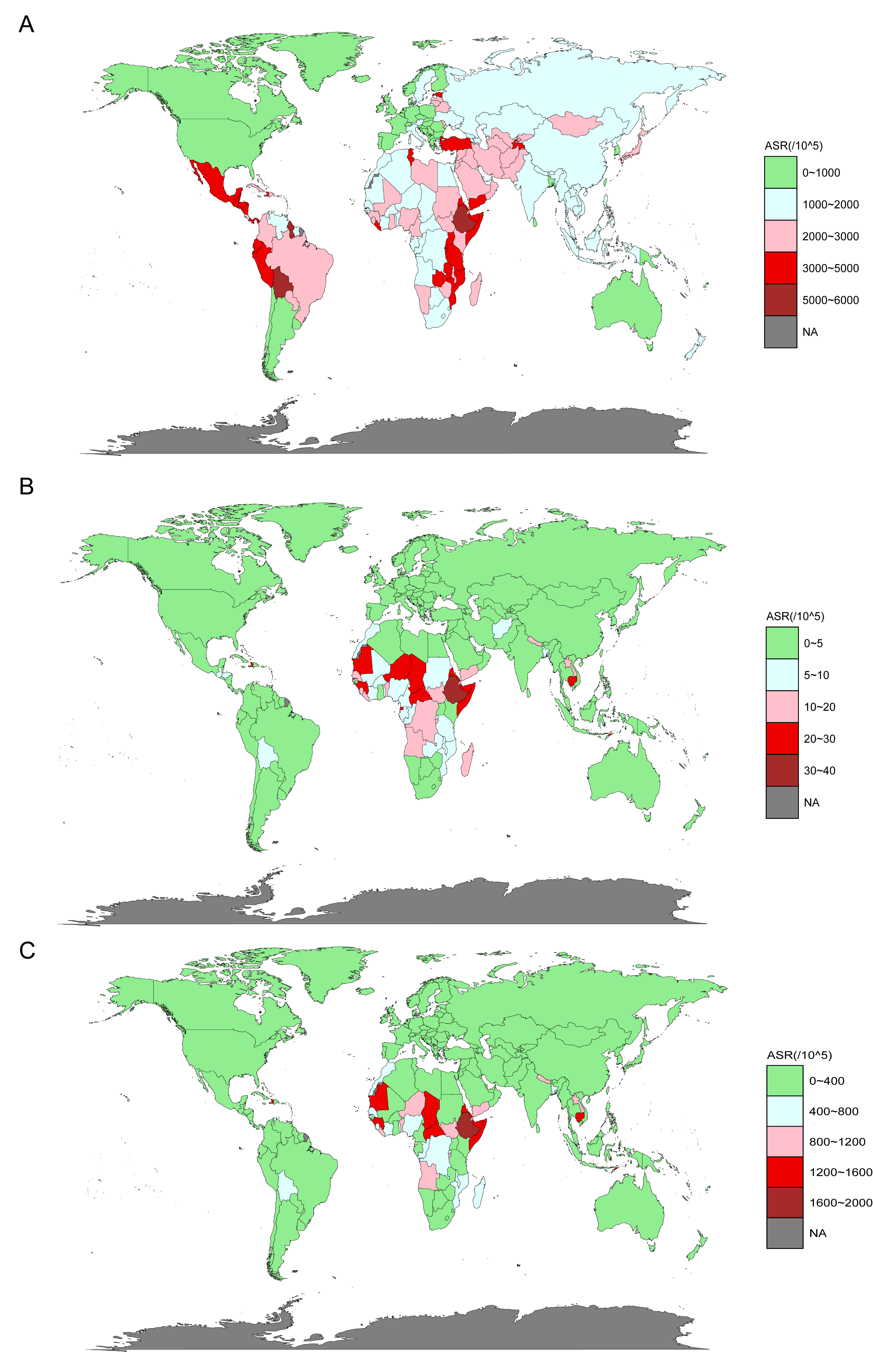

Supplement: Supplementary file 3 — Supplementary Material 3: Supplementary Figure 3. The global burden of abortion and miscarriage in 204 countries and territories in 1990. (A) The age-standardized incidence rate (ASIR) of abortion and miscarriage in 1990. (B) The age-standardized death rate (ASDR) of abortion and miscarriage in 1990. (C) The age-standardized disability-adjusted life years (DALYs) rate of abortion and miscarriage in 1990. [file 12978_2025_2049_MOESM3_ESM.tif]

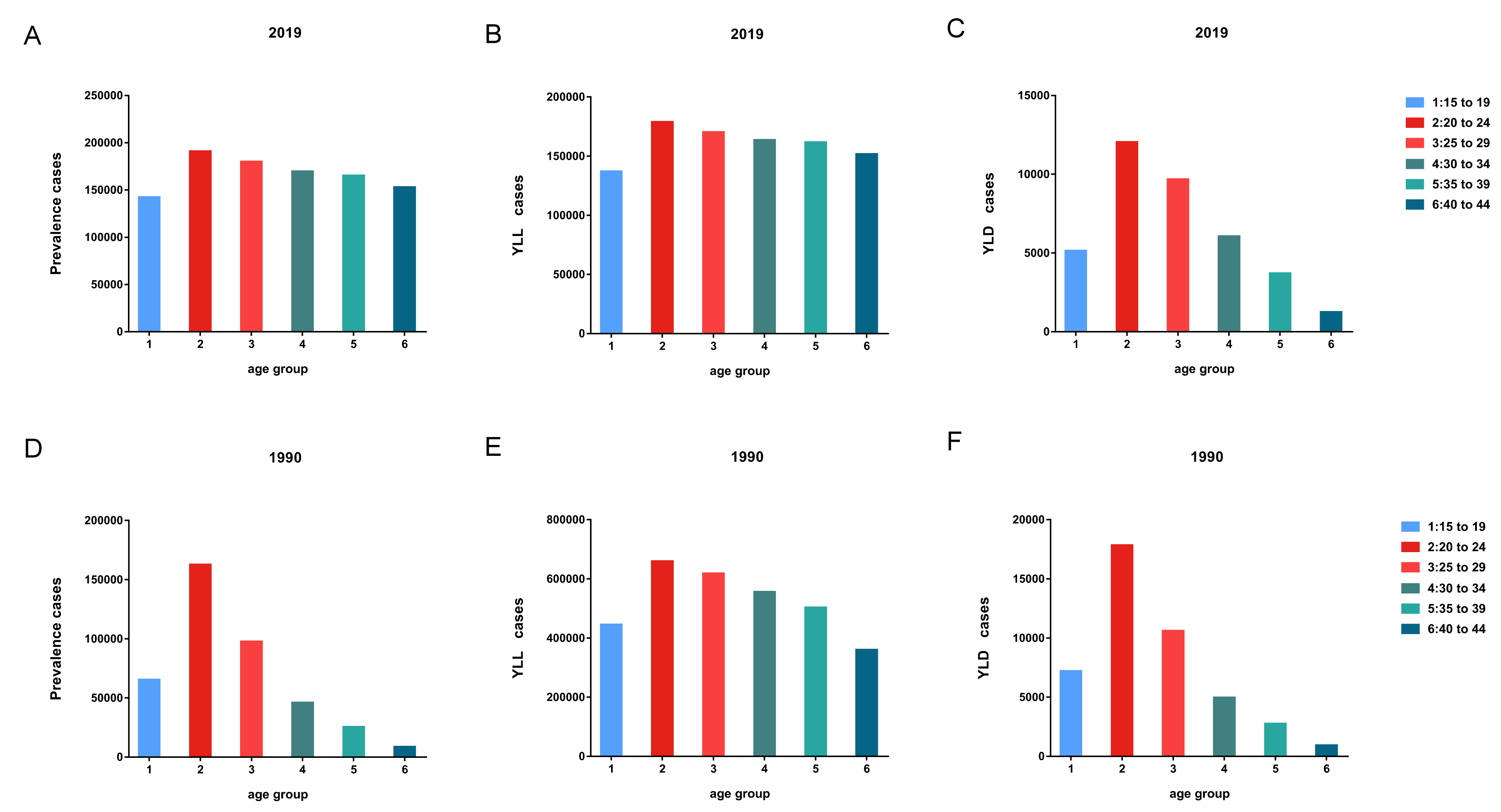

Supplement: Supplementary file 4 — Supplementary Material 4: Supplementary Figure 4. Trends of prevalence of abortion and miscarriage by age groups. The number of prevalence(A), YLL(B), YLD(C) cases of abortion and miscarriage by age groups in 2019. The number of prevalence(D), YLL(E), YLD(F) cases of abortion and miscarriage by age groups in 1990. DALY: Disability-adjusted life year; YLL: year of life lost; YLD: year lived with disability. [file 12978_2025_2049_MOESM4_ESM.tif]

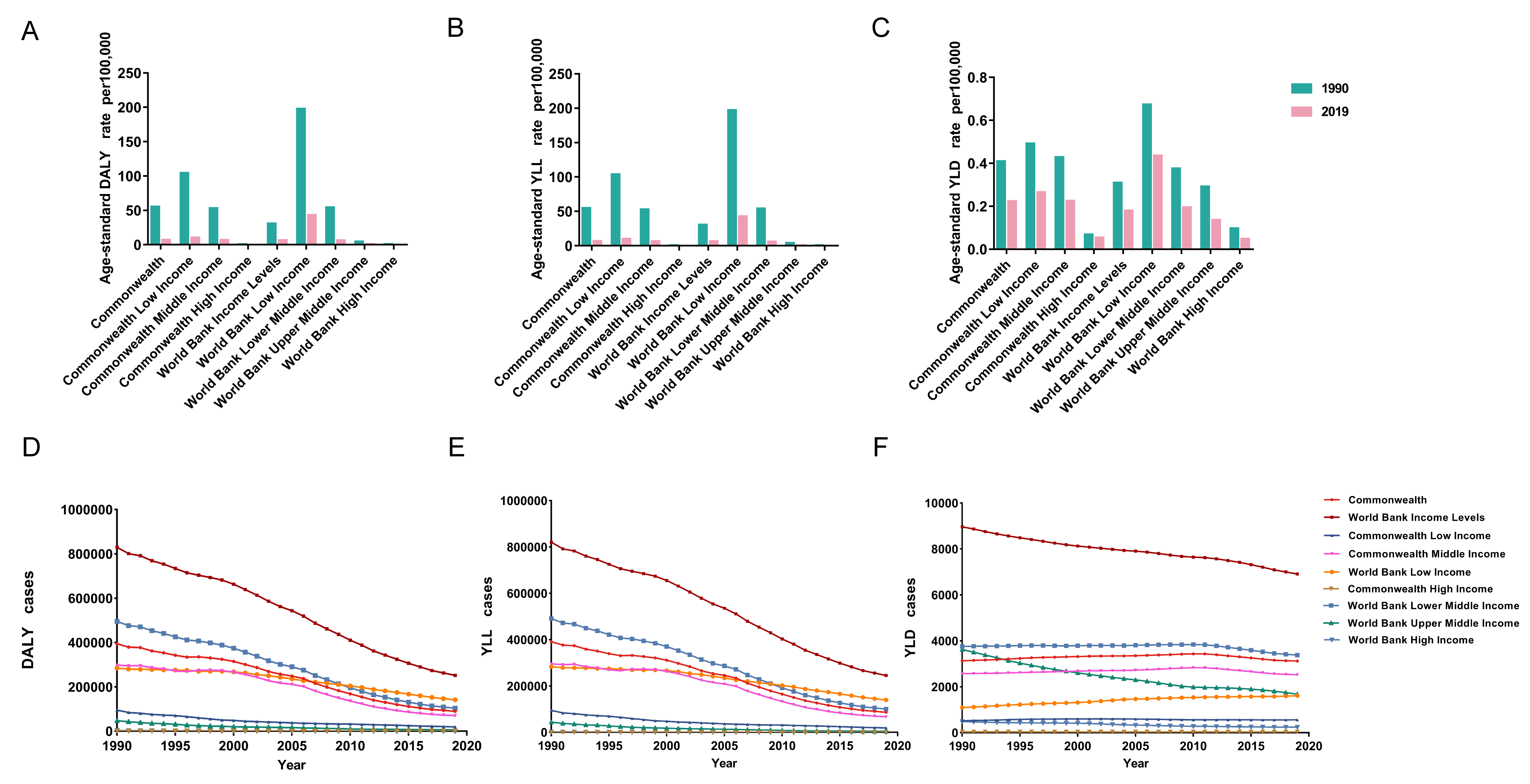

Supplement: Supplementary file 6 — Supplementary Material 6: Supplementary Figure 6. The death burden attributable to risk factor (iron deficiency) of abortion and miscarriage. The age-standardized DALY(A), YLL(B), YLD(C) rate of abortion and miscarriage attributable to iron deficiency in different income areas in 2019. Trends in the number of DALY(D), YLL(E), YLD(F) cases of abortion and miscarriage attributable to iron deficiency in different income areas from 1990 to 2019. DALY: Disability-adjusted life year; YLL: year of life lost; YLD: year lived with disability. [file 12978_2025_2049_MOESM6_ESM.tif]
