# Supplementary material for: Epidemiological trends in abortion and miscarriage between 1990 and 2019
Source: Reprod Health. 2025 Jun 5;22:97. doi: 10.1186/s12978-025-02049-3 (PMC12143105; doi:10.1186/s12978-025-02049-3)
Supplement: Supplementary file 7 — Supplementary Material 7. [file 12978_2025_2049_MOESM7_ESM.docx]

Supplementary appendix to

Epidemiological trend of maternal abortion and miscarriage,1990–2019: results from the Global Burden of Disease Study 2019

[**Contents**](#_Toc119313343)

[Table S1 2](#_Toc119313344)

[Table S2 5](#_Toc119313345)

[Table S3 8](#_Toc119313346)

[Table S4. 27](#_Toc119313347)

[Table S5. 46](#_Toc119313348)

# TableS1. The death cases and age-standardized rates of Maternal abortion and miscarriage in 1990 and 2019, and their temporal changes from 1990 to 2019, for female, in Global, SDI regions and 21 GBD regions

| location | 1990  Number  (95% UI) | 2019  Number  (95% UI) | Change of number  from 1990 to 2019  (95% UI) | 1990  ASDR (per100,000)  (95% UI) | 2019  ASDR (per100,000)  (95% UI) | EAPC in ASDR  (%, 95% CI) |
| --- | --- | --- | --- | --- | --- | --- |
| Global | 59475 (51678-68097) | 19565 (16319-23373) | -0.67 (-0.73--0.61) | 2.25 (1.96-2.59) | 0.5 (0.41-0.59) | -5.21 (-5.38--5.04) |
| Low SDI | 27091 (22545-32366) | 15067 (12306-18431) | -0.44 (-0.56--0.33) | 12.53 (10.4-14.92) | 3.03 (2.47-3.73) | -4.77 (-5.1--4.44) |
| Low-middle SDI | 22462 (19488-25937) | 3432 (2905-4062) | -0.85 (-0.88--0.81) | 4.36 (3.77-5.05) | 0.37 (0.32-0.44) | -8.47 (-8.78--8.16) |
| Middle SDI | 7964 (6878-9132) | 818 (697-958) | -0.9 (-0.92--0.87) | 0.92 (0.8-1.06) | 0.06 (0.05-0.08) | -9.4 (-9.6--9.19) |
| High-middle SDI | 1736 (1595-1896) | 193 (170-216) | -0.89 (-0.9--0.87) | 0.28 (0.26-0.31) | 0.03 (0.02-0.03) | -8.4 (-8.76--8.05) |
| High SDI | 164 (144-189) | 31 (26-37) | -0.81 (-0.85--0.77) | 0.04 (0.03-0.04) | 0.01 (0.01-0.01) | -5.58 (-6.01--5.15) |
| Central Asia | 212 (190-236) | 29 (24-35) | -0.86 (-0.89--0.83) | 0.63 (0.57-0.7) | 0.06 (0.05-0.07) | -9.03 (-9.4--8.66) |
| Central Europe | 202 (185-218) | 6 (5-8) | -0.97 (-0.98--0.96) | 0.33 (0.31-0.36) | 0.01 (0.01-0.02) | -11.91 (-12.49--11.33) |
| Eastern Europe | 193 (154-232) | 18 (12-25) | -0.91 (-0.94--0.86) | 0.17 (0.13-0.2) | 0.02 (0.01-0.02) | -8.36 (-8.62--8.1) |
| Australasia | 1 (1-1) | 0 (0-0) | -0.75 (-0.83--0.63) | 0.01 (0.01-0.01) | 0 (0-0) | -6 (-6.85--5.14) |
| High-income Asia Pacific | 22 (18-26) | 1 (1-2) | -0.94 (-0.95--0.92) | 0.02 (0.02-0.03) | 0 (0-0) | -9.21 (-9.79--8.63) |
| High-income North America | 36 (28-44) | 18 (14-23) | -0.5 (-0.64--0.31) | 0.02 (0.02-0.03) | 0.01 (0.01-0.01) | -2.2 (-2.53--1.88) |
| Southern Latin America | 211 (182-241) | 79 (64-98) | -0.63 (-0.71--0.52) | 0.85 (0.73-0.96) | 0.23 (0.18-0.28) | -4.38 (-4.56--4.2) |
| Western Europe | 59 (53-64) | 7 (6-8) | -0.88 (-0.9--0.86) | 0.03 (0.03-0.03) | 0 (0-0) | -7.45 (-7.81--7.08) |
| Andean Latin America | 634 (533-752) | 95 (65-133) | -0.85 (-0.9--0.78) | 3.57 (2.99-4.29) | 0.28 (0.19-0.4) | -9.1 (-9.61--8.59) |
| Caribbean | 922 (717-1195) | 458 (319-636) | -0.5 (-0.67--0.31) | 5.1 (3.93-6.62) | 1.87 (1.31-2.58) | -2.86 (-3.08--2.63) |
| Central Latin America | 767 (688-851) | 156 (122-197) | -0.8 (-0.84--0.74) | 0.92 (0.83-1.02) | 0.11 (0.09-0.15) | -7.14 (-7.56--6.72) |
| Tropical Latin America | 461 (375-555) | 105 (82-129) | -0.77 (-0.83--0.7) | 0.59 (0.48-0.72) | 0.09 (0.07-0.11) | -5.72 (-6.15--5.29) |
| North Africa and Middle East | 3633 (2963-4382) | 778 (540-1063) | -0.79 (-0.85--0.7) | 2.44 (1.99-2.93) | 0.24 (0.17-0.33) | -7.65 (-7.75--7.55) |
| South Asia | 20192 (16460-24493) | 2119 (1643-2669) | -0.9 (-0.92--0.86) | 4.01 (3.29-4.84) | 0.22 (0.17-0.28) | -9.97 (-10.57--9.36) |
| East Asia | 1233 (999-1500) | 43 (31-58) | -0.97 (-0.98--0.95) | 0.18 (0.15-0.22) | 0.01 (0-0.01) | -11.08 (-11.32--10.84) |
| Oceania | 88 (65-112) | 49 (36-66) | -0.44 (-0.59--0.24) | 3.05 (2.26-3.9) | 0.76 (0.55-1.01) | -4.66 (-4.87--4.46) |
| Southeast Asia | 7351 (6057-8981) | 752 (596-930) | -0.9 (-0.92--0.87) | 3.19 (2.64-3.92) | 0.21 (0.16-0.25) | -9.51 (-9.7--9.31) |
| Central Sub-Saharan Africa | 3028 (2311-3802) | 2179 (1609-2866) | -0.28 (-0.49--0.02) | 13.74 (10.46-17.3) | 3.96 (2.88-5.29) | -3.56 (-3.93--3.18) |
| Eastern Sub-Saharan Africa | 12236 (9720-15457) | 4805 (3802-6020) | -0.61 (-0.71--0.49) | 16.04 (12.79-20.29) | 2.6 (2.07-3.26) | -6.41 (-6.77--6.05) |
| Southern Sub-Saharan Africa | 479 (390-584) | 209 (152-277) | -0.56 (-0.69--0.38) | 1.86 (1.52-2.24) | 0.48 (0.35-0.64) | -2.55 (-3.88--1.2) |
| Western Sub-Saharan Africa | 7515 (5970-9408) | 7658 (5888-9915) | 0.02 (-0.22-0.32) | 10.03 (7.89-12.61) | 3.83 (2.93-5.01) | -3.13 (-3.47--2.78) |

Abbreviations: ASDR: age-standardized death rate; EAPC: estimated annual percentage change; CI: confidence interval; UI: uncertainty interval

# TableS2. The DALY cases and age-standardized rates of Maternal abortion and miscarriage in 1990 and 2019, for female, in Global, SDI regions and 21 GBD regions

| location | 1990  Number  (95% UI) | 2019  Number  (95% UI) | Change of number  from 1990 to 2019  (95% UI) | 1990  ASR (per100,000)  (95% UI) | 2019  ASR (per100,000)  (95% UI) | EAPC in ASR  (%, 95% CI) |
| --- | --- | --- | --- | --- | --- | --- |
| Global | 3449222 (3000198-3952086) | 1130038 (947675-1338397) | -0.67 (-0.73--0.61) | 127.9 (111.35-146.24) | 28.86 (24.21-34.15) | -5.14 (-5.31--4.96) |
| Low SDI | 1538023 (1275796-1830848) | 851228 (695463-1032744) | -0.45 (-0.56--0.33) | 684.62 (571.18-815.38) | 164.56 (134.76-201.11) | -4.8 (-5.13--4.46) |
| Low-middle SDI | 1310487 (1129424-1516780) | 200962 (170110-236408) | -0.85 (-0.88--0.81) | 246.05 (213.82-284.09) | 21.57 (18.24-25.37) | -8.4 (-8.71--8.09) |
| Middle SDI | 474677 (411742-546002) | 56588 (48326-65830) | -0.88 (-0.9--0.86) | 53.29 (46.49-60.83) | 4.52 (3.87-5.26) | -8.73 (-8.93--8.53) |
| High-middle SDI | 109819 (99577-120099) | 16256 (13384-19712) | -0.85 (-0.87--0.82) | 17.82 (16.18-19.48) | 2.37 (1.93-2.9) | -7.18 (-7.58--6.79) |
| High SDI | 12937 (10704-15525) | 3666 (2688-4910) | -0.72 (-0.77--0.66) | 3.03 (2.5-3.63) | 0.81 (0.59-1.09) | -4.4 (-4.61--4.19) |
| Central Asia | 12986 (11603-14435) | 2301 (1846-2868) | -0.82 (-0.86--0.78) | 37.62 (33.78-41.54) | 4.62 (3.7-5.74) | -8.03 (-8.36--7.69) |
| Central Europe | 12045 (11011-13018) | 533 (399-668) | -0.96 (-0.97--0.94) | 20.1 (18.35-21.8) | 1.06 (0.79-1.34) | -10.37 (-11--9.74) |
| Eastern Europe | 13033 (10617-15564) | 2132 (1503-2961) | -0.84 (-0.88--0.77) | 11.58 (9.45-13.78) | 2.22 (1.55-3.08) | -6.08 (-6.33--5.84) |
| Australasia | 145 (91-220) | 99 (51-168) | -0.32 (-0.58-0) | 1.36 (0.85-2.05) | 0.75 (0.38-1.27) | -1.88 (-2.16--1.59) |
| High-income Asia Pacific | 2475 (1841-3246) | 346 (214-511) | -0.86 (-0.89--0.83) | 2.81 (2.08-3.71) | 0.46 (0.28-0.69) | -7.8 (-8.47--7.13) |
| High-income North America | 2995 (2354-3743) | 1500 (1179-1913) | -0.5 (-0.6--0.38) | 2.04 (1.59-2.54) | 0.9 (0.71-1.15) | -2.44 (-2.67--2.22) |
| Southern Latin America | 12456 (10713-14174) | 4632 (3775-5757) | -0.63 (-0.71--0.52) | 49.66 (42.68-56.6) | 13.48 (10.97-16.75) | -4.33 (-4.51--4.14) |
| Western Europe | 4566 (3820-5444) | 1500 (955-2196) | -0.67 (-0.76--0.58) | 2.35 (1.95-2.81) | 0.81 (0.51-1.2) | -3.37 (-3.72--3.03) |
| Andean Latin America | 36692 (31011-43196) | 5933 (4344-7905) | -0.84 (-0.88--0.77) | 199.22 (167.68-236.44) | 17.75 (12.99-23.66) | -8.72 (-9.23--8.2) |
| Caribbean | 53100 (41386-68405) | 25660 (17782-35073) | -0.52 (-0.67--0.33) | 287.22 (223.82-372.51) | 105.36 (72.7-143.63) | -2.85 (-3.08--2.61) |
| Central Latin America | 47964 (43076-52982) | 11190 (8951-13875) | -0.77 (-0.81--0.71) | 55.42 (49.99-61.24) | 8.24 (6.6-10.22) | -6.51 (-6.91--6.12) |
| Tropical Latin America | 27927 (22877-33481) | 7464 (5983-9093) | -0.73 (-0.8--0.66) | 34.87 (28.52-41.71) | 6.27 (5.03-7.63) | -5.07 (-5.45--4.68) |
| North Africa and Middle East | 212351 (174137-256334) | 47740 (34170-64559) | -0.78 (-0.84--0.7) | 137.32 (112.56-165.23) | 14.8 (10.6-19.99) | -7.39 (-7.49--7.3) |
| South Asia | 1187419 (961560-1449500) | 128218 (100218-160745) | -0.89 (-0.92--0.86) | 229.25 (186.83-277) | 13.31 (10.44-16.63) | -9.86 (-10.46--9.26) |
| East Asia | 84114 (68344-101197) | 7165 (4817-10062) | -0.91 (-0.94--0.88) | 11.86 (9.65-14.24) | 1.08 (0.72-1.52) | -8.04 (-8.45--7.64) |
| Oceania | 5001 (3679-6441) | 2805 (2041-3741) | -0.44 (-0.59--0.24) | 167.72 (124.04-214.1) | 42.15 (30.9-55.95) | -4.61 (-4.81--4.4) |
| Southeast Asia | 422270 (347037-516797) | 44505 (35408-54672) | -0.89 (-0.92--0.86) | 177.89 (146.77-217.17) | 12.29 (9.76-15.11) | -9.27 (-9.47--9.08) |
| Central Sub-Saharan Africa | 169341 (129770-211886) | 119606 (89204-155903) | -0.29 (-0.49--0.05) | 736 (564.13-922.67) | 208.42 (153.48-274.68) | -3.62 (-3.99--3.25) |
| Eastern Sub-Saharan Africa | 695290 (554911-876254) | 277098 (219397-344185) | -0.6 (-0.7--0.49) | 868.5 (694-1096.55) | 143.07 (113.83-178.35) | -6.34 (-6.7--5.98) |
| Southern Sub-Saharan Africa | 28086 (23003-34098) | 12539 (9270-16551) | -0.55 (-0.69--0.38) | 105.3 (86.35-127.82) | 28.79 (21.42-37.77) | -2.44 (-3.75--1.11) |
| Western Sub-Saharan Africa | 418964 (333968-523265) | 427074 (328677-547861) | 0.02 (-0.22-0.32) | 531.56 (421.61-665.84) | 204.26 (157.35-264.34) | -3.11 (-3.46--2.76) |

Abbreviations: DALY: disability-adjusted life-year; ASR: age-standardized rate; EAPC: estimated annual percentage change; CI: confidence interval; UI: uncertainty interval

# TableS3. The incidence cases and age-standardized rates of Maternal abortion and miscarriage in 1990 and 2019, and their temporal changes from 1990 to 2019, for female, in 204 countries and territories

| location | 1990  Number  (95% UI) | 2019  Number  (95% UI) | Change of number  from 1990 to 2019  (95% UI) | 1990  ASIR (per100,000)  (95% UI) | 2019  ASIR (per100,000)  (95% UI) | EAPC in ASIR  (%, 95% CI) |
| --- | --- | --- | --- | --- | --- | --- |
| Afghanistan | 161005 (115982-215122) | 422489 (307700-559739) | 1.62 (1.28-2.06) | 2941.5 (2193.57-3882.39) | 2148.75 (1615.93-2804.39) | -1.12 (-1.24--1) |
| Albania | 5027 (3576-6854) | 5773 (4091-7799) | 0.15 (-0.17-0.57) | 276.06 (198.41-371.28) | 454.38 (323.68-616.28) | -0.61 (-1.35-0.12) |
| Algeria | 222346 (168607-286514) | 219981 (165148-297000) | -0.01 (-0.17-0.17) | 1821.11 (1395.62-2320.31) | 934.77 (705.58-1247.33) | -1.91 (-2.17--1.65) |
| American Samoa | 291 (216-385) | 186 (140-239) | -0.36 (-0.45--0.27) | 1046.3 (782.49-1366.13) | 642.03 (481.91-821.18) | -1.68 (-1.78--1.59) |
| Andorra | 85 (63-115) | 82 (61-112) | -0.04 (-0.22-0.22) | 277.69 (207.02-365.66) | 213.11 (158.81-288.23) | -0.98 (-1.14--0.82) |
| Angola | 81180 (60907-105910) | 172299 (129440-222552) | 1.12 (0.88-1.36) | 1633.47 (1233.92-2128.13) | 1140.13 (860.35-1475.45) | -1.2 (-1.33--1.08) |
| Antigua and Barbuda | 532 (387-710) | 363 (266-477) | -0.32 (-0.42--0.22) | 1498.87 (1099.79-1988.02) | 804.07 (590.92-1060.83) | -2.43 (-2.54--2.33) |
| Argentina | 114717 (84477-149784) | 81128 (60527-105078) | -0.29 (-0.4--0.13) | 701.71 (515.22-918.47) | 353.8 (264.08-458.75) | -2.03 (-2.49--1.57) |
| Armenia | 34647 (24219-46386) | 15499 (10891-20710) | -0.55 (-0.62--0.48) | 1869.21 (1306.23-2498.45) | 1121.17 (789.31-1493.97) | -1.45 (-1.92--0.98) |
| Australia | 85301 (61171-114002) | 85059 (61314-113985) | 0 (-0.17-0.21) | 975.02 (697.38-1308.68) | 758.34 (544.08-1015.62) | -0.56 (-0.73--0.39) |
| Austria | 66662 (54771-78337) | 41245 (33561-51508) | -0.38 (-0.48--0.27) | 1662.83 (1372.06-1950.49) | 1076.35 (873.6-1329.62) | -1.52 (-1.62--1.42) |
| Azerbaijan | 84300 (59061-113263) | 64959 (45534-87479) | -0.23 (-0.34--0.11) | 1899.94 (1353.59-2522.96) | 1272.78 (887.92-1713.69) | -0.94 (-1.15--0.73) |
| Bahamas | 2579 (1862-3402) | 2668 (1985-3511) | 0.03 (-0.12-0.22) | 1594.25 (1160.96-2095.75) | 1316.92 (977.93-1733.99) | -0.61 (-0.94--0.29) |
| Bahrain | 8926 (6595-11597) | 7885 (5966-10442) | -0.12 (-0.24-0.04) | 3404.84 (2517.7-4448.33) | 1223.02 (922.01-1607.67) | -3.8 (-3.92--3.69) |
| Bangladesh | 553440 (404921-734291) | 382691 (278511-505028) | -0.31 (-0.4--0.22) | 951.51 (706.04-1251.93) | 403.2 (294.37-529.11) | -2.87 (-2.95--2.8) |
| Barbados | 3386 (2497-4493) | 2265 (1708-2996) | -0.33 (-0.41--0.24) | 2352.49 (1738.2-3107.2) | 1753.57 (1321.52-2302.06) | -0.98 (-1.2--0.75) |
| Belarus | 104856 (83793-131158) | 51607 (37867-69145) | -0.51 (-0.62--0.37) | 2104.22 (1667.91-2632.96) | 1239 (903.63-1654.84) | -4.03 (-5.1--2.95) |
| Belgium | 30581 (21657-41833) | 26352 (18907-36173) | -0.14 (-0.32-0.1) | 632.61 (447.29-861.37) | 547.4 (390.52-748.25) | -0.97 (-1.14--0.8) |
| Belize | 4754 (3433-6185) | 5882 (4229-7768) | 0.24 (0.09-0.4) | 4692.7 (3454.53-6112.01) | 2390.55 (1737.83-3154.51) | -2.27 (-2.33--2.21) |
| Benin | 42716 (32067-55232) | 178729 (133883-237209) | 3.18 (2.53-3.87) | 1785.15 (1357.37-2339.54) | 2735.46 (2060.5-3584.25) | 2.61 (1.93-3.3) |
| Bermuda | 596 (441-803) | 321 (241-438) | -0.46 (-0.56--0.35) | 1784.64 (1326.03-2357.22) | 1201.64 (903.56-1624.38) | -1.53 (-1.81--1.25) |
| Bhutan | 5444 (3938-7308) | 3219 (2329-4417) | -0.41 (-0.48--0.31) | 1725.65 (1272.03-2313.78) | 728.92 (529.43-993.4) | -3.13 (-3.2--3.06) |
| Bolivia (Plurinational State of) | 175035 (128079-226287) | 225040 (166341-289279) | 0.29 (0.14-0.44) | 5250.99 (3909.47-6829.99) | 3524.9 (2622.06-4530.11) | -1.32 (-1.47--1.16) |
| Bosnia and Herzegovina | 10979 (7597-15142) | 4428 (3186-6079) | -0.6 (-0.68--0.5) | 449.4 (311.68-620.49) | 314.43 (224.57-431.46) | -1.3 (-1.54--1.07) |
| Botswana | 8288 (6259-10735) | 15350 (11514-20388) | 0.85 (0.57-1.16) | 1219.13 (937.25-1582.04) | 1092.05 (820.9-1445.45) | -0.54 (-1.02--0.06) |
| Brazil | 1743038 (1275911-2301746) | 1476634 (1078890-1906777) | -0.15 (-0.23--0.07) | 2025.78 (1488.2-2660.44) | 1319.15 (964.49-1705.61) | -1.29 (-1.37--1.21) |
| Brunei Darussalam | 458 (338-600) | 377 (285-494) | -0.18 (-0.29--0.05) | 297.47 (219.83-388.28) | 148.03 (112.05-192.71) | -2.59 (-2.71--2.47) |
| Bulgaria | 17703 (12198-24215) | 10667 (7886-14127) | -0.4 (-0.52--0.22) | 477 (326.84-655.44) | 417.07 (306.58-544.79) | 0.33 (-0.03-0.69) |
| Burkina Faso | 83258 (62288-108314) | 256510 (192802-342923) | 2.08 (1.6-2.62) | 1856.27 (1397.28-2431.44) | 2231.8 (1685.07-2988.11) | 0.71 (0.25-1.17) |
| Burundi | 98151 (72611-130575) | 168702 (124448-219394) | 0.72 (0.54-0.92) | 3667.37 (2750.87-4884.71) | 2915.39 (2175.78-3798.21) | -0.8 (-0.84--0.75) |
| Cabo Verde | 5360 (3935-7059) | 4858 (3590-6450) | -0.09 (-0.22-0.05) | 3044.35 (2279.1-3967.58) | 1543.28 (1143.63-2035.86) | -2.44 (-2.57--2.32) |
| Cambodia | 93062 (69430-122840) | 174001 (124311-233358) | 0.87 (0.49-1.29) | 1663.14 (1244.47-2162.47) | 1865.62 (1340.8-2493.16) | 0.67 (-0.14-1.48) |
| Cameroon | 129505 (96321-172069) | 246940 (183711-332629) | 0.91 (0.68-1.13) | 2491.83 (1871.05-3284.32) | 1547.75 (1165.78-2088.76) | -1.45 (-1.64--1.25) |
| Canada | 36299 (26725-48676) | 72838 (53468-97138) | 1.01 (0.5-1.73) | 249.93 (184.52-334.04) | 456.79 (336.45-608.07) | 0.47 (-0.14-1.09) |
| Central African Republic | 23150 (17324-30507) | 35919 (26720-46746) | 0.55 (0.4-0.75) | 1650.24 (1259.38-2147.96) | 1247.15 (942.37-1636.64) | -0.91 (-0.99--0.83) |
| Chad | 53923 (40329-71576) | 202912 (151733-265975) | 2.76 (2.18-3.44) | 1864.47 (1405.01-2480.75) | 2596.42 (1967.04-3401.41) | 1.75 (1.35-2.16) |
| Chile | 16067 (11770-21740) | 34490 (25588-45077) | 1.15 (0.67-1.76) | 207.34 (153.02-281.12) | 387.35 (286.63-501.09) | 0.78 (-0.36-1.93) |
| China | 11874912 (8448703-15712125) | 5149582 (4090309-6284788) | -0.57 (-0.62--0.49) | 1578.52 (1132.58-2086.28) | 822.82 (648.09-1005.83) | -2.31 (-2.72--1.9) |
| Colombia | 412322 (305812-549515) | 634605 (467668-813282) | 0.54 (0.26-0.82) | 2090.22 (1569.99-2758.79) | 2521.39 (1857.51-3230.29) | 1.46 (0.9-2.03) |
| Comoros | 7624 (5624-10097) | 6072 (4478-7905) | -0.2 (-0.29--0.11) | 3446.42 (2559.45-4566.54) | 1600.2 (1184.67-2086.28) | -2.7 (-2.77--2.64) |
| Congo | 18688 (14209-24551) | 26157 (19584-34201) | 0.4 (0.22-0.6) | 1521.29 (1169.59-2004.24) | 927.52 (694.87-1211.78) | -1.4 (-1.54--1.26) |
| Cook Islands | 95 (71-125) | 49 (37-64) | -0.48 (-0.55--0.41) | 933.69 (694.39-1214.36) | 584.92 (437.85-762.88) | -1.6 (-1.62--1.58) |
| Costa Rica | 43450 (31387-58529) | 33865 (25297-45174) | -0.22 (-0.32--0.1) | 2497.95 (1819.2-3328.35) | 1352.71 (1012.22-1805.33) | -2.12 (-2.25--2) |
| Croatia | 9168 (8084-10483) | 5871 (4469-7908) | -0.36 (-0.51--0.16) | 398.22 (349.28-456.59) | 330.35 (249.22-442.86) | -1 (-1.95--0.05) |
| Cuba | 161177 (114887-209635) | 85946 (63209-109711) | -0.47 (-0.53--0.39) | 2386.44 (1713.14-3095.71) | 1907.85 (1409.87-2423.83) | -0.11 (-0.37-0.15) |
| Cyprus | 3730 (2654-5033) | 2878 (2076-3943) | -0.23 (-0.44-0.05) | 952.98 (678.11-1280.05) | 385.93 (281.4-521.25) | -3.04 (-3.47--2.61) |
| Czechia | 20032 (13970-27312) | 16775 (12016-23727) | -0.16 (-0.4-0.16) | 444.93 (307.02-608.16) | 380.54 (271.97-530.62) | 2.07 (1.26-2.89) |
| Côte d'Ivoire | 96010 (71688-128577) | 146929 (109657-191279) | 0.53 (0.37-0.73) | 1589.11 (1210.3-2104.39) | 1065.71 (796.08-1380.7) | -1.37 (-1.4--1.34) |
| Democratic People's Republic of Korea | 164278 (112098-225655) | 65075 (44766-90101) | -0.6 (-0.67--0.52) | 1351.29 (935.59-1858.62) | 497.97 (342.42-685.39) | -3.45 (-3.48--3.41) |
| Democratic Republic of the Congo | 342540 (256511-444401) | 527699 (393568-688282) | 0.54 (0.38-0.73) | 1881.51 (1417.8-2439.58) | 1213.48 (906.34-1592.81) | -1.41 (-1.61--1.21) |
| Denmark | 21133 (19452-22908) | 21216 (15892-27825) | 0 (-0.24-0.34) | 842.45 (775.83-912.34) | 867.16 (647.46-1139.68) | -0.26 (-0.66-0.14) |
| Djibouti | 5973 (4481-7786) | 9033 (6807-11923) | 0.51 (0.32-0.71) | 2623.98 (1990.74-3446.59) | 1403.5 (1063.68-1849.24) | -2.2 (-2.35--2.05) |
| Dominica | 644 (470-858) | 282 (210-372) | -0.56 (-0.62--0.5) | 1669.03 (1231.74-2186.43) | 856.59 (636.92-1126.76) | -2.55 (-2.63--2.47) |
| Dominican Republic | 112569 (81358-148437) | 52813 (38373-71533) | -0.53 (-0.6--0.45) | 2494.56 (1823.52-3262.66) | 884.96 (644.85-1195.89) | -4.53 (-5.15--3.91) |
| Ecuador | 232157 (186029-280255) | 228945 (195578-270387) | -0.01 (-0.15-0.13) | 4083.12 (3323.27-4920.58) | 2370.17 (2020.12-2789.56) | -1.65 (-1.84--1.46) |
| Egypt | 521880 (380249-698160) | 529531 (386033-691904) | 0.01 (-0.1-0.15) | 1817.51 (1332.79-2424.91) | 995.04 (728.35-1298.55) | -1.76 (-1.88--1.64) |
| El Salvador | 107006 (78084-141778) | 24137 (17515-31985) | -0.77 (-0.81--0.73) | 3458.51 (2547.52-4559.64) | 659.55 (482.76-864.81) | -6.35 (-7.52--5.16) |
| Equatorial Guinea | 4234 (3179-5473) | 8256 (6157-10876) | 0.95 (0.69-1.25) | 2007.14 (1513.83-2592.46) | 1115.13 (829.19-1461.15) | -2.5 (-2.8--2.19) |
| Eritrea | 51148 (37751-67585) | 111408 (82537-146184) | 1.18 (0.91-1.48) | 3534.97 (2653.22-4641.47) | 3150.8 (2379.46-4158.79) | 0.1 (-0.26-0.47) |
| Estonia | 22369 (19485-25679) | 5748 (4412-7266) | -0.74 (-0.81--0.67) | 3174.41 (2751.97-3661.02) | 995.03 (770.76-1257.13) | -4.66 (-5.29--4.02) |
| Eswatini | 5192 (3905-6903) | 10405 (7755-13635) | 1 (0.68-1.33) | 1258.22 (963.53-1676.25) | 1545.24 (1157.74-2009.5) | 1.84 (1.11-2.58) |
| Ethiopia | 1381325 (1037053-1822717) | 2175136 (1625230-2839869) | 0.57 (0.43-0.74) | 5569.06 (4202.62-7310.2) | 3839.06 (2900.13-4987.39) | -1.25 (-1.38--1.12) |
| Fiji | 4342 (3148-5884) | 4985 (3587-6717) | 0.15 (-0.04-0.37) | 998.78 (727.2-1349.94) | 1084.07 (781.38-1463.81) | 0.81 (0.43-1.19) |
| Finland | 11902 (11015-12905) | 10261 (7424-13642) | -0.14 (-0.36-0.14) | 502.03 (462.66-544.88) | 454.59 (330.44-601.43) | -0.53 (-0.77--0.3) |
| France | 247643 (177674-338389) | 208712 (152665-276645) | -0.16 (-0.32-0.07) | 865.5 (619.82-1184.48) | 774.75 (562.43-1030.45) | -0.04 (-0.21-0.14) |
| Gabon | 7405 (5604-9813) | 7664 (5709-10217) | 0.04 (-0.1-0.18) | 1530.19 (1175.17-2008.41) | 751.84 (562.69-1000.79) | -2.48 (-2.5--2.46) |
| Gambia | 12700 (9325-16903) | 17980 (13345-23317) | 0.42 (0.25-0.61) | 2552.7 (1896.13-3334.43) | 1472.59 (1111.79-1927.17) | -1.95 (-2.08--1.82) |
| Georgia | 35375 (25034-47159) | 20232 (16334-24525) | -0.43 (-0.54--0.28) | 1278.97 (902.28-1700.47) | 1407.75 (1132.67-1705.15) | 1.26 (0.74-1.78) |
| Germany | 252553 (177482-340198) | 184214 (132751-246514) | -0.27 (-0.42--0.04) | 652.13 (464.77-872.89) | 543.17 (395.93-724.72) | -0.52 (-0.69--0.35) |
| Ghana | 182311 (137315-240904) | 252338 (186654-332150) | 0.38 (0.22-0.57) | 2422.95 (1838.49-3170.51) | 1378.4 (1032.23-1822.94) | -1.89 (-2--1.78) |
| Greece | 41579 (29297-56327) | 28114 (21304-38171) | -0.32 (-0.49--0.11) | 844.55 (594.4-1144.98) | 665.24 (502.11-890.42) | -0.36 (-0.58--0.13) |
| Greenland | 299 (218-393) | 172 (127-229) | -0.43 (-0.54--0.29) | 959.78 (711.06-1240.87) | 656.26 (488.76-867.94) | -1.51 (-1.65--1.37) |
| Grenada | 1251 (893-1666) | 785 (588-1051) | -0.37 (-0.46--0.25) | 2773.46 (2010.07-3683.05) | 1510.17 (1137.68-2019.86) | -2.09 (-2.28--1.9) |
| Guam | 1521 (1086-2038) | 1097 (779-1481) | -0.28 (-0.37--0.17) | 2015.89 (1447.4-2696.46) | 1420.65 (1015.32-1905.99) | -1.38 (-1.62--1.14) |
| Guatemala | 148275 (109716-194683) | 170233 (126121-226913) | 0.15 (0.01-0.31) | 3658.76 (2750.26-4773.42) | 1562.6 (1163.64-2068.83) | -3.14 (-3.23--3.05) |
| Guinea | 72812 (54183-98309) | 156208 (116555-203877) | 1.15 (0.82-1.53) | 2400.46 (1801.13-3193.06) | 2292.6 (1724.94-2975.18) | -0.08 (-0.45-0.3) |
| Guinea-Bissau | 6189 (4660-8101) | 8160 (6102-10656) | 0.32 (0.17-0.48) | 1218.26 (926.01-1601.63) | 765.64 (577.36-1001.73) | -1.65 (-1.76--1.55) |
| Guyana | 24470 (18897-30563) | 14540 (11219-18356) | -0.41 (-0.48--0.32) | 5046.55 (3933.58-6290.4) | 3215.87 (2501.55-4044.59) | -1.5 (-1.54--1.46) |
| Haiti | 101156 (75173-133546) | 132043 (98462-172122) | 0.31 (0.15-0.49) | 3081.73 (2314.44-4025.33) | 1833.29 (1376.98-2381.07) | -1.82 (-1.84--1.8) |
| Honduras | 112928 (82893-149338) | 93259 (67580-123508) | -0.17 (-0.29--0.04) | 4637.47 (3473.38-6079.31) | 1537.17 (1133.17-2020.47) | -4.22 (-4.76--3.68) |
| Hungary | 18702 (13339-25107) | 16553 (12014-22600) | -0.11 (-0.33-0.19) | 418.04 (294.73-567.03) | 409.09 (295.35-550.87) | -0.14 (-0.39-0.11) |
| Iceland | 781 (745-819) | 932 (688-1218) | 0.19 (-0.11-0.54) | 586.01 (558.65-614.27) | 599.63 (443.41-781.21) | 0.32 (0.2-0.45) |
| India | 7482366 (5465317-9963888) | 7067850 (5089460-9587638) | -0.06 (-0.12-0.02) | 1707.23 (1248.05-2263.62) | 918.93 (662.08-1244.56) | -1.99 (-2.09--1.9) |
| Indonesia | 1485265 (1111947-1949586) | 779548 (624888-953477) | -0.48 (-0.53--0.42) | 1353.2 (1026.32-1751.53) | 568.42 (454.78-696.25) | -3.06 (-3.26--2.85) |
| Iran (Islamic Republic of) | 687369 (506220-894354) | 473530 (354481-622810) | -0.31 (-0.4--0.22) | 2313.98 (1733.04-2956.65) | 1055.25 (783.18-1380.26) | -2.53 (-2.64--2.42) |
| Iraq | 210425 (154014-274887) | 255406 (189369-337446) | 0.21 (0.07-0.39) | 2526.31 (1902.96-3264.39) | 1058.56 (792.54-1381.55) | -3.14 (-3.24--3.04) |
| Ireland | 3994 (3027-5278) | 8449 (6262-11359) | 1.12 (0.56-1.76) | 225.2 (169.97-298.27) | 359.19 (267.08-482.4) | 0.5 (-0.06-1.06) |
| Israel | 26978 (19309-36570) | 45804 (33739-60929) | 0.7 (0.41-1.09) | 1090.95 (779.25-1490.86) | 1076.77 (789.49-1432.27) | -0.11 (-0.22-0) |
| Italy | 322976 (246458-417738) | 199526 (165658-240613) | -0.38 (-0.47--0.28) | 1132.54 (864.12-1465.42) | 885.65 (731.78-1067.63) | -0.63 (-0.76--0.5) |
| Jamaica | 32334 (23114-43245) | 18889 (13784-24990) | -0.42 (-0.49--0.33) | 2293.56 (1660.08-3021.31) | 1190.34 (875.67-1569.93) | -2.33 (-2.41--2.26) |
| Japan | 1275200 (1017515-1583159) | 257124 (205524-329507) | -0.8 (-0.84--0.75) | 2305.72 (1832.79-2875.99) | 557.81 (445.55-705) | -7.63 (-8.67--6.58) |
| Jordan | 44835 (33203-58516) | 90508 (69632-114707) | 1.02 (0.72-1.4) | 2453.87 (1845.86-3181.35) | 1532.86 (1188.67-1947.26) | -1.15 (-1.48--0.82) |
| Kazakhstan | 150765 (105352-202142) | 142130 (101973-188659) | -0.06 (-0.19-0.11) | 1727.45 (1212.78-2321.75) | 1538.59 (1104.09-2032.82) | 0.37 (-0.02-0.75) |
| Kenya | 344464 (255054-457499) | 443215 (328304-593299) | 0.29 (0.22-0.36) | 2975.78 (2249.01-3893.83) | 1534.94 (1149.99-2034.53) | -3.16 (-3.6--2.73) |
| Kiribati | 330 (242-437) | 350 (257-459) | 0.06 (-0.09-0.23) | 783.22 (580.45-1010.76) | 528.49 (389.49-689.55) | -1.34 (-1.39--1.29) |
| Kuwait | 12217 (9119-15973) | 16424 (12206-22109) | 0.34 (0.12-0.59) | 1253.76 (942.29-1644.73) | 590.61 (441.23-782.4) | -3.03 (-3.4--2.66) |
| Kyrgyzstan | 51452 (36528-68708) | 58417 (41895-78812) | 0.14 (-0.03-0.32) | 2193.25 (1575.99-2921.25) | 1644.1 (1189.7-2198.93) | -0.34 (-0.7-0.02) |
| Lao People's Democratic Republic | 38935 (29117-50623) | 45779 (33775-60726) | 0.18 (0.01-0.35) | 1736.57 (1314.1-2259.47) | 1091.77 (811.94-1445.06) | -1.6 (-1.75--1.45) |
| Latvia | 24338 (17878-32378) | 13627 (10166-18210) | -0.44 (-0.56--0.3) | 2000.53 (1469.64-2658.14) | 1693.14 (1264.66-2247.03) | 0.77 (0.31-1.23) |
| Lebanon | 33226 (24771-43461) | 27232 (20294-35581) | -0.18 (-0.28--0.06) | 2036.29 (1524.27-2652.05) | 1069.14 (791.99-1389.95) | -2.18 (-2.21--2.15) |
| Lesotho | 12927 (9717-16766) | 9791 (7364-12878) | -0.24 (-0.33--0.15) | 1465.16 (1110.85-1914.09) | 814.69 (617.2-1069.27) | -2.13 (-2.17--2.1) |
| Liberia | 28528 (21368-37671) | 42734 (31555-56994) | 0.5 (0.31-0.7) | 3083.02 (2299.81-4075.41) | 1625.56 (1205.34-2168.32) | -2.26 (-2.41--2.1) |
| Libya | 43204 (31593-56826) | 20317 (15175-26451) | -0.53 (-0.59--0.46) | 2142.03 (1610.42-2749.76) | 534.45 (396.86-697.19) | -4.7 (-4.9--4.49) |
| Lithuania | 44178 (32141-58946) | 21794 (16375-30121) | -0.51 (-0.61--0.39) | 2441.1 (1770.94-3266.51) | 1855.9 (1394.51-2548.17) | -0.7 (-0.86--0.54) |
| Luxembourg | 1222 (859-1666) | 1479 (1068-2019) | 0.21 (-0.04-0.55) | 626.48 (441.78-846.85) | 479.65 (348-642) | -1.76 (-2.06--1.46) |
| Madagascar | 159888 (117611-210937) | 248780 (185256-330528) | 0.56 (0.4-0.76) | 2704.54 (2018.93-3522.55) | 1711.67 (1278.53-2258.32) | -1.6 (-1.69--1.51) |
| Malawi | 116728 (86475-152997) | 132813 (99777-172589) | 0.14 (0.02-0.28) | 2414.5 (1822.07-3143.75) | 1295.96 (978.32-1679.2) | -2.32 (-2.52--2.13) |
| Malaysia | 101808 (73919-134710) | 95093 (68087-129080) | -0.07 (-0.22-0.12) | 1042.85 (759.41-1375.19) | 547 (394.06-737.61) | -2.66 (-2.89--2.42) |
| Maldives | 2069 (1488-2817) | 1558 (1112-2077) | -0.25 (-0.37--0.1) | 1848.36 (1364.82-2429.05) | 695.19 (494.12-929.54) | -3.25 (-3.54--2.95) |
| Mali | 82453 (62044-106598) | 206222 (155770-272379) | 1.5 (1.14-1.87) | 2010.81 (1529.32-2618.92) | 1897.72 (1437.13-2538.1) | 0 (-0.17-0.17) |
| Malta | 531 (387-718) | 376 (278-507) | -0.29 (-0.41--0.17) | 304.27 (220.41-411.85) | 205.23 (152.69-273.5) | 0.09 (-0.61-0.79) |
| Marshall Islands | 227 (170-298) | 176 (130-226) | -0.23 (-0.31--0.12) | 938.46 (701.64-1227.95) | 572.1 (423.36-735.71) | -1.57 (-1.63--1.5) |
| Mauritania | 15052 (11336-20000) | 17926 (13527-23379) | 0.19 (0.06-0.33) | 1510.01 (1143.97-2004.94) | 860.03 (653.02-1131.21) | -1.84 (-1.95--1.73) |
| Mauritius | 4346 (3155-5769) | 2232 (1666-2911) | -0.49 (-0.56--0.41) | 671.01 (492.31-886.9) | 370.88 (276.51-484.19) | -2.18 (-2.32--2.03) |
| Mexico | 1540794 (1122711-2017647) | 1142507 (883668-1427758) | -0.26 (-0.34--0.18) | 3057.17 (2269.02-3959.85) | 1697.5 (1314.34-2120.85) | -2.08 (-2.12--2.04) |
| Micronesia (Federated States of) | 479 (359-613) | 258 (189-339) | -0.46 (-0.52--0.39) | 921.18 (694.38-1170.56) | 466.49 (342.93-614.23) | -2.3 (-2.34--2.25) |
| Monaco | 76 (54-101) | 62 (45-83) | -0.18 (-0.33-0) | 612.06 (437.65-808.88) | 477.7 (344.77-636.33) | -0.86 (-0.91--0.81) |
| Mongolia | 29156 (21071-39357) | 31788 (23024-41940) | 0.09 (-0.09-0.28) | 2528.28 (1849.84-3380.21) | 1791.58 (1289.12-2360.79) | -0.52 (-1.06-0.01) |
| Montenegro | 1789 (1287-2359) | 1253 (895-1704) | -0.3 (-0.44--0.12) | 563.41 (404.84-740.12) | 444.61 (316.32-599.34) | -0.85 (-0.91--0.8) |
| Morocco | 201380 (150713-263507) | 139600 (105736-178080) | -0.31 (-0.39--0.2) | 1490.58 (1142.08-1910.25) | 723.61 (547.36-921.84) | -2.4 (-2.53--2.27) |
| Mozambique | 251702 (188709-325430) | 456557 (338425-602838) | 0.81 (0.61-1.07) | 3837.45 (2886.11-4941.28) | 2897.47 (2187.08-3753.13) | -0.92 (-1.01--0.82) |
| Myanmar | 294840 (217044-391200) | 209372 (153886-275661) | -0.29 (-0.38--0.18) | 1275.61 (950.06-1677.6) | 698.35 (513.46-918.6) | -2.01 (-2.05--1.97) |
| Namibia | 16815 (12673-21766) | 22175 (16523-29383) | 0.32 (0.18-0.48) | 2455.41 (1874.12-3213.21) | 1648.2 (1242.13-2162.2) | -1.28 (-1.33--1.22) |
| Nauru | 84 (63-109) | 59 (44-75) | -0.3 (-0.41--0.2) | 1563.42 (1169.7-2016.07) | 962.4 (721.52-1244.76) | -1.72 (-1.75--1.69) |
| Nepal | 177168 (129364-234172) | 331811 (264161-410524) | 0.87 (0.52-1.32) | 1754.42 (1293.65-2332.98) | 1712.16 (1370.4-2103.19) | 1.28 (0.32-2.26) |
| Netherlands | 63099 (44923-86186) | 52200 (37226-71294) | -0.17 (-0.33-0) | 787.23 (563.29-1074.9) | 731.67 (519.78-1000.38) | -0.02 (-0.19-0.16) |
| New Zealand | 20902 (15168-27879) | 11671 (9478-14725) | -0.44 (-0.55--0.3) | 1151.35 (835.78-1537.11) | 616.59 (498.74-777.1) | -2.16 (-2.3--2.02) |
| Nicaragua | 67868 (49136-88694) | 29355 (21229-38900) | -0.57 (-0.64--0.48) | 3085.23 (2267.41-4003.92) | 786.77 (573.71-1045.28) | -5.32 (-5.79--4.84) |
| Niger | 75035 (55908-99479) | 348671 (256227-461507) | 3.65 (2.88-4.33) | 1974.27 (1505.01-2621.97) | 3293.98 (2469.62-4304.95) | 2.56 (2.02-3.11) |
| Nigeria | 903922 (679193-1172025) | 1517528 (1155958-1968540) | 0.68 (0.58-0.79) | 2096.25 (1606.97-2721.08) | 1367.96 (1047.73-1791.47) | -1.48 (-1.57--1.39) |
| Niue | 10 (8-13) | 4 (3-5) | -0.58 (-0.64--0.52) | 979.1 (736.97-1286.1) | 580.58 (426.33-760.61) | -1.81 (-1.84--1.79) |
| North Macedonia | 6562 (4645-8843) | 4076 (2884-5526) | -0.38 (-0.52--0.21) | 630.81 (446.47-850.8) | 399.39 (282.47-532.37) | -1.61 (-1.9--1.33) |
| Northern Mariana Islands | 219 (161-286) | 90 (67-118) | -0.59 (-0.64--0.53) | 718.75 (533.16-933.01) | 464.46 (350.25-603.23) | -1.42 (-1.47--1.38) |
| Norway | 15742 (10821-22594) | 17168 (11148-26640) | 0.09 (-0.11-0.33) | 758.09 (520.8-1086.44) | 719.39 (465.08-1112.57) | -1.31 (-1.62--0.99) |
| Oman | 16244 (12166-20986) | 19500 (14394-25732) | 0.2 (0.03-0.4) | 2268.15 (1704.2-2952.56) | 912.3 (678.76-1190.92) | -2.92 (-3.17--2.67) |
| Pakistan | 1131215 (822100-1516066) | 1528836 (1122720-2054751) | 0.35 (0.22-0.5) | 2227.22 (1643.29-2951.54) | 1270.05 (940.2-1689.93) | -1.94 (-2.07--1.8) |
| Palau | 52 (38-68) | 24 (18-31) | -0.53 (-0.59--0.47) | 578.77 (428.82-757.89) | 365.98 (269.05-479.77) | -1.59 (-1.63--1.55) |
| Palestine | 25610 (18820-33984) | 32977 (23824-43943) | 0.29 (0.1-0.46) | 2543.92 (1912.92-3346.39) | 1161.39 (850.41-1535.54) | -2.82 (-3.04--2.61) |
| Panama | 43115 (31067-57097) | 44993 (32756-60446) | 0.04 (-0.09-0.22) | 3076.49 (2248.35-4045.96) | 2141.93 (1562.34-2865.48) | -1.34 (-1.41--1.26) |
| Papua New Guinea | 21243 (15708-27793) | 43012 (31588-56768) | 1.02 (0.8-1.27) | 975.12 (732.02-1257.86) | 807.18 (597.9-1060.24) | -0.71 (-0.76--0.67) |
| Paraguay | 54138 (39496-72534) | 51161 (37544-67132) | -0.05 (-0.16-0.07) | 2618.7 (1931.32-3445.37) | 1329.84 (980.67-1735.41) | -2.28 (-2.33--2.22) |
| Peru | 462010 (337966-609524) | 415033 (303352-553144) | -0.1 (-0.2-0.01) | 3800.78 (2833.63-4949.52) | 2344.41 (1709.3-3135.8) | -1.64 (-1.7--1.58) |
| Philippines | 547959 (406292-723240) | 819808 (617024-1068349) | 0.5 (0.35-0.65) | 1561.47 (1170.78-2030.96) | 1349.67 (1012.71-1750.46) | -0.19 (-0.35--0.03) |
| Poland | 125944 (90958-167808) | 62536 (46012-84566) | -0.5 (-0.61--0.39) | 715.98 (509.33-956.03) | 351.49 (258.26-475.26) | -0.39 (-0.94-0.17) |
| Portugal | 23764 (17005-31781) | 22249 (17781-27497) | -0.06 (-0.26-0.22) | 474.92 (339.22-635.59) | 527.5 (419.91-646.64) | 1.26 (0.73-1.8) |
| Puerto Rico | 38715 (27317-52199) | 14058 (10170-18569) | -0.64 (-0.68--0.59) | 2004.42 (1415.21-2702.32) | 945.2 (681.4-1245.94) | -2.77 (-3.09--2.46) |
| Qatar | 2477 (1816-3225) | 5400 (4007-7216) | 1.18 (0.84-1.63) | 1519.3 (1106.43-1982.52) | 547.08 (412.93-716.11) | -3.62 (-4.11--3.12) |
| Republic of Korea | 94257 (67471-126411) | 42430 (30895-57546) | -0.55 (-0.64--0.43) | 334.64 (239.18-448.9) | 182.97 (133.21-250.89) | -2.26 (-2.54--1.98) |
| Republic of Moldova | 49303 (35777-65411) | 20638 (15433-27058) | -0.58 (-0.65--0.51) | 2234.03 (1610.9-2980.66) | 1212.2 (895.93-1587.07) | -1.69 (-1.98--1.4) |
| Romania | 50735 (35519-69571) | 29739 (21876-39094) | -0.41 (-0.53--0.25) | 450.02 (317.08-616.46) | 405.37 (297.17-533.16) | 2.21 (1.5-2.92) |
| Russian Federation | 1366603 (1004155-1824041) | 894377 (660050-1208990) | -0.35 (-0.46--0.21) | 1928.64 (1392.79-2583.11) | 1371.01 (1006.59-1812.62) | -0.84 (-1.19--0.5) |
| Rwanda | 108394 (80229-143396) | 116355 (86511-154440) | 0.07 (-0.05-0.2) | 3199.64 (2395.98-4231.46) | 1695.91 (1273.06-2246.72) | -2.43 (-2.53--2.33) |
| Saint Kitts and Nevis | 643 (467-850) | 470 (347-615) | -0.27 (-0.37--0.17) | 2764.43 (2029.37-3637.73) | 1600.92 (1184.44-2087.2) | -1.9 (-1.94--1.86) |
| Saint Lucia | 2448 (1752-3290) | 939 (681-1251) | -0.62 (-0.67--0.56) | 3024.38 (2188.16-3999.86) | 1063.52 (772.36-1421.48) | -3.78 (-3.96--3.59) |
| Saint Vincent and the Grenadines | 2392 (1716-3136) | 1252 (941-1646) | -0.48 (-0.55--0.39) | 3747.46 (2732-4878.44) | 2295.99 (1722.97-3014.36) | -1.66 (-1.72--1.59) |
| Samoa | 555 (402-761) | 606 (443-796) | 0.09 (-0.09-0.26) | 671.45 (487-908.02) | 570.08 (416.77-745.35) | -0.19 (-0.4-0.03) |
| San Marino | 38 (27-50) | 40 (30-55) | 0.06 (-0.13-0.3) | 312.71 (229.74-414.68) | 267.13 (198.22-367.97) | -0.54 (-0.59--0.49) |
| Sao Tome and Principe | 1050 (791-1371) | 1025 (766-1362) | -0.02 (-0.15-0.11) | 1913.89 (1469.79-2448.01) | 926.51 (696.2-1226.13) | -2.6 (-2.76--2.44) |
| Saudi Arabia | 145520 (107182-190793) | 104377 (79399-137231) | -0.28 (-0.41--0.14) | 2057.1 (1555.07-2673.08) | 496.34 (378.61-645.77) | -5.12 (-5.24--5) |
| Senegal | 52898 (39297-69655) | 69634 (51803-90348) | 0.32 (0.17-0.48) | 1452.39 (1091.38-1925.78) | 899.88 (674.62-1181.55) | -1.48 (-1.57--1.38) |
| Serbia | 19170 (13803-25607) | 13263 (9507-17941) | -0.31 (-0.46--0.12) | 445.55 (319.14-600.57) | 352.31 (253.4-479.28) | 0.43 (-0.02-0.88) |
| Seychelles | 576 (420-789) | 464 (348-618) | -0.19 (-0.3--0.08) | 1354.49 (996.82-1836.49) | 1052.46 (791.33-1406.45) | -0.58 (-0.77--0.4) |
| Sierra Leone | 42004 (30899-55686) | 70521 (52245-91734) | 0.68 (0.5-0.91) | 2235.29 (1657.48-2913.44) | 1515.11 (1130.19-1963.74) | -1.4 (-1.57--1.22) |
| Singapore | 5558 (4823-6371) | 1964 (1646-2331) | -0.65 (-0.69--0.6) | 269.95 (234.34-309.63) | 60.13 (50.48-71.26) | -5.81 (-6.07--5.55) |
| Slovakia | 19556 (13876-26969) | 13909 (10261-18772) | -0.29 (-0.47--0.07) | 783.94 (550.13-1085.35) | 571.18 (425.95-761.54) | -0.67 (-1--0.33) |
| Slovenia | 4670 (3236-6306) | 4117 (2933-5765) | -0.12 (-0.34-0.16) | 479.15 (331.56-652.77) | 488.4 (345.75-682.46) | 1.12 (0.72-1.52) |
| Solomon Islands | 1864 (1390-2432) | 2473 (1848-3213) | 0.33 (0.17-0.51) | 1047.85 (798.42-1360.66) | 700.33 (523.71-904.94) | -1.43 (-1.49--1.37) |
| Somalia | 97788 (74356-129246) | 248174 (185320-328174) | 1.54 (1.29-1.87) | 3137.98 (2372.92-4172.17) | 2605.54 (1950.78-3432.25) | -0.68 (-0.73--0.63) |
| South Africa | 351645 (263075-458511) | 589771 (446961-788393) | 0.68 (0.47-0.9) | 1674.31 (1266.22-2188.91) | 1856.42 (1406.98-2460.47) | 0.63 (0.16-1.1) |
| South Sudan | 74472 (54376-98562) | 147434 (111222-189538) | 0.98 (0.72-1.28) | 2626.45 (1959.97-3420.32) | 3229.66 (2433.64-4151.85) | 0.58 (0.41-0.76) |
| Spain | 92281 (67123-120945) | 146391 (111110-198227) | 0.59 (0.19-1.1) | 472.54 (344-617.91) | 750.19 (566.75-987.03) | 3.11 (2.44-3.79) |
| Sri Lanka | 69115 (49369-92919) | 47932 (34773-63250) | -0.31 (-0.42--0.17) | 699.06 (502.1-933.18) | 445.88 (321.35-589.18) | -1.29 (-1.42--1.17) |
| Sudan | 310994 (228163-414866) | 456575 (346830-591827) | 0.47 (0.28-0.7) | 2968.45 (2209.22-3907.07) | 2009.74 (1534.11-2607.62) | -1.5 (-1.75--1.26) |
| Suriname | 4176 (3012-5567) | 3765 (2739-4953) | -0.1 (-0.22-0.04) | 1903.23 (1383.73-2506.82) | 1326.59 (964.71-1750.36) | -1.2 (-1.31--1.08) |
| Sweden | 46845 (33724-62352) | 33535 (24137-45761) | -0.28 (-0.41--0.11) | 1214.4 (874.68-1623.52) | 772.23 (558.7-1044.3) | -1.06 (-1.33--0.8) |
| Switzerland | 14045 (9718-18982) | 24556 (18106-33606) | 0.75 (0.29-1.4) | 401.83 (278.18-540.09) | 611.53 (450.21-820.28) | 1.42 (1-1.83) |
| Syrian Arab Republic | 128249 (94764-170639) | 55429 (41548-72271) | -0.57 (-0.62--0.49) | 2042.87 (1521.7-2640.55) | 728.63 (546.4-953.47) | -3.6 (-3.64--3.56) |
| Taiwan (Province of China) | 56022 (39521-75925) | 22796 (16840-30528) | -0.59 (-0.69--0.46) | 470.16 (333.51-635.26) | 208.57 (155-275.12) | -3.51 (-4.03--2.99) |
| Tajikistan | 88182 (63413-116502) | 105362 (74955-140186) | 0.19 (0.03-0.38) | 3186.95 (2305.88-4153.97) | 1979 (1422.29-2619.39) | -1.52 (-1.69--1.36) |
| Thailand | 403066 (290421-543894) | 236977 (170569-319843) | -0.41 (-0.5--0.31) | 1138.74 (824.11-1527.75) | 782.49 (564.24-1058.68) | -0.87 (-1.08--0.66) |
| Timor-Leste | 6079 (4575-7990) | 7491 (5497-9952) | 0.23 (0-0.48) | 1482.88 (1131.76-1910.75) | 1046.35 (771.4-1374.68) | -1 (-1.28--0.72) |
| Togo | 30206 (22406-39530) | 70233 (52301-92469) | 1.33 (0.94-1.69) | 1666.35 (1256.19-2181.47) | 1643.76 (1231.74-2168.68) | 0.76 (0.29-1.23) |
| Tokelau | 13 (10-17) | 7 (5-9) | -0.47 (-0.53--0.38) | 1716.3 (1299.43-2212.52) | 1103.61 (830.6-1411.52) | -1.55 (-1.57--1.52) |
| Tonga | 314 (229-411) | 252 (187-329) | -0.2 (-0.31--0.07) | 657.07 (481.38-853.62) | 496.44 (367.82-649.07) | -0.85 (-0.92--0.78) |
| Trinidad and Tobago | 13626 (9744-18248) | 8258 (5998-11002) | -0.39 (-0.47--0.31) | 2058.3 (1483.48-2749.79) | 1299.63 (935.88-1725) | -1.18 (-1.43--0.92) |
| Tunisia | 156011 (113858-204416) | 90826 (67814-121554) | -0.42 (-0.52--0.29) | 3449.87 (2561.35-4524.29) | 1444.4 (1077.31-1916.76) | -2.86 (-3--2.72) |
| Turkey | 1162676 (832110-1539698) | 573998 (420291-758631) | -0.51 (-0.58--0.43) | 3490.81 (2513.98-4596.43) | 1321.58 (968.99-1754.27) | -3.88 (-4.1--3.65) |
| Turkmenistan | 53582 (37643-72750) | 44987 (31916-60007) | -0.16 (-0.28--0.02) | 2694.36 (1922.81-3623.27) | 1777.87 (1264.32-2368.51) | -1.08 (-1.42--0.74) |
| Tuvalu | 36 (26-46) | 26 (19-34) | -0.27 (-0.35--0.17) | 709.69 (522.36-921.95) | 435.16 (318.02-580.31) | -1.68 (-1.7--1.66) |
| Uganda | 359216 (260518-474399) | 591100 (435763-774735) | 0.65 (0.46-0.86) | 4129.32 (3060.73-5450.17) | 2719.23 (2033.43-3536.96) | -1.54 (-1.67--1.41) |
| Ukraine | 447974 (325915-594156) | 273999 (205683-363498) | -0.39 (-0.49--0.27) | 1900.4 (1372.4-2545.98) | 1455.43 (1077.83-1953.24) | -0.17 (-0.6-0.26) |
| United Arab Emirates | 9398 (6991-12069) | 17191 (13036-23036) | 0.83 (0.43-1.32) | 1259.39 (957.27-1615.24) | 512.12 (390.48-666.45) | -2.47 (-3.06--1.87) |
| United Kingdom | 225666 (164494-296662) | 193096 (145122-254901) | -0.14 (-0.27-0) | 815.41 (599.64-1069.33) | 653.5 (493.11-852.87) | -0.44 (-0.63--0.24) |
| United Republic of Tanzania | 512743 (381896-662569) | 841582 (632952-1091731) | 0.64 (0.44-0.85) | 3890.71 (2922.35-5029.51) | 2765.84 (2092.61-3569.83) | -1.14 (-1.2--1.07) |
| United States of America | 1106161 (958986-1281913) | 503180 (429919-593519) | -0.55 (-0.59--0.49) | 873.8 (758.4-1010.99) | 348.32 (297.17-411.08) | -3.11 (-3.41--2.8) |
| United States Virgin Islands | 2120 (1583-2722) | 1150 (863-1468) | -0.46 (-0.52--0.39) | 3949.87 (2937.97-5077.85) | 2866.57 (2141.54-3667.14) | -1.13 (-1.18--1.09) |
| Uruguay | 8747 (6533-11600) | 12136 (8978-15922) | 0.39 (0.13-0.67) | 580.86 (433.63-770.89) | 761.92 (564.6-998.51) | 0.88 (0.25-1.52) |
| Uzbekistan | 301137 (209641-408316) | 298195 (211896-400283) | -0.01 (-0.15-0.17) | 2646.76 (1884.89-3532.68) | 1589.91 (1134.66-2136.72) | -1.66 (-1.9--1.41) |
| Vanuatu | 1128 (845-1463) | 1458 (1078-1923) | 0.29 (0.11-0.51) | 1408.63 (1055.72-1815.74) | 893.73 (663.66-1167.13) | -1.64 (-1.68--1.6) |
| Venezuela (Bolivarian Republic of) | 205017 (152532-268705) | 177940 (130626-230935) | -0.13 (-0.23--0.01) | 1870.89 (1397.07-2431.02) | 1282.27 (933.94-1670.9) | -0.93 (-1.12--0.74) |
| Viet Nam | 784272 (544552-1086484) | 421648 (331831-538381) | -0.46 (-0.59--0.32) | 1936.48 (1355.64-2647.13) | 852.15 (667.19-1093.63) | -2.86 (-3.02--2.71) |
| Yemen | 196852 (144945-259695) | 266521 (199042-350163) | 0.35 (0.21-0.54) | 3274.36 (2445.28-4309.54) | 1599.09 (1201.7-2093.75) | -2.52 (-2.7--2.33) |
| Zambia | 193474 (141504-253621) | 294902 (219472-388244) | 0.52 (0.35-0.74) | 4765.96 (3576.99-6213.26) | 2901.8 (2185.47-3825.61) | -1.75 (-1.89--1.6) |
| Zimbabwe | 112545 (85334-147235) | 131746 (98177-173075) | 0.17 (0.04-0.33) | 2242.53 (1720.11-2940.38) | 1576.87 (1175.92-2076.18) | -0.86 (-0.99--0.72) |

Abbreviations: ASIR: age-standardized incidence rate; EAPC: estimated annual percentage change; CI: confidence interval; UI: uncertainty interval

# TableS4. The death cases and age-standardized rates of Maternal abortion and miscarriage in 1990 and 2019, and their temporal changes from 1990 to 2019, for female, in 204 countries and territories

| location | 1990  Number  (95% UI) | 2019  Number  (95% UI) | Change of number  from 1990 to 2019  (95% UI) | 1990  ASDR (per100,000)  (95% UI) | 2019  ASDR (per100,000)  (95% UI) | EAPC in ASDR  (%, 95% CI) |
| --- | --- | --- | --- | --- | --- | --- |
| Afghanistan | 256 (177-360) | 154 (103-229) | -0.4 (-0.61--0.06) | 5.68 (3.86-8.11) | 0.99 (0.65-1.47) | -5.84 (-6.56--5.11) |
| Albania | 10 (8-13) | 0 (0-1) | -0.97 (-0.98--0.95) | 0.62 (0.5-0.77) | 0.03 (0.02-0.04) | -9.54 (-10.48--8.58) |
| Algeria | 85 (57-123) | 4 (3-6) | -0.95 (-0.97--0.91) | 0.72 (0.48-1.06) | 0.02 (0.01-0.03) | -11.97 (-12.37--11.56) |
| American Samoa | 0 (0-0) | 0 (0-0) | -0.72 (-0.84--0.53) | 0.68 (0.47-0.93) | 0.15 (0.1-0.22) | -5.67 (-6.4--4.94) |
| Andorra | 0 (0-0) | 0 (0-0) | -0.64 (-0.8--0.36) | 0.01 (0-0.01) | 0 (0-0) | -5.6 (-5.86--5.35) |
| Angola | 808 (542-1091) | 380 (234-562) | -0.53 (-0.73--0.23) | 19.4 (13.1-26.73) | 2.97 (1.83-4.41) | -6.12 (-6.57--5.66) |
| Antigua and Barbuda | 0 (0-0) | 0 (0-0) | -0.67 (-0.78--0.52) | 0.28 (0.21-0.35) | 0.07 (0.05-0.09) | -4.5 (-5.18--3.81) |
| Argentina | 163 (134-194) | 75 (60-94) | -0.54 (-0.66--0.39) | 1.02 (0.84-1.21) | 0.32 (0.25-0.4) | -4.07 (-4.29--3.84) |
| Armenia | 3 (2-4) | 0 (0-1) | -0.84 (-0.9--0.76) | 0.17 (0.13-0.22) | 0.03 (0.02-0.04) | -5.56 (-6.56--4.56) |
| Australia | 1 (1-1) | 0 (0-0) | -0.74 (-0.83--0.6) | 0.01 (0.01-0.01) | 0 (0-0) | -5.92 (-6.88--4.94) |
| Austria | 1 (1-1) | 0 (0-0) | -0.97 (-0.98--0.95) | 0.02 (0.02-0.03) | 0 (0-0) | -13.37 (-14.72--12.01) |
| Azerbaijan | 13 (10-17) | 1 (1-1) | -0.93 (-0.96--0.89) | 0.38 (0.29-0.48) | 0.02 (0.01-0.02) | -12.03 (-12.59--11.47) |
| Bahamas | 1 (1-1) | 0 (0-0) | -0.71 (-0.82--0.56) | 0.53 (0.42-0.66) | 0.11 (0.07-0.16) | -5.29 (-6.02--4.55) |
| Bahrain | 1 (1-1) | 0 (0-0) | -0.72 (-0.84--0.53) | 0.45 (0.33-0.6) | 0.04 (0.03-0.06) | -7.83 (-8.56--7.1) |
| Bangladesh | 4090 (2808-5333) | 163 (110-235) | -0.96 (-0.98--0.93) | 9.27 (6.35-12.18) | 0.19 (0.13-0.27) | -12.75 (-13.3--12.21) |
| Barbados | 0 (0-0) | 0 (0-0) | -0.76 (-0.84--0.63) | 0.23 (0.17-0.28) | 0.06 (0.04-0.08) | -4.73 (-5.42--4.03) |
| Belarus | 6 (5-8) | 1 (0-1) | -0.91 (-0.94--0.86) | 0.11 (0.09-0.14) | 0.01 (0.01-0.02) | -7.75 (-8.27--7.24) |
| Belgium | 2 (1-2) | 0 (0-0) | -0.92 (-0.94--0.88) | 0.03 (0.02-0.04) | 0 (0-0) | -9.63 (-10.84--8.4) |
| Belize | 0 (0-0) | 0 (0-0) | -0.09 (-0.39-0.38) | 0.4 (0.3-0.5) | 0.14 (0.1-0.18) | -3.56 (-3.79--3.33) |
| Benin | 296 (221-386) | 222 (137-347) | -0.25 (-0.54-0.2) | 15.58 (11.45-20.29) | 4.18 (2.61-6.48) | -3.88 (-4.16--3.59) |
| Bermuda | 0 (0-0) | 0 (0-0) | -0.97 (-0.98--0.94) | 0.11 (0.08-0.14) | 0 (0-0.01) | -9.82 (-11.28--8.35) |
| Bhutan | 9 (5-14) | 0 (0-0) | -0.97 (-0.98--0.94) | 3.58 (1.96-5.33) | 0.07 (0.04-0.12) | -13.32 (-13.57--13.06) |
| Bolivia (Plurinational State of) | 273 (206-352) | 53 (32-82) | -0.8 (-0.89--0.69) | 9.51 (7.15-12.3) | 0.9 (0.55-1.38) | -8.42 (-8.8--8.03) |
| Bosnia and Herzegovina | 6 (5-7) | 0 (0-0) | -1 (-1--0.99) | 0.25 (0.2-0.31) | 0 (0-0) | -20.13 (-21.76--18.47) |
| Botswana | 5 (3-8) | 1 (1-2) | -0.76 (-0.88--0.58) | 0.91 (0.57-1.35) | 0.09 (0.05-0.16) | -7.54 (-8.29--6.79) |
| Brazil | 424 (332-519) | 88 (66-112) | -0.79 (-0.85--0.71) | 0.56 (0.44-0.68) | 0.07 (0.06-0.1) | -5.89 (-6.46--5.32) |
| Brunei Darussalam | 0 (0-0) | 0 (0-0) | -0.8 (-0.87--0.67) | 0.17 (0.12-0.24) | 0.02 (0.01-0.03) | -7.12 (-8--6.23) |
| Bulgaria | 12 (10-14) | 1 (0-1) | -0.95 (-0.97--0.93) | 0.28 (0.23-0.33) | 0.02 (0.01-0.02) | -11.46 (-12.53--10.37) |
| Burkina Faso | 247 (165-344) | 215 (136-313) | -0.13 (-0.46-0.37) | 6.23 (4.18-8.7) | 2.07 (1.31-3.05) | -4.2 (-4.51--3.89) |
| Burundi | 439 (299-610) | 201 (138-277) | -0.54 (-0.71--0.27) | 18.83 (12.86-26.28) | 4.01 (2.68-5.68) | -6.17 (-6.65--5.69) |
| Cabo Verde | 4 (3-6) | 0 (0-1) | -0.91 (-0.95--0.87) | 2.68 (2.04-3.45) | 0.12 (0.08-0.18) | -10.07 (-10.84--9.29) |
| Cambodia | 1114 (833-1460) | 84 (56-121) | -0.93 (-0.95--0.89) | 24.34 (18.18-32) | 0.99 (0.65-1.43) | -11.88 (-12.49--11.27) |
| Cameroon | 268 (195-354) | 226 (131-344) | -0.15 (-0.5-0.31) | 6.28 (4.54-8.49) | 1.69 (0.99-2.61) | -4.16 (-4.84--3.48) |
| Canada | 2 (2-3) | 1 (0-1) | -0.71 (-0.8--0.57) | 0.01 (0.01-0.02) | 0 (0-0) | -4.42 (-5.07--3.78) |
| Central African Republic | 294 (221-377) | 236 (156-355) | -0.2 (-0.47-0.21) | 25.15 (18.75-32.49) | 9.84 (6.34-14.77) | -3.07 (-3.43--2.7) |
| Chad | 543 (399-692) | 1137 (778-1519) | 1.09 (0.37-2.01) | 22.16 (16.28-28.61) | 17.59 (12.09-23.84) | -0.48 (-1.04-0.09) |
| Chile | 42 (34-51) | 2 (1-3) | -0.95 (-0.97--0.93) | 0.56 (0.46-0.68) | 0.02 (0.02-0.03) | -9.25 (-10.27--8.21) |
| China | 977 (770-1223) | 16 (12-21) | -0.98 (-0.99--0.98) | 0.15 (0.11-0.18) | 0 (0-0) | -13.16 (-13.4--12.92) |
| Colombia | 105 (81-134) | 17 (11-24) | -0.84 (-0.91--0.75) | 0.59 (0.46-0.75) | 0.07 (0.04-0.1) | -7.44 (-8.13--6.75) |
| Comoros | 16 (3-26) | 2 (1-3) | -0.88 (-0.94--0.41) | 8.63 (2.08-13.98) | 0.55 (0.28-0.85) | -10.22 (-10.91--9.54) |
| Congo | 85 (60-116) | 38 (24-58) | -0.55 (-0.73--0.26) | 8.6 (6-11.8) | 1.45 (0.91-2.22) | -5.88 (-6.61--5.15) |
| Cook Islands | 0 (0-0) | 0 (0-0) | -0.81 (-0.91--0.62) | 0.04 (0.02-0.06) | 0.01 (0-0.01) | -5.66 (-6.18--5.14) |
| Costa Rica | 6 (5-7) | 1 (1-2) | -0.77 (-0.85--0.65) | 0.37 (0.3-0.44) | 0.05 (0.03-0.07) | -8.37 (-9.32--7.41) |
| Croatia | 1 (1-1) | 0 (0-0) | -0.97 (-0.98--0.95) | 0.03 (0.02-0.04) | 0 (0-0) | -11.14 (-12.16--10.12) |
| Cuba | 24 (19-30) | 4 (3-5) | -0.85 (-0.9--0.78) | 0.39 (0.31-0.48) | 0.07 (0.05-0.1) | -5.81 (-6.2--5.42) |
| Cyprus | 0 (0-0) | 0 (0-0) | -0.94 (-0.96--0.9) | 0.02 (0.01-0.02) | 0 (0-0) | -13.06 (-13.56--12.57) |
| Czechia | 3 (2-3) | 0 (0-0) | -0.97 (-0.98--0.96) | 0.05 (0.04-0.06) | 0 (0-0) | -11.71 (-12.79--10.61) |
| Côte d'Ivoire | 441 (309-592) | 443 (274-654) | 0 (-0.42-0.59) | 9.35 (6.45-12.61) | 3.82 (2.34-5.61) | -2.67 (-3.24--2.1) |
| Democratic People's Republic of Korea | 252 (155-380) | 25 (15-39) | -0.9 (-0.95--0.82) | 2.28 (1.41-3.49) | 0.19 (0.11-0.29) | -8.59 (-9.27--7.9) |
| Democratic Republic of the Congo | 1780 (1259-2424) | 1516 (1070-2068) | -0.15 (-0.45-0.3) | 11.78 (8.37-16.25) | 4.25 (2.93-5.99) | -2.46 (-2.9--2.01) |
| Denmark | 0 (0-0) | 0 (0-0) | -0.78 (-0.85--0.67) | 0.01 (0-0.01) | 0 (0-0) | -8.23 (-9.69--6.75) |
| Djibouti | 33 (21-47) | 22 (11-35) | -0.32 (-0.64-0.22) | 15.99 (10.15-22.59) | 3.55 (1.77-5.57) | -5.29 (-5.8--4.77) |
| Dominica | 0 (0-0) | 0 (0-0) | -0.63 (-0.78--0.4) | 1.03 (0.78-1.36) | 0.4 (0.26-0.58) | -3.38 (-3.62--3.15) |
| Dominican Republic | 33 (25-43) | 20 (13-29) | -0.41 (-0.64--0.05) | 0.87 (0.65-1.11) | 0.34 (0.22-0.5) | -2.19 (-3.02--1.35) |
| Ecuador | 48 (37-61) | 14 (10-22) | -0.7 (-0.82--0.52) | 0.96 (0.74-1.23) | 0.15 (0.1-0.23) | -5.67 (-6.51--4.83) |
| Egypt | 236 (181-298) | 8 (5-12) | -0.97 (-0.98--0.95) | 0.92 (0.71-1.16) | 0.02 (0.01-0.02) | -14.48 (-15.13--13.83) |
| El Salvador | 94 (74-113) | 4 (3-7) | -0.95 (-0.97--0.93) | 3.57 (2.84-4.34) | 0.12 (0.08-0.19) | -10.87 (-11.97--9.76) |
| Equatorial Guinea | 37 (25-51) | 3 (2-6) | -0.91 (-0.96--0.83) | 20.58 (13.94-28.78) | 0.56 (0.28-0.99) | -12.87 (-13.3--12.43) |
| Eritrea | 312 (202-435) | 125 (81-197) | -0.6 (-0.75--0.33) | 23.8 (15.31-33.56) | 3.81 (2.5-5.9) | -6.14 (-6.53--5.74) |
| Estonia | 1 (1-1) | 0 (0-0) | -0.99 (-1--0.99) | 0.1 (0.08-0.13) | 0 (0-0) | -17.88 (-19.79--15.93) |
| Eswatini | 8 (5-11) | 2 (1-3) | -0.75 (-0.88--0.52) | 2.35 (1.55-3.41) | 0.34 (0.16-0.61) | -5.69 (-6.94--4.42) |
| Ethiopia | 7040 (5296-9614) | 1717 (1198-2362) | -0.76 (-0.86--0.62) | 35.04 (26.28-47.66) | 3.71 (2.59-5.18) | -7.96 (-8.56--7.35) |
| Fiji | 4 (3-6) | 2 (1-2) | -0.63 (-0.79--0.36) | 1.23 (0.86-1.73) | 0.36 (0.23-0.52) | -4.57 (-4.74--4.41) |
| Finland | 1 (1-1) | 0 (0-0) | -0.97 (-0.98--0.95) | 0.02 (0.02-0.03) | 0 (0-0) | -11.72 (-12.74--10.69) |
| France | 10 (7-12) | 2 (1-2) | -0.83 (-0.89--0.76) | 0.03 (0.02-0.04) | 0.01 (0-0.01) | -6.48 (-7.22--5.73) |
| Gabon | 23 (17-31) | 5 (3-8) | -0.77 (-0.87--0.63) | 6.03 (4.28-8.04) | 0.58 (0.34-0.9) | -8.04 (-8.67--7.41) |
| Gambia | 41 (27-58) | 36 (24-51) | -0.12 (-0.47-0.47) | 11.04 (7.13-16.12) | 3.79 (2.48-5.53) | -3.59 (-4.2--2.98) |
| Georgia | 2 (2-3) | 1 (1-2) | -0.4 (-0.61--0.05) | 0.07 (0.05-0.1) | 0.07 (0.05-0.1) | 3.54 (2.06-5.05) |
| Germany | 17 (13-21) | 1 (1-1) | -0.94 (-0.96--0.9) | 0.04 (0.03-0.05) | 0 (0-0) | -8.7 (-9.68--7.71) |
| Ghana | 206 (137-288) | 67 (42-95) | -0.67 (-0.82--0.48) | 2.94 (2-4.06) | 0.39 (0.25-0.55) | -6.24 (-6.46--6.02) |
| Greece | 1 (1-1) | 1 (0-1) | -0.4 (-0.58--0.15) | 0.02 (0.02-0.02) | 0.01 (0.01-0.01) | -1.61 (-2.77--0.43) |
| Greenland | 0 (0-0) | 0 (0-0) | -0.91 (-0.95--0.84) | 0.02 (0.02-0.04) | 0 (0-0) | -8.71 (-9.14--8.28) |
| Grenada | 0 (0-0) | 0 (0-0) | -0.83 (-0.89--0.74) | 0.53 (0.41-0.68) | 0.07 (0.05-0.09) | -6.24 (-6.94--5.52) |
| Guam | 0 (0-0) | 0 (0-0) | -0.14 (-0.5-0.41) | 0.11 (0.08-0.15) | 0.08 (0.05-0.11) | -1.58 (-2.31--0.85) |
| Guatemala | 185 (143-237) | 38 (26-53) | -0.79 (-0.86--0.7) | 5.26 (4.06-6.76) | 0.38 (0.26-0.54) | -10.09 (-11.08--9.09) |
| Guinea | 614 (468-786) | 557 (389-767) | -0.09 (-0.38-0.29) | 23.53 (17.93-30.07) | 9.95 (6.84-14.09) | -2.58 (-2.9--2.26) |
| Guinea-Bissau | 21 (14-29) | 7 (4-10) | -0.69 (-0.81--0.49) | 4.64 (3.1-6.67) | 0.69 (0.43-1.04) | -6.34 (-6.97--5.71) |
| Guyana | 8 (6-10) | 2 (1-3) | -0.76 (-0.86--0.61) | 1.82 (1.34-2.42) | 0.42 (0.27-0.63) | -4.92 (-5.23--4.61) |
| Haiti | 806 (608-1073) | 413 (278-582) | -0.49 (-0.67--0.26) | 27.56 (20.66-36.74) | 6.13 (4.12-8.75) | -4.59 (-4.87--4.3) |
| Honduras | 127 (95-167) | 8 (4-12) | -0.94 (-0.97--0.9) | 6.05 (4.49-7.95) | 0.14 (0.08-0.23) | -12.99 (-13.53--12.46) |
| Hungary | 2 (2-3) | 0 (0-0) | -0.89 (-0.93--0.82) | 0.05 (0.04-0.06) | 0.01 (0-0.01) | -5.06 (-6.57--3.52) |
| Iceland | 0 (0-0) | 0 (0-0) | -0.91 (-0.94--0.86) | 0.01 (0-0.01) | 0 (0-0) | -8.92 (-9.36--8.48) |
| India | 13437 (10116-17204) | 1436 (997-1959) | -0.89 (-0.93--0.84) | 3.34 (2.53-4.23) | 0.2 (0.14-0.27) | -10.03 (-10.82--9.23) |
| Indonesia | 4009 (2993-5175) | 239 (166-338) | -0.94 (-0.96--0.91) | 4.43 (3.33-5.7) | 0.17 (0.12-0.24) | -11.17 (-11.34--10.99) |
| Iran (Islamic Republic of) | 90 (69-115) | 9 (7-12) | -0.9 (-0.93--0.85) | 0.36 (0.27-0.46) | 0.02 (0.02-0.03) | -9.05 (-9.5--8.59) |
| Iraq | 36 (24-52) | 6 (4-10) | -0.82 (-0.9--0.69) | 0.52 (0.34-0.77) | 0.03 (0.02-0.05) | -9.12 (-9.61--8.62) |
| Ireland | 0 (0-0) | 0 (0-0) | -0.86 (-0.91--0.79) | 0.02 (0.01-0.03) | 0 (0-0) | -8.03 (-9.45--6.6) |
| Israel | 2 (2-3) | 0 (0-0) | -0.83 (-0.88--0.76) | 0.08 (0.07-0.11) | 0.01 (0.01-0.01) | -8.14 (-9.03--7.24) |
| Italy | 7 (6-9) | 1 (1-2) | -0.82 (-0.87--0.75) | 0.02 (0.02-0.03) | 0 (0-0.01) | -4.95 (-5.49--4.41) |
| Jamaica | 5 (4-6) | 2 (1-3) | -0.56 (-0.73--0.3) | 0.39 (0.3-0.5) | 0.13 (0.08-0.19) | -5.32 (-6.32--4.31) |
| Japan | 8 (6-11) | 1 (1-1) | -0.89 (-0.92--0.84) | 0.01 (0.01-0.02) | 0 (0-0) | -6.89 (-7.21--6.56) |
| Jordan | 10 (7-14) | 1 (1-2) | -0.87 (-0.92--0.77) | 0.81 (0.55-1.13) | 0.03 (0.02-0.04) | -12.66 (-13.42--11.9) |
| Kazakhstan | 104 (87-121) | 7 (5-9) | -0.94 (-0.96--0.91) | 1.21 (1.02-1.39) | 0.07 (0.05-0.09) | -11.18 (-12.21--10.14) |
| Kenya | 393 (284-515) | 314 (210-445) | -0.2 (-0.48-0.19) | 4.3 (3.09-5.64) | 1.3 (0.86-1.85) | -3.82 (-4.16--3.47) |
| Kiribati | 2 (2-3) | 1 (1-2) | -0.57 (-0.74--0.29) | 6.95 (4.79-9.67) | 1.76 (1.16-2.54) | -4.83 (-4.97--4.68) |
| Kuwait | 0 (0-0) | 0 (0-0) | -0.6 (-0.76--0.38) | 0.04 (0.03-0.05) | 0 (0-0.01) | -8.76 (-10.4--7.1) |
| Kyrgyzstan | 6 (4-7) | 1 (1-2) | -0.76 (-0.84--0.64) | 0.27 (0.2-0.34) | 0.04 (0.03-0.05) | -5.57 (-6--5.14) |
| Lao People's Democratic Republic | 284 (202-398) | 17 (11-26) | -0.94 (-0.96--0.9) | 15.01 (10.71-21.24) | 0.46 (0.28-0.66) | -11.67 (-12.43--10.9) |
| Latvia | 1 (1-2) | 0 (0-0) | -0.92 (-0.96--0.88) | 0.09 (0.07-0.12) | 0.01 (0.01-0.02) | -8.09 (-9.8--6.36) |
| Lebanon | 3 (2-4) | 0 (0-1) | -0.85 (-0.91--0.73) | 0.2 (0.13-0.29) | 0.02 (0.01-0.02) | -8.07 (-8.68--7.46) |
| Lesotho | 26 (18-37) | 18 (9-29) | -0.33 (-0.65-0.22) | 3.47 (2.27-4.98) | 1.68 (0.89-2.76) | -0.41 (-1.54-0.72) |
| Liberia | 127 (90-172) | 83 (52-118) | -0.35 (-0.62-0.03) | 16.43 (11.52-22.03) | 3.69 (2.3-5.35) | -4.7 (-5.16--4.23) |
| Libya | 4 (2-5) | 1 (1-1) | -0.76 (-0.87--0.56) | 0.27 (0.17-0.39) | 0.02 (0.01-0.03) | -7.85 (-8.52--7.17) |
| Lithuania | 3 (2-4) | 0 (0-0) | -0.99 (-0.99--0.98) | 0.16 (0.12-0.2) | 0 (0-0) | -13.69 (-15.35--11.99) |
| Luxembourg | 0 (0-0) | 0 (0-0) | -0.82 (-0.88--0.73) | 0.03 (0.02-0.04) | 0 (0-0) | -7.66 (-8.08--7.23) |
| Madagascar | 547 (418-696) | 363 (256-499) | -0.34 (-0.56--0.01) | 10.38 (7.93-13.28) | 2.89 (1.98-4.01) | -4.5 (-4.85--4.16) |
| Malawi | 379 (279-507) | 102 (61-155) | -0.73 (-0.85--0.54) | 9.23 (6.76-12.22) | 1.17 (0.7-1.79) | -6.98 (-7.49--6.47) |
| Malaysia | 44 (32-59) | 9 (6-13) | -0.8 (-0.89--0.68) | 0.54 (0.38-0.73) | 0.05 (0.03-0.08) | -7.85 (-8.48--7.23) |
| Maldives | 4 (3-6) | 0 (0-0) | -0.96 (-0.98--0.94) | 5.35 (3.59-7.81) | 0.07 (0.05-0.1) | -14.49 (-15.14--13.83) |
| Mali | 245 (183-321) | 203 (131-299) | -0.17 (-0.49-0.31) | 7.09 (5.27-9.37) | 2.42 (1.54-3.63) | -3.83 (-4.14--3.52) |
| Malta | 0 (0-0) | 0 (0-0) | -0.83 (-0.88--0.74) | 0.05 (0.04-0.06) | 0.01 (0.01-0.01) | -5.66 (-6.29--5.03) |
| Marshall Islands | 0 (0-1) | 0 (0-0) | -0.47 (-0.7--0.15) | 2.71 (1.93-3.78) | 0.81 (0.49-1.25) | -4.57 (-5.12--4.02) |
| Mauritania | 234 (182-293) | 59 (36-89) | -0.75 (-0.85--0.6) | 29.36 (22.45-37.37) | 3.39 (2.06-5.15) | -7.24 (-7.6--6.89) |
| Mauritius | 6 (5-7) | 1 (0-1) | -0.9 (-0.93--0.85) | 0.93 (0.76-1.12) | 0.09 (0.06-0.13) | -6.43 (-8.06--4.76) |
| Mexico | 126 (98-162) | 46 (33-63) | -0.64 (-0.76--0.45) | 0.3 (0.23-0.38) | 0.07 (0.05-0.09) | -4.59 (-4.91--4.28) |
| Micronesia (Federated States of) | 2 (1-3) | 0 (0-0) | -0.86 (-0.95--0.74) | 5.16 (3.45-7.54) | 0.57 (0.18-0.99) | -7.79 (-8--7.58) |
| Monaco | 0 (0-0) | 0 (0-0) | -0.89 (-0.94--0.8) | 0 (0-0.01) | 0 (0-0) | -7.76 (-8.9--6.6) |
| Mongolia | 43 (31-58) | 4 (2-5) | -0.92 (-0.95--0.86) | 4.53 (3.3-6.01) | 0.18 (0.12-0.28) | -12.43 (-13.25--11.6) |
| Montenegro | 0 (0-0) | 0 (0-0) | -0.78 (-0.88--0.64) | 0.09 (0.06-0.13) | 0.02 (0.01-0.03) | -5.52 (-6.3--4.72) |
| Morocco | 1027 (767-1334) | 38 (25-61) | -0.96 (-0.98--0.94) | 8.62 (6.48-11.08) | 0.2 (0.13-0.32) | -12.33 (-12.68--11.98) |
| Mozambique | 541 (388-729) | 162 (97-242) | -0.7 (-0.82--0.53) | 8.97 (6.47-12.09) | 1.18 (0.69-1.76) | -5.96 (-6.34--5.58) |
| Myanmar | 979 (675-1408) | 241 (153-357) | -0.75 (-0.86--0.6) | 4.67 (3.24-6.74) | 0.8 (0.51-1.18) | -5.92 (-6.07--5.77) |
| Namibia | 22 (13-30) | 4 (2-7) | -0.8 (-0.9--0.61) | 3.7 (2.2-5.18) | 0.35 (0.19-0.59) | -7.92 (-8.61--7.23) |
| Nauru | 0 (0-0) | 0 (0-0) | -0.71 (-0.84--0.5) | 2.16 (1.36-3.32) | 0.52 (0.31-0.87) | -4.86 (-5.59--4.13) |
| Nepal | 1350 (986-1815) | 46 (29-69) | -0.97 (-0.98--0.95) | 15.27 (11.17-20.73) | 0.27 (0.17-0.41) | -13.63 (-14.02--13.24) |
| Netherlands | 1 (1-1) | 0 (0-0) | -0.93 (-0.96--0.9) | 0.01 (0.01-0.02) | 0 (0-0) | -11 (-11.83--10.16) |
| New Zealand | 0 (0-0) | 0 (0-0) | -0.78 (-0.85--0.66) | 0.01 (0.01-0.01) | 0 (0-0) | -6.42 (-7.25--5.58) |
| Nicaragua | 25 (19-32) | 2 (1-3) | -0.93 (-0.95--0.89) | 1.39 (1.07-1.78) | 0.05 (0.03-0.07) | -10.91 (-11.82--9.98) |
| Niger | 681 (497-868) | 1173 (776-1664) | 0.72 (0.12-1.6) | 22.07 (16.22-28.34) | 14.02 (9.2-19.75) | -1.68 (-2.02--1.33) |
| Nigeria | 2821 (1702-4393) | 2555 (1530-4082) | -0.09 (-0.49-0.6) | 8.55 (5.1-13.46) | 2.82 (1.69-4.54) | -3.7 (-4.07--3.33) |
| Niue | 0 (0-0) | 0 (0-0) | -0.78 (-0.89--0.58) | 0.48 (0.28-0.76) | 0.13 (0.07-0.22) | -5.22 (-5.4--5.03) |
| North Macedonia | 1 (0-1) | 0 (0-0) | -0.88 (-0.93--0.8) | 0.05 (0.04-0.07) | 0.01 (0-0.01) | -8.12 (-8.63--7.6) |
| Northern Mariana Islands | 0 (0-0) | 0 (0-0) | -0.78 (-0.88--0.61) | 0.76 (0.5-1.12) | 0.19 (0.12-0.29) | -4.1 (-4.67--3.53) |
| Norway | 0 (0-0) | 0 (0-0) | -0.91 (-0.94--0.87) | 0.01 (0.01-0.01) | 0 (0-0) | -8.17 (-9.69--6.63) |
| Oman | 11 (8-17) | 0 (0-1) | -0.97 (-0.98--0.94) | 1.9 (1.23-2.86) | 0.02 (0.01-0.03) | -15.37 (-15.64--15.11) |
| Pakistan | 1306 (947-1760) | 474 (310-673) | -0.64 (-0.78--0.45) | 2.83 (2.04-3.81) | 0.43 (0.28-0.61) | -6.73 (-7.74--5.72) |
| Palau | 0 (0-0) | 0 (0-0) | -0.59 (-0.77--0.24) | 1.04 (0.65-1.6) | 0.34 (0.2-0.52) | -3.71 (-3.86--3.55) |
| Palestine | 1 (1-2) | 1 (1-1) | -0.45 (-0.68--0.06) | 0.19 (0.12-0.29) | 0.03 (0.02-0.05) | -4.4 (-5.3--3.49) |
| Panama | 7 (6-9) | 3 (2-4) | -0.63 (-0.77--0.42) | 0.58 (0.46-0.72) | 0.13 (0.08-0.19) | -4.58 (-4.96--4.2) |
| Papua New Guinea | 50 (33-70) | 31 (20-45) | -0.38 (-0.61-0.01) | 2.71 (1.79-3.81) | 0.63 (0.4-0.91) | -4.81 (-5.02--4.6) |
| Paraguay | 38 (29-48) | 17 (11-25) | -0.55 (-0.72--0.27) | 1.96 (1.54-2.51) | 0.45 (0.29-0.68) | -5.23 (-5.91--4.56) |
| Peru | 314 (240-407) | 27 (16-43) | -0.91 (-0.95--0.86) | 3.09 (2.35-4.04) | 0.15 (0.09-0.24) | -10.97 (-11.58--10.36) |
| Philippines | 302 (229-380) | 121 (80-171) | -0.6 (-0.74--0.37) | 1 (0.76-1.25) | 0.21 (0.14-0.3) | -4.95 (-5.18--4.72) |
| Poland | 14 (11-17) | 1 (1-1) | -0.94 (-0.96--0.92) | 0.07 (0.05-0.09) | 0 (0-0.01) | -10.73 (-11.54--9.92) |
| Portugal | 4 (3-5) | 0 (0-0) | -0.92 (-0.95--0.89) | 0.07 (0.06-0.09) | 0.01 (0-0.01) | -9.29 (-10.32--8.25) |
| Puerto Rico | 3 (3-4) | 0 (0-0) | -0.96 (-0.98--0.95) | 0.18 (0.15-0.22) | 0.01 (0-0.01) | -12.12 (-13.62--10.59) |
| Qatar | 2 (1-2) | 0 (0-1) | -0.73 (-0.85--0.52) | 0.96 (0.66-1.34) | 0.05 (0.03-0.07) | -9.8 (-10.3--9.3) |
| Republic of Korea | 13 (10-17) | 0 (0-1) | -0.97 (-0.98--0.96) | 0.05 (0.04-0.06) | 0 (0-0) | -12.24 (-13.11--11.36) |
| Republic of Moldova | 10 (8-12) | 0 (0-0) | -0.99 (-0.99--0.98) | 0.43 (0.34-0.52) | 0.01 (0-0.01) | -14.84 (-15.74--13.94) |
| Romania | 151 (136-167) | 4 (3-6) | -0.97 (-0.98--0.96) | 1.35 (1.22-1.5) | 0.05 (0.03-0.07) | -11.75 (-12.41--11.09) |
| Russian Federation | 164 (126-202) | 15 (10-22) | -0.91 (-0.94--0.86) | 0.21 (0.16-0.26) | 0.02 (0.01-0.03) | -8.39 (-8.68--8.09) |
| Rwanda | 256 (183-357) | 35 (22-53) | -0.86 (-0.93--0.77) | 8.71 (6.25-11.99) | 0.57 (0.35-0.85) | -10.12 (-10.95--9.29) |
| Saint Kitts and Nevis | 1 (0-1) | 0 (0-0) | -0.83 (-0.95--0.68) | 2.77 (2.11-3.52) | 0.31 (0.1-0.54) | -6.86 (-7.79--5.92) |
| Saint Lucia | 0 (0-0) | 0 (0-0) | -0.85 (-0.9--0.77) | 0.46 (0.37-0.58) | 0.05 (0.04-0.07) | -6.24 (-7.25--5.22) |
| Saint Vincent and the Grenadines | 0 (0-0) | 0 (0-0) | -0.7 (-0.8--0.54) | 0.39 (0.3-0.49) | 0.11 (0.08-0.16) | -4.34 (-4.91--3.77) |
| Samoa | 1 (0-1) | 0 (0-0) | -0.84 (-0.93--0.71) | 1.01 (0.65-1.56) | 0.11 (0.05-0.18) | -8.09 (-8.54--7.63) |
| San Marino | 0 (0-0) | 0 (0-0) | -0.52 (-0.77--0.05) | 0.01 (0-0.01) | 0 (0-0) | -3.98 (-4.26--3.7) |
| Sao Tome and Principe | 1 (1-2) | 0 (0-0) | -0.74 (-0.85--0.46) | 2.9 (1.51-4.41) | 0.33 (0.21-0.5) | -8.2 (-9.11--7.29) |
| Saudi Arabia | 48 (30-70) | 4 (3-7) | -0.91 (-0.95--0.84) | 0.82 (0.52-1.21) | 0.02 (0.01-0.03) | -11.6 (-11.93--11.26) |
| Senegal | 407 (295-525) | 383 (245-545) | -0.06 (-0.42-0.43) | 13.37 (9.61-17.25) | 5.68 (3.66-8.25) | -2.36 (-2.62--2.1) |
| Serbia | 1 (1-1) | 0 (0-0) | -0.9 (-0.94--0.83) | 0.02 (0.01-0.02) | 0 (0-0) | -7.63 (-8.1--7.16) |
| Seychelles | 0 (0-0) | 0 (0-0) | -0.83 (-0.89--0.75) | 1 (0.75-1.32) | 0.11 (0.08-0.15) | -6.76 (-7.5--6.01) |
| Sierra Leone | 184 (123-256) | 229 (152-322) | 0.24 (-0.22-0.95) | 11.6 (7.77-16.23) | 5.89 (3.89-8.36) | -1.81 (-2.59--1.03) |
| Singapore | 0 (0-0) | 0 (0-0) | -0.93 (-0.95--0.89) | 0.01 (0.01-0.02) | 0 (0-0) | -11.08 (-12.08--10.07) |
| Slovakia | 1 (1-1) | 0 (0-0) | -0.91 (-0.95--0.85) | 0.03 (0.02-0.04) | 0 (0-0) | -8.51 (-9.34--7.67) |
| Slovenia | 0 (0-0) | 0 (0-0) | -0.89 (-0.94--0.81) | 0.02 (0.02-0.03) | 0 (0-0) | -5.71 (-7.43--3.95) |
| Solomon Islands | 20 (13-28) | 11 (7-16) | -0.45 (-0.68--0.1) | 15.58 (10.17-22.39) | 3.55 (2.22-5.23) | -4.99 (-5.27--4.71) |
| Somalia | 750 (465-1066) | 929 (593-1378) | 0.24 (-0.16-0.86) | 26.06 (16.07-37.33) | 11.34 (7.2-16.91) | -2.76 (-2.98--2.54) |
| South Africa | 288 (217-387) | 56 (32-86) | -0.81 (-0.9--0.68) | 1.48 (1.12-1.97) | 0.17 (0.1-0.26) | -6.19 (-8.01--4.33) |
| South Sudan | 356 (229-528) | 268 (157-444) | -0.25 (-0.56-0.33) | 15.41 (9.89-22.65) | 6.53 (3.82-10.78) | -3.01 (-3.13--2.89) |
| Spain | 8 (7-10) | 1 (0-1) | -0.94 (-0.96--0.91) | 0.04 (0.03-0.05) | 0 (0-0) | -10.73 (-11.26--10.19) |
| Sri Lanka | 29 (21-39) | 4 (2-5) | -0.88 (-0.93--0.79) | 0.31 (0.23-0.42) | 0.03 (0.02-0.05) | -7.63 (-7.96--7.31) |
| Sudan | 505 (350-716) | 72 (41-116) | -0.86 (-0.92--0.76) | 5.69 (3.99-8.07) | 0.36 (0.2-0.58) | -8.84 (-9.37--8.31) |
| Suriname | 1 (0-1) | 0 (0-0) | -0.58 (-0.75--0.26) | 0.39 (0.24-0.55) | 0.11 (0.08-0.16) | -4.31 (-4.82--3.79) |
| Sweden | 0 (0-0) | 0 (0-0) | -0.81 (-0.87--0.71) | 0.01 (0-0.01) | 0 (0-0) | -7.05 (-8.32--5.76) |
| Switzerland | 0 (0-0) | 0 (0-0) | -0.77 (-0.85--0.65) | 0.01 (0-0.01) | 0 (0-0) | -6.87 (-7.79--5.93) |
| Syrian Arab Republic | 34 (23-49) | 1 (1-2) | -0.97 (-0.98--0.95) | 0.75 (0.5-1.07) | 0.01 (0.01-0.02) | -13.52 (-14.13--12.9) |
| Taiwan (Province of China) | 3 (3-4) | 2 (1-2) | -0.54 (-0.71--0.26) | 0.03 (0.02-0.04) | 0.01 (0.01-0.02) | -2.99 (-3.41--2.56) |
| Tajikistan | 12 (8-16) | 1 (1-2) | -0.9 (-0.94--0.82) | 0.47 (0.33-0.65) | 0.02 (0.01-0.04) | -11.31 (-11.99--10.62) |
| Thailand | 325 (234-430) | 24 (15-37) | -0.92 (-0.96--0.87) | 0.99 (0.72-1.33) | 0.06 (0.04-0.1) | -9.84 (-10.33--9.34) |
| Timor-Leste | 99 (66-132) | 10 (3-14) | -0.9 (-0.97--0.83) | 29.21 (19.66-38.74) | 1.74 (0.46-2.63) | -10.35 (-10.8--9.9) |
| Togo | 134 (95-176) | 63 (38-93) | -0.53 (-0.72--0.27) | 9.06 (6.43-12.08) | 1.65 (0.99-2.45) | -5.7 (-6.38--5.02) |
| Tokelau | 0 (0-0) | 0 (0-0) | -0.88 (-0.94--0.77) | 2.8 (1.72-4.45) | 0.36 (0.21-0.58) | -7.33 (-7.45--7.2) |
| Tonga | 1 (0-1) | 0 (0-0) | -0.71 (-0.83--0.52) | 1.57 (1.11-2.16) | 0.36 (0.23-0.56) | -5.55 (-5.75--5.36) |
| Trinidad and Tobago | 8 (7-9) | 1 (0-1) | -0.91 (-0.94--0.85) | 1.25 (1.07-1.46) | 0.1 (0.06-0.16) | -9.41 (-10.19--8.63) |
| Tunisia | 47 (34-64) | 1 (1-2) | -0.97 (-0.98--0.95) | 1.16 (0.84-1.55) | 0.02 (0.01-0.04) | -12.89 (-13.31--12.46) |
| Turkey | 304 (223-407) | 6 (4-9) | -0.98 (-0.99--0.97) | 1.05 (0.78-1.44) | 0.01 (0.01-0.02) | -16.14 (-17.34--14.91) |
| Turkmenistan | 14 (11-17) | 7 (5-10) | -0.49 (-0.66--0.23) | 0.83 (0.65-1.05) | 0.28 (0.18-0.41) | -3.88 (-4.3--3.45) |
| Tuvalu | 0 (0-0) | 0 (0-0) | -0.89 (-0.94--0.81) | 3.66 (2.46-5.08) | 0.36 (0.21-0.55) | -7.95 (-8.15--7.75) |
| Uganda | 214 (130-318) | 164 (104-244) | -0.23 (-0.56-0.4) | 2.99 (1.87-4.43) | 0.88 (0.57-1.3) | -4.38 (-5.17--3.59) |
| Ukraine | 9 (6-12) | 2 (1-2) | -0.82 (-0.89--0.72) | 0.03 (0.02-0.05) | 0.01 (0-0.01) | -6.08 (-6.9--5.26) |
| United Arab Emirates | 2 (1-2) | 1 (0-1) | -0.55 (-0.76--0.17) | 0.26 (0.16-0.38) | 0.02 (0.01-0.03) | -8.86 (-9.44--8.27) |
| United Kingdom | 5 (4-6) | 1 (1-1) | -0.83 (-0.88--0.75) | 0.02 (0.01-0.02) | 0 (0-0) | -6.94 (-7.51--6.36) |
| United Republic of Tanzania | 723 (513-994) | 333 (213-488) | -0.54 (-0.73--0.21) | 6.26 (4.42-8.58) | 1.24 (0.8-1.82) | -4.59 (-5.02--4.15) |
| United States of America | 33 (26-42) | 17 (13-22) | -0.49 (-0.64--0.28) | 0.02 (0.02-0.03) | 0.01 (0.01-0.01) | -2.1 (-2.42--1.77) |
| United States Virgin Islands | 0 (0-0) | 0 (0-0) | -0.86 (-0.92--0.77) | 0.39 (0.28-0.54) | 0.07 (0.04-0.1) | -5.54 (-5.95--5.13) |
| Uruguay | 6 (5-8) | 2 (2-2) | -0.7 (-0.78--0.61) | 0.43 (0.36-0.5) | 0.11 (0.09-0.14) | -5.7 (-6.33--5.06) |
| Uzbekistan | 16 (12-20) | 7 (5-9) | -0.58 (-0.73--0.39) | 0.21 (0.15-0.27) | 0.04 (0.03-0.05) | -5.11 (-5.84--4.37) |
| Vanuatu | 2 (1-3) | 1 (1-2) | -0.35 (-0.68-0.17) | 3.6 (2.08-5.5) | 1.07 (0.57-1.65) | -4.83 (-5.08--4.58) |
| Venezuela (Bolivarian Republic of) | 92 (72-115) | 39 (26-57) | -0.58 (-0.74--0.34) | 0.93 (0.73-1.15) | 0.26 (0.18-0.39) | -3.81 (-4.7--2.9) |
| Viet Nam | 145 (86-228) | 3 (2-4) | -0.98 (-0.99--0.96) | 0.43 (0.26-0.66) | 0.01 (0-0.01) | -14.96 (-15.48--14.44) |
| Yemen | 927 (516-1412) | 467 (258-715) | -0.5 (-0.73--0.04) | 17.94 (10.35-26.92) | 3.04 (1.71-4.59) | -6.07 (-6.46--5.68) |
| Zambia | 227 (166-296) | 64 (41-92) | -0.72 (-0.83--0.56) | 6.35 (4.64-8.28) | 0.69 (0.44-0.99) | -8.08 (-8.64--7.51) |
| Zimbabwe | 129 (94-170) | 128 (85-188) | -0.01 (-0.37-0.56) | 3.03 (2.21-4.02) | 1.72 (1.12-2.51) | 1.17 (-0.33-2.69) |

Abbreviations: ASDR: age-standardized death rate; EAPC: estimated annual percentage change; CI: confidence interval; UI: uncertainty interval

# TableS5.The DALY cases and age-standardized rates of Maternal abortion and miscarriage in 1990 and 2019, for female, in 204 countries and territories

| location | 1990  Number  (95% UI) | 2019  Number  (95% UI) | Change of number  from 1990 to 2019  (95% UI) | 1990  ASR (per100,000)  (95% UI) | 2019  ASR (per100,000)  (95% UI) | EAPC in ASR  (%, 95% CI) |
| --- | --- | --- | --- | --- | --- | --- |
| Afghanistan | 14487 (10165-20305) | 9103 (6175-13402) | -0.37 (-0.59--0.04) | 312.46 (214.34-443.34) | 55.56 (37.61-80.2) | -5.75 (-6.46--5.03) |
| Albania | 600 (473-755) | 24 (16-34) | -0.96 (-0.97--0.94) | 34.99 (27.88-43.42) | 1.87 (1.24-2.62) | -8.87 (-9.8--7.92) |
| Algeria | 5313 (3637-7688) | 443 (290-630) | -0.92 (-0.95--0.86) | 43.73 (30.35-63.05) | 1.93 (1.28-2.73) | -10.22 (-10.77--9.66) |
| American Samoa | 8 (6-10) | 2 (1-3) | -0.72 (-0.83--0.53) | 35.83 (25.35-47.76) | 8.21 (5.54-12) | -5.44 (-6.13--4.73) |
| Andorra | 0 (0-0) | 0 (0-0) | -0.41 (-0.59--0.2) | 0.63 (0.44-0.87) | 0.28 (0.17-0.42) | -2.95 (-3.04--2.86) |
| Angola | 45196 (30567-61416) | 21036 (12938-30434) | -0.53 (-0.73--0.23) | 1043.58 (702.71-1412.51) | 158.57 (97.39-234.56) | -6.13 (-6.59--5.66) |
| Antigua and Barbuda | 6 (5-8) | 2 (2-3) | -0.65 (-0.75--0.5) | 17.67 (13.78-22.54) | 4.58 (3.4-6.02) | -4.25 (-4.84--3.66) |
| Argentina | 9603 (7960-11423) | 4367 (3509-5487) | -0.55 (-0.66--0.4) | 59.62 (49.42-71.01) | 18.56 (14.92-23.32) | -4.04 (-4.25--3.82) |
| Armenia | 205 (156-264) | 40 (28-55) | -0.81 (-0.87--0.72) | 11.23 (8.62-14.42) | 2.65 (1.81-3.66) | -4.54 (-5.36--3.71) |
| Australia | 119 (72-183) | 87 (42-151) | -0.27 (-0.59-0.12) | 1.34 (0.81-2.08) | 0.76 (0.38-1.33) | -1.68 (-2--1.37) |
| Austria | 110 (75-154) | 39 (20-68) | -0.65 (-0.79--0.45) | 2.72 (1.85-3.8) | 1 (0.5-1.75) | -3.63 (-4.09--3.17) |
| Azerbaijan | 819 (635-1061) | 108 (69-159) | -0.87 (-0.92--0.8) | 22.16 (17.52-28.09) | 2.04 (1.29-3.01) | -9.51 (-10.05--8.97) |
| Bahamas | 49 (39-60) | 16 (11-22) | -0.68 (-0.78--0.53) | 32.12 (25.55-39.44) | 7.65 (5.46-10.71) | -4.78 (-5.48--4.07) |
| Bahrain | 69 (51-89) | 23 (16-33) | -0.67 (-0.79--0.49) | 28.94 (21.49-37.83) | 3.36 (2.38-4.76) | -7 (-7.62--6.38) |
| Bangladesh | 235467 (163104-306113) | 9095 (6244-12758) | -0.96 (-0.98--0.94) | 502.5 (344.63-656.96) | 10.3 (7.08-14.52) | -12.67 (-13.25--12.09) |
| Barbados | 22 (17-28) | 7 (5-9) | -0.71 (-0.8--0.58) | 15.69 (12.17-19.44) | 4.9 (3.48-6.55) | -3.91 (-4.42--3.4) |
| Belarus | 429 (333-543) | 75 (45-115) | -0.82 (-0.89--0.75) | 8.18 (6.35-10.37) | 1.75 (1.04-2.63) | -6.4 (-7.15--5.64) |
| Belgium | 108 (83-141) | 30 (15-52) | -0.72 (-0.85--0.55) | 2.17 (1.68-2.82) | 0.6 (0.3-1.06) | -5.1 (-5.86--4.32) |
| Belize | 26 (19-33) | 24 (19-32) | -0.06 (-0.33-0.31) | 27.81 (21.1-34.84) | 10.31 (7.88-13.48) | -3.32 (-3.5--3.13) |
| Benin | 16365 (12245-21386) | 12408 (7630-19272) | -0.24 (-0.54-0.21) | 819.15 (607.37-1066.96) | 222.76 (138.82-344.21) | -3.84 (-4.13--3.56) |
| Bermuda | 3 (2-4) | 0 (0-1) | -0.87 (-0.92--0.79) | 7.94 (5.97-10.43) | 1.37 (0.76-2.15) | -5.41 (-6.15--4.66) |
| Bhutan | 519 (288-789) | 19 (12-30) | -0.96 (-0.98--0.93) | 196.04 (108.49-290.27) | 4.65 (2.8-7.2) | -12.92 (-13.18--12.67) |
| Bolivia (Plurinational State of) | 15465 (11752-19927) | 3029 (1887-4535) | -0.8 (-0.88--0.69) | 523.43 (398.31-677.91) | 50.45 (31.58-75.67) | -8.37 (-8.76--7.98) |
| Bosnia and Herzegovina | 352 (283-432) | 6 (4-9) | -0.98 (-0.99--0.98) | 14.5 (11.68-17.72) | 0.38 (0.24-0.59) | -15.12 (-16.89--13.31) |
| Botswana | 320 (207-462) | 87 (51-134) | -0.73 (-0.85--0.54) | 51.16 (32.53-74.66) | 6.38 (3.77-9.83) | -6.86 (-7.54--6.18) |
| Brazil | 25624 (20493-31271) | 6412 (4951-8010) | -0.75 (-0.82--0.66) | 32.89 (26.49-39.88) | 5.56 (4.31-6.95) | -5.15 (-5.65--4.65) |
| Brunei Darussalam | 14 (10-19) | 3 (2-4) | -0.78 (-0.86--0.66) | 9.82 (6.89-13.85) | 1.2 (0.85-1.66) | -6.75 (-7.58--5.91) |
| Bulgaria | 674 (550-803) | 39 (27-53) | -0.94 (-0.96--0.92) | 16.3 (13.25-19.54) | 1.33 (0.93-1.77) | -10.27 (-11.27--9.25) |
| Burkina Faso | 14126 (9482-19674) | 12598 (8076-18269) | -0.11 (-0.45-0.39) | 343.82 (231.67-478.12) | 117.12 (75.09-168.83) | -4.1 (-4.43--3.76) |
| Burundi | 24975 (17326-34978) | 11551 (7954-15732) | -0.54 (-0.7--0.28) | 1029.21 (717.58-1428.12) | 219.77 (149.95-305.19) | -6.13 (-6.6--5.66) |
| Cabo Verde | 273 (201-358) | 27 (18-38) | -0.9 (-0.94--0.85) | 160.05 (120.89-206.71) | 8.76 (5.98-12.51) | -9.62 (-10.32--8.91) |
| Cambodia | 61783 (46449-80551) | 4523 (3084-6410) | -0.93 (-0.95--0.89) | 1307.55 (981.75-1711.62) | 52.42 (35.66-75.17) | -11.93 (-12.54--11.32) |
| Cameroon | 15166 (11169-20143) | 13088 (7573-19712) | -0.14 (-0.49-0.33) | 338.71 (248.09-448.78) | 92.89 (54.59-140.7) | -4.11 (-4.78--3.43) |
| Canada | 151 (116-194) | 99 (58-157) | -0.35 (-0.6-0.01) | 1 (0.77-1.27) | 0.61 (0.36-0.97) | -2 (-2.39--1.62) |
| Central African Republic | 16585 (12427-21218) | 13177 (8857-19806) | -0.21 (-0.47-0.19) | 1365.2 (1032.24-1758.81) | 530.35 (349.79-795.44) | -3.09 (-3.46--2.73) |
| Chad | 30909 (22887-39453) | 65611 (44178-87144) | 1.12 (0.39-2.05) | 1207.15 (891.41-1551.24) | 958.79 (658.31-1286.7) | -0.48 (-1.05-0.1) |
| Chile | 2478 (2026-3024) | 147 (111-189) | -0.94 (-0.96--0.92) | 33 (26.95-40.1) | 1.6 (1.21-2.06) | -8.79 (-9.85--7.73) |
| China | 69123 (55295-85089) | 5600 (3463-8377) | -0.92 (-0.95--0.88) | 9.99 (8-12.28) | 0.88 (0.54-1.32) | -8.03 (-8.68--7.38) |
| Colombia | 6673 (5156-8497) | 1551 (1069-2120) | -0.77 (-0.85--0.66) | 36.36 (28.42-45.97) | 6.14 (4.24-8.44) | -5.93 (-6.55--5.31) |
| Comoros | 886 (188-1450) | 111 (58-168) | -0.87 (-0.94--0.38) | 467.14 (108.23-761.83) | 31.03 (16.42-47.55) | -10.13 (-10.82--9.43) |
| Congo | 4838 (3423-6574) | 2086 (1323-3137) | -0.57 (-0.74--0.3) | 463.78 (326.19-634.5) | 78.03 (49.39-117.18) | -5.9 (-6.65--5.16) |
| Cook Islands | 0 (0-0) | 0 (0-0) | -0.7 (-0.83--0.52) | 2.76 (1.85-3.85) | 0.85 (0.48-1.31) | -3.7 (-4.03--3.38) |
| Costa Rica | 369 (298-445) | 103 (72-141) | -0.72 (-0.81--0.6) | 23.07 (18.75-27.65) | 3.97 (2.78-5.46) | -7.28 (-8.1--6.45) |
| Croatia | 49 (38-62) | 7 (4-10) | -0.86 (-0.92--0.79) | 2.02 (1.57-2.54) | 0.37 (0.22-0.57) | -6.93 (-7.83--6.01) |
| Cuba | 1589 (1277-1970) | 283 (203-380) | -0.82 (-0.87--0.75) | 25.22 (20.36-30.93) | 5.89 (4.26-7.97) | -4.85 (-5.21--4.48) |
| Cyprus | 7 (4-10) | 3 (1-5) | -0.58 (-0.76--0.32) | 1.7 (1.09-2.48) | 0.38 (0.19-0.66) | -5.52 (-6.03--5.01) |
| Czechia | 156 (121-200) | 19 (9-33) | -0.88 (-0.94--0.78) | 2.99 (2.28-3.83) | 0.42 (0.2-0.74) | -4.9 (-6.11--3.68) |
| Côte d'Ivoire | 24871 (17473-33452) | 24720 (15385-36611) | -0.01 (-0.43-0.55) | 497.59 (345.55-672.45) | 204.55 (127.02-299.6) | -2.64 (-3.21--2.07) |
| Democratic People's Republic of Korea | 14748 (9099-22296) | 1461 (868-2248) | -0.9 (-0.94--0.82) | 131.94 (81.74-200.39) | 11.07 (6.59-17.15) | -8.49 (-9.17--7.82) |
| Democratic Republic of the Congo | 99344 (69990-134326) | 82813 (59202-112191) | -0.17 (-0.45-0.27) | 627.95 (446.6-856.86) | 222.27 (155.34-305.32) | -2.54 (-2.97--2.1) |
| Denmark | 26 (16-40) | 21 (10-35) | -0.22 (-0.59-0.33) | 1.03 (0.62-1.58) | 0.83 (0.38-1.43) | -1.92 (-2.5--1.33) |
| Djibouti | 1959 (1236-2768) | 1294 (637-2037) | -0.34 (-0.65-0.19) | 913.26 (579.95-1289.81) | 205.52 (101.07-324.03) | -5.25 (-5.77--4.72) |
| Dominica | 23 (17-30) | 8 (6-12) | -0.64 (-0.78--0.41) | 62.9 (47.53-83.01) | 25.15 (16.69-36.7) | -3.22 (-3.48--2.96) |
| Dominican Republic | 2107 (1589-2746) | 1206 (777-1773) | -0.43 (-0.65--0.11) | 52.82 (40.1-67.54) | 20.68 (13.3-30.26) | -2.3 (-3.07--1.52) |
| Ecuador | 3009 (2341-3792) | 1033 (738-1445) | -0.66 (-0.77--0.48) | 58.49 (45.66-73.69) | 10.97 (7.83-15.4) | -5.17 (-5.9--4.44) |
| Egypt | 14045 (10717-17780) | 929 (598-1327) | -0.93 (-0.96--0.9) | 53.62 (41.17-67.76) | 1.78 (1.15-2.52) | -12.3 (-13.04--11.55) |
| El Salvador | 5727 (4544-6978) | 275 (181-410) | -0.95 (-0.97--0.92) | 210.8 (168.36-254.39) | 7.74 (5.07-11.62) | -10.7 (-11.8--9.58) |
| Equatorial Guinea | 2053 (1415-2830) | 196 (102-343) | -0.9 (-0.95--0.82) | 1104.21 (755.95-1526.64) | 30.76 (15.92-54.3) | -12.78 (-13.22--12.33) |
| Eritrea | 18255 (11840-25481) | 7368 (4819-11523) | -0.6 (-0.75--0.32) | 1345.85 (877.67-1888.26) | 217.34 (143.17-341.47) | -6.11 (-6.51--5.71) |
| Estonia | 66 (50-83) | 5 (3-9) | -0.92 (-0.96--0.86) | 8.76 (6.68-11.23) | 0.95 (0.45-1.62) | -9.06 (-10.16--7.95) |
| Eswatini | 443 (305-624) | 116 (59-199) | -0.74 (-0.86--0.53) | 126.22 (84.76-178.4) | 19.46 (9.77-33.47) | -5.52 (-6.74--4.28) |
| Ethiopia | 391716 (293794-535341) | 97802 (68215-136248) | -0.75 (-0.85--0.61) | 1863.06 (1404.18-2549.91) | 200.51 (141.37-276.83) | -7.89 (-8.5--7.29) |
| Fiji | 247 (172-347) | 92 (61-132) | -0.63 (-0.78--0.36) | 65.84 (46.17-92.7) | 19.97 (13.34-28.62) | -4.41 (-4.57--4.25) |
| Finland | 47 (36-58) | 10 (5-19) | -0.77 (-0.89--0.62) | 1.87 (1.44-2.34) | 0.46 (0.21-0.85) | -4.85 (-5.45--4.24) |
| France | 738 (552-970) | 271 (159-438) | -0.63 (-0.77--0.44) | 2.49 (1.87-3.27) | 0.97 (0.56-1.56) | -3.15 (-3.57--2.73) |
| Gabon | 1326 (958-1744) | 298 (177-451) | -0.78 (-0.87--0.65) | 326 (233.61-429.52) | 31.77 (18.83-48.34) | -8.03 (-8.65--7.41) |
| Gambia | 2273 (1467-3228) | 1939 (1313-2720) | -0.15 (-0.47-0.43) | 574.43 (372.07-830.48) | 194.03 (129.25-277.5) | -3.65 (-4.27--3.03) |
| Georgia | 149 (112-195) | 86 (62-115) | -0.42 (-0.6--0.16) | 5.22 (3.96-6.81) | 5.16 (3.74-6.84) | 2.81 (1.73-3.9) |
| Germany | 1125 (878-1424) | 220 (115-359) | -0.8 (-0.89--0.68) | 2.76 (2.15-3.5) | 0.63 (0.32-1.04) | -4.49 (-5.18--3.8) |
| Ghana | 12352 (8270-17181) | 4106 (2655-5746) | -0.67 (-0.81--0.47) | 170.22 (115.26-236.03) | 23.29 (15.18-32.33) | -6.14 (-6.35--5.93) |
| Greece | 91 (65-125) | 55 (38-75) | -0.4 (-0.58--0.14) | 1.81 (1.29-2.51) | 1.16 (0.79-1.65) | -1.19 (-1.87--0.51) |
| Greenland | 1 (0-1) | 0 (0-0) | -0.69 (-0.84--0.47) | 2.04 (1.38-2.88) | 0.71 (0.34-1.21) | -3.84 (-4.02--3.66) |
| Grenada | 14 (11-18) | 3 (2-4) | -0.8 (-0.86--0.71) | 33.66 (26.36-42.59) | 5.43 (4-7.16) | -5.65 (-6.26--5.04) |
| Guam | 5 (4-7) | 4 (3-6) | -0.2 (-0.48-0.19) | 7.66 (5.58-10.07) | 5.32 (3.74-7.24) | -1.5 (-2.09--0.91) |
| Guatemala | 11001 (8569-14102) | 2347 (1639-3230) | -0.79 (-0.86--0.69) | 303.4 (236.08-388.31) | 23.07 (16.09-31.79) | -9.89 (-10.84--8.94) |
| Guinea | 34057 (25961-43586) | 31393 (21954-43024) | -0.08 (-0.37-0.3) | 1259.22 (961.93-1615.42) | 535.11 (372.22-740.51) | -2.57 (-2.9--2.25) |
| Guinea-Bissau | 1221 (826-1731) | 387 (250-570) | -0.68 (-0.81--0.49) | 260.17 (175.14-366.04) | 38.98 (25.23-57.93) | -6.32 (-6.95--5.68) |
| Guyana | 483 (355-637) | 121 (81-174) | -0.75 (-0.84--0.6) | 111.14 (81.84-146.82) | 27.9 (18.69-40.22) | -4.67 (-4.97--4.37) |
| Haiti | 45885 (34589-61010) | 22879 (15508-31890) | -0.5 (-0.67--0.28) | 1524.39 (1150.03-2029.28) | 334.03 (227.02-468.22) | -4.65 (-4.93--4.37) |
| Honduras | 7705 (5733-10049) | 512 (314-780) | -0.93 (-0.96--0.89) | 355.26 (263.7-465.85) | 9.34 (5.62-14.27) | -12.63 (-13.22--12.04) |
| Hungary | 151 (115-195) | 30 (20-43) | -0.8 (-0.87--0.71) | 2.99 (2.28-3.87) | 0.71 (0.47-1.01) | -3.46 (-4.57--2.33) |
| Iceland | 1 (1-2) | 1 (0-2) | -0.17 (-0.6-0.49) | 0.8 (0.49-1.2) | 0.56 (0.24-1.01) | -0.84 (-0.96--0.73) |
| India | 794230 (591148-1020264) | 86493 (61146-116591) | -0.89 (-0.93--0.84) | 192.33 (144.8-246.64) | 11.65 (8.26-15.64) | -9.96 (-10.75--9.17) |
| Indonesia | 227121 (169232-296532) | 13913 (9881-19568) | -0.94 (-0.96--0.91) | 243.27 (182.76-314.67) | 9.85 (6.99-13.92) | -10.93 (-11.09--10.77) |
| Iran (Islamic Republic of) | 5940 (4627-7383) | 956 (714-1254) | -0.84 (-0.89--0.77) | 22.37 (17.37-28.18) | 2.09 (1.54-2.78) | -7.36 (-7.79--6.93) |
| Iraq | 2243 (1537-3119) | 570 (379-813) | -0.75 (-0.85--0.59) | 29.95 (20.57-43) | 2.53 (1.69-3.62) | -8.16 (-8.51--7.82) |
| Ireland | 22 (18-28) | 10 (6-16) | -0.54 (-0.73--0.29) | 1.27 (1.01-1.6) | 0.43 (0.25-0.67) | -3.81 (-4.56--3.06) |
| Israel | 139 (110-175) | 60 (37-93) | -0.57 (-0.73--0.34) | 5.64 (4.46-7.1) | 1.39 (0.87-2.15) | -4.76 (-5.39--4.14) |
| Italy | 677 (516-881) | 244 (160-350) | -0.64 (-0.72--0.55) | 2.36 (1.79-3.08) | 1.05 (0.67-1.51) | -2.27 (-2.55--1.99) |
| Jamaica | 296 (231-370) | 134 (90-192) | -0.55 (-0.7--0.3) | 24.3 (19.07-30.44) | 8.42 (5.71-12.1) | -4.96 (-5.84--4.07) |
| Japan | 1587 (1053-2284) | 280 (171-418) | -0.82 (-0.86--0.78) | 2.75 (1.77-4.01) | 0.59 (0.36-0.9) | -7.43 (-8.26--6.59) |
| Jordan | 601 (439-798) | 157 (104-221) | -0.74 (-0.84--0.58) | 43.91 (31.35-59.93) | 2.73 (1.83-3.85) | -10.08 (-10.85--9.31) |
| Kazakhstan | 6212 (5198-7225) | 503 (367-654) | -0.92 (-0.94--0.89) | 71.56 (60.17-82.5) | 5.19 (3.81-6.74) | -10.35 (-11.22--9.46) |
| Kenya | 23092 (16681-30163) | 18119 (12419-25493) | -0.22 (-0.48-0.18) | 236 (172.34-309.16) | 71.43 (48.31-100.79) | -3.82 (-4.16--3.48) |
| Kiribati | 141 (96-194) | 59 (40-87) | -0.58 (-0.74--0.31) | 380.94 (262.27-525.68) | 95.94 (64.49-139.23) | -4.85 (-5--4.71) |
| Kuwait | 30 (22-39) | 22 (12-34) | -0.26 (-0.56-0.11) | 3.39 (2.59-4.39) | 0.76 (0.41-1.2) | -6.22 (-7.25--5.17) |
| Kyrgyzstan | 380 (292-478) | 131 (95-180) | -0.66 (-0.76--0.52) | 17.25 (13.35-21.65) | 3.77 (2.73-5.19) | -4.46 (-4.81--4.1) |
| Lao People's Democratic Republic | 16707 (11858-23686) | 1029 (649-1491) | -0.94 (-0.96--0.9) | 857.56 (609.15-1198.97) | 26.34 (16.81-38.06) | -11.63 (-12.37--10.87) |
| Latvia | 89 (67-113) | 17 (10-26) | -0.81 (-0.88--0.71) | 6.86 (5.22-8.83) | 2.07 (1.23-3.19) | -3.93 (-4.86--3) |
| Lebanon | 202 (139-281) | 51 (33-74) | -0.75 (-0.85--0.6) | 13.1 (8.9-18.25) | 1.95 (1.24-2.81) | -6.12 (-6.65--5.59) |
| Lesotho | 1497 (998-2092) | 988 (536-1574) | -0.34 (-0.65-0.18) | 188.26 (123.66-266.35) | 90.9 (49.31-146.81) | -0.46 (-1.56-0.66) |
| Liberia | 6933 (4928-9354) | 4455 (2862-6233) | -0.36 (-0.61-0) | 858.52 (605.7-1156.39) | 192.15 (122.26-272.07) | -4.71 (-5.17--4.25) |
| Libya | 244 (170-342) | 65 (42-95) | -0.73 (-0.84--0.58) | 15.93 (10.83-22.77) | 1.59 (1.02-2.31) | -7.22 (-7.83--6.61) |
| Lithuania | 202 (159-252) | 22 (11-36) | -0.89 (-0.94--0.83) | 10.85 (8.56-13.57) | 1.85 (0.92-3.11) | -5.96 (-6.82--5.08) |
| Luxembourg | 5 (4-6) | 2 (1-3) | -0.58 (-0.75--0.32) | 2.3 (1.74-2.9) | 0.64 (0.36-1.02) | -4.89 (-5.15--4.62) |
| Madagascar | 32794 (24960-42252) | 20879 (14849-28419) | -0.36 (-0.57--0.05) | 594.07 (455.47-761.06) | 160.23 (113.07-220.76) | -4.6 (-4.93--4.27) |
| Malawi | 22110 (16236-29663) | 6067 (3642-9155) | -0.73 (-0.84--0.54) | 512.85 (377.83-682.37) | 66.17 (39.89-98.89) | -6.92 (-7.43--6.4) |
| Malaysia | 2511 (1848-3296) | 546 (384-780) | -0.78 (-0.87--0.66) | 29.29 (21.41-38.55) | 3.29 (2.31-4.75) | -7.51 (-8.13--6.88) |
| Maldives | 243 (165-349) | 10 (7-14) | -0.96 (-0.97--0.93) | 285.72 (194.45-414.86) | 4.35 (3.09-6) | -13.94 (-14.64--13.24) |
| Mali | 13508 (10162-17658) | 11379 (7382-16813) | -0.16 (-0.48-0.33) | 377.72 (281.93-494.91) | 128.33 (82.23-188.49) | -3.82 (-4.12--3.52) |
| Malta | 6 (5-7) | 1 (1-2) | -0.78 (-0.84--0.69) | 2.97 (2.33-3.72) | 0.64 (0.49-0.83) | -4.36 (-4.81--3.91) |
| Marshall Islands | 24 (18-33) | 12 (8-19) | -0.5 (-0.71--0.2) | 141.27 (101.45-195.28) | 42.24 (26.03-64.74) | -4.57 (-5.12--4.01) |
| Mauritania | 12565 (9864-15602) | 3181 (1953-4759) | -0.75 (-0.85--0.6) | 1508.94 (1169.24-1893.21) | 174.71 (106.21-262.2) | -7.25 (-7.62--6.88) |
| Mauritius | 334 (268-408) | 34 (24-47) | -0.9 (-0.93--0.85) | 53.01 (43.08-64.16) | 5.26 (3.63-7.27) | -6.33 (-7.91--4.73) |
| Mexico | 8773 (6983-10968) | 3608 (2694-4784) | -0.59 (-0.7--0.43) | 19.65 (15.72-24.52) | 5.31 (3.96-7.04) | -4.03 (-4.29--3.77) |
| Micronesia (Federated States of) | 110 (75-159) | 15 (6-25) | -0.86 (-0.95--0.75) | 269.06 (182.52-388.1) | 30.02 (10.7-50.77) | -7.78 (-8.01--7.56) |
| Monaco | 0 (0-0) | 0 (0-0) | -0.44 (-0.72--0.01) | 0.8 (0.47-1.26) | 0.46 (0.2-0.86) | -1.88 (-2.14--1.63) |
| Mongolia | 2525 (1837-3390) | 225 (151-333) | -0.91 (-0.94--0.86) | 253.65 (185.71-337.31) | 11.83 (8.08-17.17) | -11.99 (-12.84--11.13) |
| Montenegro | 18 (13-25) | 5 (3-7) | -0.74 (-0.85--0.59) | 5.72 (4.12-7.77) | 1.64 (1.12-2.34) | -4.84 (-5.53--4.15) |
| Morocco | 58834 (44457-76345) | 2253 (1505-3537) | -0.96 (-0.98--0.94) | 478.15 (356.95-619.32) | 11.64 (7.81-18.21) | -12.16 (-12.5--11.81) |
| Mozambique | 32008 (22955-43085) | 10064 (6221-15044) | -0.69 (-0.81--0.51) | 514.7 (369.24-690.83) | 69.62 (42.46-103.04) | -5.89 (-6.26--5.52) |
| Myanmar | 58779 (40804-83899) | 13858 (8746-20570) | -0.76 (-0.87--0.62) | 274.37 (189.41-394.56) | 46.01 (28.98-68.41) | -5.99 (-6.13--5.85) |
| Namibia | 1229 (704-1711) | 251 (144-402) | -0.8 (-0.89--0.6) | 198.7 (118.43-277.51) | 20.1 (11.53-32.62) | -7.7 (-8.34--7.04) |
| Nauru | 5 (3-8) | 1 (1-2) | -0.71 (-0.83--0.51) | 112.14 (71.41-172.95) | 27.71 (17.29-45.09) | -4.78 (-5.5--4.05) |
| Nepal | 78156 (57168-105005) | 2853 (1914-4126) | -0.96 (-0.98--0.94) | 859.93 (628.69-1162.02) | 16.35 (10.9-23.83) | -13.5 (-13.87--13.12) |
| Netherlands | 118 (79-165) | 50 (21-91) | -0.57 (-0.79--0.29) | 1.45 (0.97-2.04) | 0.7 (0.29-1.25) | -2.66 (-2.88--2.43) |
| New Zealand | 26 (16-40) | 12 (6-20) | -0.54 (-0.72--0.29) | 1.45 (0.88-2.21) | 0.63 (0.3-1.02) | -2.97 (-3.2--2.74) |
| Nicaragua | 1597 (1221-2024) | 136 (96-186) | -0.92 (-0.94--0.87) | 84.32 (64.98-106.4) | 3.72 (2.64-5.06) | -10.41 (-11.26--9.54) |
| Niger | 38307 (28235-48834) | 66518 (44360-94690) | 0.74 (0.13-1.65) | 1181.37 (862.13-1516.68) | 747.92 (490.6-1061.98) | -1.69 (-2.04--1.35) |
| Nigeria | 155185 (93920-239920) | 137394 (82755-224821) | -0.11 (-0.5-0.57) | 444.83 (267.6-692.42) | 145.34 (87.65-232.92) | -3.75 (-4.13--3.37) |
| Niue | 0 (0-0) | 0 (0-0) | -0.78 (-0.88--0.58) | 25.52 (15.41-39.36) | 7.02 (3.93-11.49) | -5 (-5.17--4.82) |
| North Macedonia | 37 (27-49) | 7 (5-11) | -0.8 (-0.88--0.7) | 3.55 (2.62-4.65) | 0.7 (0.45-1.05) | -6.09 (-6.57--5.6) |
| Northern Mariana Islands | 10 (7-15) | 2 (1-3) | -0.79 (-0.88--0.63) | 39.87 (26.14-57.77) | 10.52 (6.91-15.29) | -3.97 (-4.52--3.4) |
| Norway | 25 (18-36) | 16 (8-28) | -0.36 (-0.58--0.13) | 1.21 (0.84-1.71) | 0.68 (0.34-1.17) | -2.77 (-3.26--2.29) |
| Oman | 649 (434-944) | 38 (25-54) | -0.94 (-0.97--0.9) | 102.94 (68.18-151.32) | 1.89 (1.27-2.63) | -13.66 (-13.98--13.34) |
| Pakistan | 79047 (57159-106917) | 29757 (20015-41533) | -0.62 (-0.76--0.43) | 166.96 (121.09-223.84) | 26.17 (17.41-36.89) | -6.61 (-7.58--5.63) |
| Palau | 4 (3-6) | 2 (1-2) | -0.62 (-0.78--0.32) | 53.36 (33.8-80.32) | 17.6 (10.84-26.66) | -3.64 (-3.79--3.49) |
| Palestine | 98 (68-138) | 72 (50-99) | -0.27 (-0.54-0.12) | 12.1 (8.41-17.4) | 2.79 (1.95-3.84) | -3.86 (-4.51--3.2) |
| Panama | 489 (390-608) | 204 (143-279) | -0.58 (-0.72--0.38) | 37.4 (29.69-45.86) | 9.8 (6.85-13.39) | -4.14 (-4.48--3.81) |
| Papua New Guinea | 2858 (1888-4033) | 1792 (1172-2623) | -0.37 (-0.61-0.01) | 151.08 (99.48-211.51) | 35.66 (23.18-52.02) | -4.73 (-4.94--4.52) |
| Paraguay | 2302 (1808-2932) | 1052 (690-1561) | -0.54 (-0.72--0.28) | 117.09 (92.15-149.18) | 27.74 (18.21-41.12) | -5.15 (-5.8--4.49) |
| Peru | 18218 (14081-23673) | 1871 (1240-2706) | -0.9 (-0.93--0.84) | 172.28 (132.2-222.63) | 10.42 (6.92-15.1) | -10.28 (-10.9--9.65) |
| Philippines | 18092 (13751-22791) | 7679 (5345-10686) | -0.58 (-0.72--0.35) | 57.87 (44.33-72.41) | 13.25 (9.24-18.39) | -4.66 (-4.9--4.42) |
| Poland | 867 (678-1070) | 98 (66-140) | -0.89 (-0.92--0.84) | 4.5 (3.54-5.48) | 0.54 (0.36-0.77) | -7.01 (-7.72--6.3) |
| Portugal | 239 (188-297) | 36 (23-53) | -0.85 (-0.9--0.77) | 4.76 (3.73-5.91) | 0.8 (0.51-1.23) | -6.33 (-7.2--5.45) |
| Puerto Rico | 222 (179-268) | 19 (11-30) | -0.91 (-0.95--0.87) | 11.55 (9.29-13.95) | 1.22 (0.71-1.95) | -8.04 (-8.79--7.28) |
| Qatar | 90 (63-126) | 28 (19-41) | -0.68 (-0.81--0.47) | 55.05 (38.71-75.4) | 3.13 (2.08-4.42) | -9.3 (-9.79--8.81) |
| Republic of Korea | 855 (658-1095) | 60 (40-88) | -0.93 (-0.95--0.89) | 3.22 (2.49-4.07) | 0.25 (0.16-0.37) | -8.76 (-9.71--7.8) |
| Republic of Moldova | 613 (492-741) | 26 (15-41) | -0.96 (-0.98--0.93) | 26.85 (21.41-32.65) | 1.48 (0.81-2.32) | -10.74 (-11.77--9.71) |
| Romania | 8993 (8065-9949) | 260 (184-348) | -0.97 (-0.98--0.96) | 80.59 (72.3-89.05) | 3.33 (2.34-4.48) | -11.22 (-11.88--10.55) |
| Russian Federation | 10737 (8502-13049) | 1654 (1164-2282) | -0.85 (-0.89--0.78) | 14.13 (11.16-17.07) | 2.45 (1.72-3.4) | -6.47 (-6.7--6.24) |
| Rwanda | 14653 (10523-20494) | 2072 (1317-3062) | -0.86 (-0.92--0.77) | 477.43 (343.81-656.77) | 32.19 (20.45-47.79) | -9.98 (-10.8--9.16) |
| Saint Kitts and Nevis | 35 (26-44) | 6 (2-10) | -0.82 (-0.93--0.68) | 161.85 (124.19-205.78) | 19.96 (7.7-32.94) | -6.59 (-7.49--5.69) |
| Saint Lucia | 21 (17-27) | 4 (3-5) | -0.83 (-0.88--0.75) | 29.61 (23.47-36.62) | 4.08 (2.92-5.5) | -5.82 (-6.68--4.95) |
| Saint Vincent and the Grenadines | 15 (12-19) | 5 (3-6) | -0.68 (-0.78--0.54) | 26.18 (20.81-32.35) | 8.59 (6.29-11.49) | -3.9 (-4.38--3.42) |
| Samoa | 41 (26-62) | 7 (3-11) | -0.83 (-0.92--0.7) | 57.06 (36.84-87.82) | 6.83 (3.41-10.66) | -7.84 (-8.28--7.39) |
| San Marino | 0 (0-0) | 0 (0-0) | -0.27 (-0.49-0.01) | 0.66 (0.46-0.91) | 0.37 (0.24-0.56) | -2.01 (-2.2--1.83) |
| Sao Tome and Principe | 70 (34-106) | 19 (12-27) | -0.73 (-0.84--0.43) | 153.46 (76.52-228.55) | 18.32 (11.97-26.83) | -8.1 (-9.01--7.18) |
| Saudi Arabia | 2828 (1819-4157) | 318 (212-458) | -0.89 (-0.93--0.81) | 46 (29.7-67.11) | 1.54 (1.02-2.2) | -10.82 (-11.17--10.47) |
| Senegal | 22944 (16589-29721) | 21200 (13758-29667) | -0.08 (-0.44-0.41) | 717.27 (519.21-921.07) | 302.63 (194.49-431.34) | -2.39 (-2.65--2.13) |
| Serbia | 64 (46-85) | 17 (10-27) | -0.73 (-0.84--0.58) | 1.41 (1.01-1.88) | 0.44 (0.26-0.7) | -3.37 (-3.83--2.9) |
| Seychelles | 19 (15-25) | 3 (2-5) | -0.83 (-0.88--0.74) | 55.76 (41.95-72.35) | 6.76 (4.92-9.21) | -6.36 (-7.12--5.6) |
| Sierra Leone | 10400 (6943-14497) | 13197 (8816-18359) | 0.27 (-0.2-0.98) | 626.44 (419.22-867.59) | 322.61 (215-451.45) | -1.77 (-2.55--0.99) |
| Singapore | 19 (14-25) | 3 (2-4) | -0.85 (-0.9--0.79) | 0.97 (0.73-1.27) | 0.09 (0.06-0.12) | -8.58 (-9.18--7.99) |
| Slovakia | 66 (47-89) | 17 (9-28) | -0.75 (-0.87--0.57) | 2.42 (1.71-3.27) | 0.66 (0.34-1.09) | -3.68 (-4.06--3.3) |
| Slovenia | 18 (13-26) | 5 (3-9) | -0.71 (-0.86--0.5) | 1.78 (1.24-2.52) | 0.62 (0.33-0.98) | -2 (-2.84--1.15) |
| Solomon Islands | 1106 (716-1576) | 593 (373-870) | -0.46 (-0.69--0.12) | 831.55 (537.34-1185.22) | 188.36 (117.83-277.33) | -5.01 (-5.28--4.73) |
| Somalia | 41371 (25913-58669) | 52041 (33712-76870) | 0.26 (-0.15-0.88) | 1410.04 (876.25-2016.42) | 609.69 (390.35-903.58) | -2.79 (-3.01--2.56) |
| South Africa | 17066 (12994-22969) | 3718 (2280-5492) | -0.78 (-0.88--0.65) | 85.79 (64.79-114.12) | 11.56 (7.18-16.97) | -5.69 (-7.44--3.9) |
| South Sudan | 20544 (13355-30507) | 15148 (8869-25108) | -0.26 (-0.57-0.28) | 842.87 (543.01-1242.82) | 358.91 (211.1-587.47) | -3.01 (-3.13--2.88) |
| Spain | 517 (417-634) | 155 (77-268) | -0.7 (-0.84--0.49) | 2.67 (2.15-3.28) | 0.77 (0.37-1.38) | -3.52 (-4.42--2.6) |
| Sri Lanka | 1794 (1289-2381) | 248 (165-357) | -0.86 (-0.91--0.78) | 18.91 (13.71-24.99) | 2.24 (1.49-3.22) | -7.11 (-7.44--6.77) |
| Sudan | 29636 (20492-41575) | 4473 (2725-6949) | -0.85 (-0.91--0.74) | 321.63 (223.82-456.73) | 21.42 (13.1-33.16) | -8.72 (-9.22--8.22) |
| Suriname | 51 (32-71) | 23 (16-32) | -0.56 (-0.73--0.25) | 24.88 (15.87-34.61) | 7.85 (5.59-11.15) | -3.98 (-4.49--3.48) |
| Sweden | 57 (34-90) | 33 (16-56) | -0.43 (-0.65--0.13) | 1.45 (0.85-2.28) | 0.75 (0.36-1.27) | -2.1 (-2.36--1.83) |
| Switzerland | 23 (14-36) | 24 (11-44) | 0.05 (-0.5-0.83) | 0.64 (0.39-1) | 0.6 (0.27-1.1) | -0.67 (-1.03--0.3) |
| Syrian Arab Republic | 2017 (1356-2783) | 103 (64-150) | -0.95 (-0.97--0.92) | 41.39 (27.87-58.25) | 1.3 (0.82-1.92) | -11.56 (-12.24--10.87) |
| Taiwan (Province of China) | 244 (185-325) | 104 (73-143) | -0.57 (-0.72--0.37) | 2.15 (1.65-2.81) | 0.85 (0.6-1.15) | -3.11 (-3.51--2.7) |
| Tajikistan | 766 (544-1036) | 165 (104-244) | -0.78 (-0.87--0.66) | 30.06 (21.3-40.5) | 3.13 (2.01-4.61) | -8.58 (-9.29--7.86) |
| Thailand | 19742 (14258-26440) | 1536 (981-2221) | -0.92 (-0.95--0.87) | 59.24 (43.23-78.09) | 4.35 (2.81-6.24) | -9.49 (-9.98--8.99) |
| Timor-Leste | 5384 (3552-7212) | 512 (142-755) | -0.9 (-0.97--0.83) | 1537.44 (1033.28-2053.91) | 89.62 (23.52-134.52) | -10.43 (-10.9--9.96) |
| Togo | 7425 (5295-9746) | 3448 (2094-5027) | -0.54 (-0.72--0.3) | 478.76 (343.32-632.45) | 87.92 (53.51-128.3) | -5.67 (-6.35--5) |
| Tokelau | 1 (1-2) | 0 (0-0) | -0.88 (-0.93--0.77) | 145.8 (91.21-230.7) | 19.27 (11.56-31.5) | -7.18 (-7.31--7.05) |
| Tonga | 31 (22-42) | 9 (6-14) | -0.7 (-0.82--0.52) | 79.49 (56.49-109.05) | 18.81 (12.36-28.93) | -5.38 (-5.57--5.19) |
| Trinidad and Tobago | 469 (396-550) | 48 (32-72) | -0.9 (-0.93--0.85) | 73.6 (62.75-85.88) | 6.94 (4.58-10.06) | -8.85 (-9.62--8.07) |
| Tunisia | 2950 (2144-3937) | 164 (105-240) | -0.94 (-0.97--0.91) | 69.78 (51.11-93.01) | 2.64 (1.69-3.88) | -10.89 (-11.47--10.31) |
| Turkey | 18784 (13832-25210) | 850 (537-1267) | -0.95 (-0.97--0.93) | 62.93 (46.62-84.39) | 1.94 (1.21-2.87) | -13.19 (-14.29--12.08) |
| Turkmenistan | 826 (643-1034) | 425 (295-602) | -0.49 (-0.65--0.25) | 47.98 (37.62-60.04) | 16.86 (11.78-23.79) | -3.69 (-4.11--3.26) |
| Tuvalu | 9 (6-13) | 1 (1-2) | -0.89 (-0.94--0.81) | 192.82 (130.66-267.32) | 18.78 (11.59-29.05) | -7.94 (-8.15--7.73) |
| Uganda | 12925 (7877-19318) | 10208 (6570-15089) | -0.21 (-0.54-0.43) | 169.94 (106.74-248.04) | 51.74 (33.48-76.07) | -4.31 (-5.09--3.53) |
| Ukraine | 898 (646-1243) | 333 (192-521) | -0.63 (-0.75--0.46) | 3.61 (2.57-5.02) | 1.7 (0.97-2.73) | -2.39 (-2.95--1.83) |
| United Arab Emirates | 100 (65-142) | 54 (34-80) | -0.46 (-0.68--0.11) | 14.96 (9.59-21.07) | 1.43 (0.93-2.07) | -7.64 (-8.19--7.09) |
| United Kingdom | 481 (364-619) | 218 (136-323) | -0.55 (-0.67--0.42) | 1.7 (1.29-2.2) | 0.73 (0.45-1.08) | -2.98 (-3.18--2.78) |
| United Republic of Tanzania | 43571 (30886-59675) | 20034 (13028-29254) | -0.54 (-0.72--0.23) | 358.13 (254.67-484.21) | 71.4 (46.55-103.58) | -4.58 (-5--4.16) |
| United States of America | 2843 (2219-3564) | 1401 (1097-1780) | -0.51 (-0.61--0.38) | 2.15 (1.68-2.71) | 0.93 (0.73-1.19) | -2.47 (-2.71--2.24) |
| United States Virgin Islands | 15 (11-19) | 3 (2-4) | -0.81 (-0.88--0.72) | 25.84 (19.2-34.55) | 6.29 (4.23-8.83) | -4.36 (-4.71--4.01) |
| Uruguay | 375 (317-441) | 117 (92-146) | -0.69 (-0.76--0.6) | 24.88 (21.03-29.29) | 6.94 (5.49-8.62) | -5.48 (-6.11--4.85) |
| Uzbekistan | 1104 (844-1418) | 618 (437-835) | -0.44 (-0.6--0.22) | 13.05 (10.07-16.57) | 3.37 (2.39-4.53) | -3.98 (-4.54--3.42) |
| Vanuatu | 123 (71-184) | 81 (43-123) | -0.35 (-0.68-0.14) | 190.66 (110.7-288.13) | 57.28 (31.04-87.27) | -4.79 (-5.04--4.54) |
| Venezuela (Bolivarian Republic of) | 5631 (4454-6968) | 2453 (1689-3566) | -0.56 (-0.72--0.32) | 55.57 (44.11-68.56) | 17.01 (11.68-24.61) | -3.53 (-4.4--2.65) |
| Viet Nam | 9201 (5580-14133) | 555 (322-887) | -0.94 (-0.97--0.89) | 25.92 (16.09-39.38) | 1.1 (0.64-1.73) | -10.99 (-11.68--10.28) |
| Yemen | 53052 (29281-81481) | 27020 (14887-40981) | -0.49 (-0.73--0.01) | 992.17 (557.78-1503.62) | 171.78 (95.95-260.08) | -5.98 (-6.38--5.58) |
| Zambia | 13919 (10103-18236) | 4118 (2736-5941) | -0.7 (-0.82--0.54) | 367.31 (271.39-475.68) | 42.35 (28.39-60.07) | -7.8 (-8.35--7.25) |
| Zimbabwe | 7531 (5472-9899) | 7380 (4943-10767) | -0.02 (-0.37-0.55) | 167.05 (121.12-219.54) | 95.7 (63.92-138.33) | 1.19 (-0.29-2.69) |

Abbreviations: DALY: disability-adjusted life-year; ASR: age-standardized rate; EAPC: estimated annual percentage change; CI: confidence interval; UI: uncertainty interval
